# V_H_H antibody loop guides design of a synthetic macrocyclic peptide that potently blocks influenza virus membrane fusion

**DOI:** 10.1038/s44298-025-00166-1

**Published:** 2025-12-18

**Authors:** Rameshwar U. Kadam, Jarek Juraszek, Boerries Brandenburg, Divita Garg, Xueyong Zhu, Mandy Jongeneelen, Wim B. G. Schepens, Bart Stoops, Jan Vermond, Wouter Goutier, Chan Tang, Sven Blokland, Ronald Vogels, Robert H. E. Friesen, Maria J. P. van Dongen, Ian A. Wilson

**Affiliations:** 1 Department of Integrative Structural and Computational Biology, The Scripps Research Institute, La Jolla, CA USA; 2 Johnson & Johnson Innovative Medicine, Leiden, The Netherlands; 3 Johnson & Johnson Innovative Medicine, Beerse, Belgium; 4 The Skaggs Institute for Chemical Biology, The Scripps Research Institute, La Jolla, CA USA; 5 Present Address: Structural & Protein Science, Johnson & Johnson Innovative Medicine, San Diego, CA USA; 6 Present Address: Avidicure, Oegstgeest, The Netherlands; 7 Present Address: Onco3R Therapeutics, Leuven, Belgium; 8 Present Address: Allegria Therapeutics, Basel, Switzerland

**Keywords:** Biochemistry, Biophysics, Biotechnology, Computational biology and bioinformatics, Drug discovery, Structural biology

## Abstract

Miniaturizing biologically complex structural motifs to produce synthetic functional mimetics holds significant promise for development of new therapeutic modalities. Here, we demonstrate a unique approach using the key binding loop of the single variable domain of a heavy chain (V_H_H) llama antibody as a starting point for peptide design. V_H_H antibodies of camelids and sharks generally have longer, but more ligand-efficient complementarity determining region 3 (CDR3) loops and are relatively stable structures. We harnessed these attributes as templates for design of a series of synthetic macrocyclic peptides. The designed peptides exhibit nanomolar binding to influenza hemagglutinin (HA) and heterosubtypic in vitro neutralization breadth against influenza A viruses by inhibiting the low pH mediated HA conformational changes that lead to membrane fusion. X-ray structures of peptide-HA complexes reveal high structural mimicry with the parent V_H_H antibody. One such macrocycle peptide candidate is promising for further development of broad protection against influenza A group 1 viruses.

## Introduction

The improved understanding of biological pathways and disease targets in the post-genomic era has opened up a diverse target space for potential therapeutics^1,2^. A substantial number of these unexplored targets constitute topologically complex, flat and large binding surfaces that are difficult to target with conventional drug-like small molecules^3–6^. Consequently, biologics (protein-based therapeutics), particularly antibodies (Abs) and Fc-fusion proteins, are becoming exceedingly important^7–9^. Although antibodies provide high affinity and specificity against such challenging targets, they generally lack cell permeability, have restricted bioavailability, may be thermally unstable, and are expensive to manufacture, thereby limiting their broad applicability^7,10^. Notwithstanding, antibodies are currently among most successful therapeutics for certain indications^7,9,11–13^.

Deconstructing biologics to produce stable, functional and bioavailable designs without losing specificity and affinity towards a specific target is an arduous task and various approaches are being explored^14–21^. Conventional as well as heavy-chain only antibodies naturally produced by camelids and sharks have been deconstructed to Fab (Fragment antigen binding), Fc (Fragment crystallizable region), and V_H_H fragments for a variety of therapeutic applications^22,23^. Alternatively, engineered non-immunoglobulin (Ig)^10^ based protein scaffolds (~6–20 kDa) e.g., adnectins^24^, affibodies^25^, affitins^26^, alphabodies^27^, anticalins^28^, centyrins^29^, DARPins^30^, Kunitz domains^31^, monobodies^32^ and other small proteins^33–36^ are also being explored. However, many of these engineered biologics pose significant challenges for further clinical development such as immunogenicity, short serum half-life, and confined bioavailability^37^.

Peptidic ligands occupy an intermediate space between antibodies and small molecules; they combine high affinity, selectivity, and functional activity with the potential to address limitations in cell permeability, bioavailability, toxicity, and manufacturing cost. Nevertheless, peptides often exhibit short serum half-lives due to proteolytic degradation and rapid renal clearance. Extending systemic exposure could therefore enhance their therapeutic utility. Approaches successfully used for engineered biologics such as albumin-binding motifs, PEGylation, Fc fusions, and lipidation have prolonged circulating half-life and may be applicable to peptides, provided that potency, solubility, and tissue distribution are carefully balanced^38^. Multiple bioactive peptides and peptidomimetics based on the variable domains of antibodies have been reported^39–50^. The CDR loops from light (V_L_) or heavy (V_H_) chains and framework region 3 (FR3) have either been utilized individually^40–42,44–46,50^, or features from multiple loops have been merged into a single design^17,39^. These loops have been selected from overlapping peptide fragments combinatorically^40,48^ or based on known antibody-antigen interactions^17,44,47^. Notwithstanding, further opportunities for greater success in rational design of therapeutics from antibodies seems possible.

Here we report a structure-guided methodology for design of cyclic peptides that target topologically complex binding sites, such as the HA stem region once thought to be undruggable. For the design of these cyclic peptides, we were specifically inspired by single domain antibodies (sdAbs) (also known as nanobodies or V_H_H antibodies), which are naturally produced in camelids and sharks. Despite lacking a light chain, these sdAbs maintain full antigen binding capability that often utilize much longer CDR3 loops compared to conventional V_H_ domains^51–53^. Our approach is illustrated through the development of a series of potent peptides that combine the key interactions and functionality of broadly neutralizing llama sdAbs against influenza HA such as sdAb38^54^. The HA interacting region of the CDR3 loop of this sdAb was identified from the crystal structure of the sdAb38-HA complex^54^ and constrained with dipeptide templates to generate a small cyclic peptide library. Subsequently, these designs were synthesized and tested in vitro for binding and neutralization activity against influenza virus, and a subset was structurally characterized. The most promising peptide, CP1, was further evaluated to determine its mechanism of action and to validate its translational potential.

## Results

### sdAbs as a template for design of peptide modalities

Among the CDRs of heavy and light chains of antibodies, the heavy chain CDR3 (HCDR3) is known to be the most distinct^55–57^. HCDR3 loops show wide diversity in size, shape, and composition within and across different species. To explore the diversity of HCDR3 loops and analyze their potential to develop into therapeutic modalities, we performed comparative analysis of HCDR3 loops of 28 different randomly selected Abs and CDR3 loops of sdAbs against various targets (Supplementary Fig. 1 and Supplementary Table 1). Our analysis shows that on average CDR3 loops of sdAbs are 4 and 7 amino acids longer compared to human and mouse HCDR3 loops, respectively, and contribute an average of ~67% of the heavy atom contacts with target antigens (Supplementary Fig. 1 and Supplementary Table 1). Conventional Abs show relatively smaller HCDR3 loops and ~36% fewer heavy atoms contacts, with on average only 4 residues in direct contact with the antigen binding site (Supplementary Fig. 1 and Supplementary Table 1), although with some exceptions including Abs PG9^58^, C05^59^ and PGT145^60^, which have longer HCDR3 loops. The long CDR3 loop in sdAbs with >38% contribution to the interaction with the target antigen have relatively high propensity to form stable and well-ordered structures and can be stabilized in the absence of antibody framework^61,62^. Thus, the relatively large length and major interactions of CDR3 loop residues in sdAbs as compared to Abs towards the target antigen could act as an ideal template for designing peptide-based modalities. Bovine antibodies would be another alternative with exceptional ultralong HCDR3 loops of 50–61 amino acids. However, the unusual size and shape of the stalk and knob region of the bovine HCDR3 with a number of cysteine residues with different disulfide bond patterns make them more demanding templates for development of peptide-based modalities^57,63^.

### sdAb paratope mimetic design strategy

**Fig. 1:**
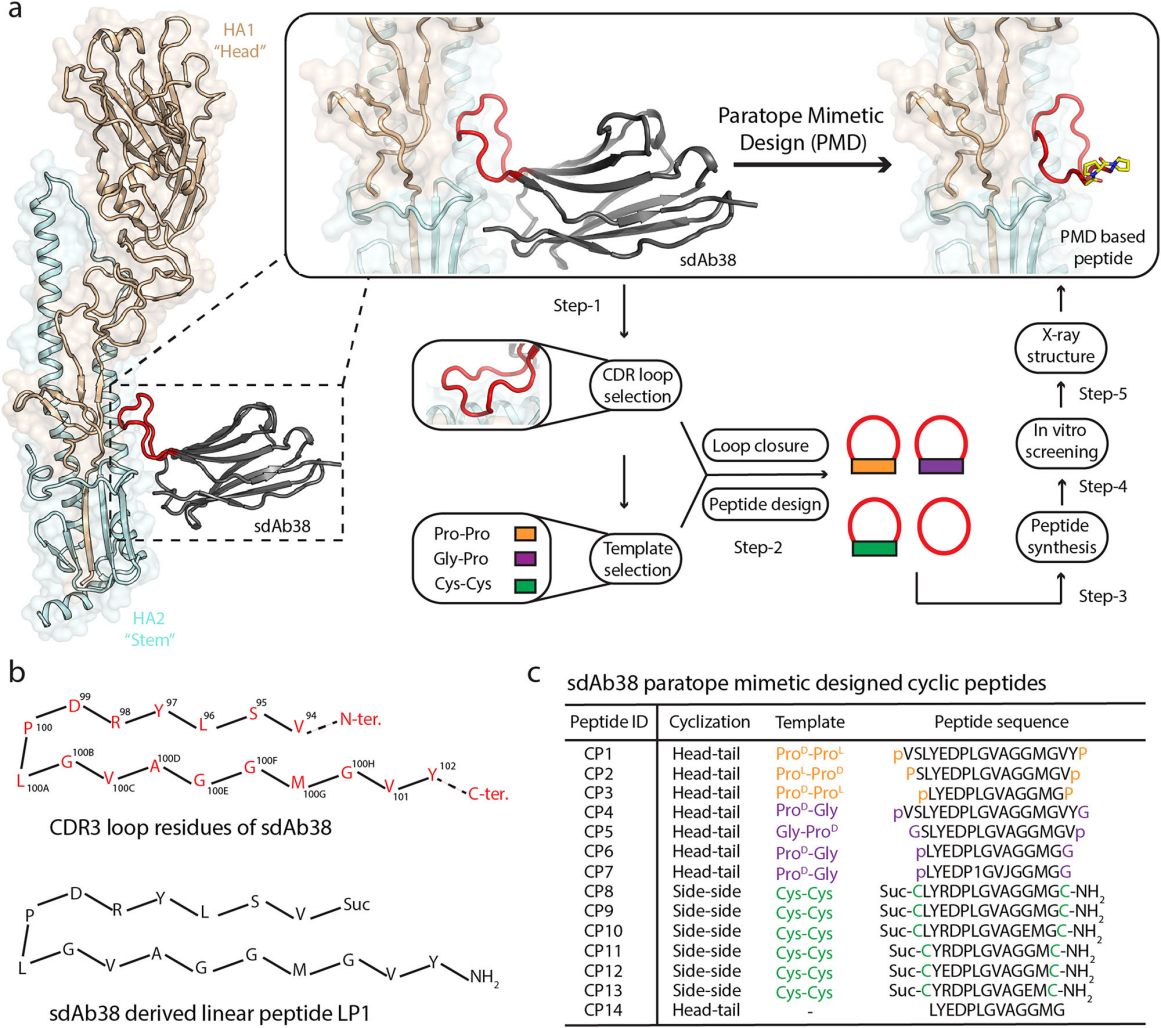
Blueprint for sdAb paratope mimetic design (PMD). **a** Complete cycle of PMD-based peptide design. Crystal structure of influenza HA from group 1 H1N1 A/Solomon Islands/3/2006 (H1/SI06) (HA1-beige and HA2- light cyan cartoon) in complex with single domain antibody sdAb38 (black) with HA-interacting CDR3 loop (red) (PDB ID 6FYT)^54^. One protomer of the H1/SI06 trimer is represented. The selected CDR3 loop is in (red) and the dipeptide templates are in orange, purple, and green. The designed peptides are represented as a combination of red loops grafted on templates (rectangles of different colors). The complete red circle represents a peptide without template. **b** 2D-representation of amino acid sequence of CDR3 loop of sdAb38 and the loop-derived linear peptide (LP1). **c** Designed cyclic peptides (CP1-CP13) based on sdAb38 and the dipeptide templates [proline-proline (Pro^D^-Pro^L^ or Pro^L^-Pro^D^, orange); proline-glycine (or Pro^D^-Gly or Gly-Pro^D^, purple); cysteine-cysteine (Cys^L^-Cys^L^, green)]. Peptide CP14 is non-template-based cyclic peptide. Non-proteinogenic amino acids in peptide CP7 are (1): 2-amino-5-phenylpentanoic acid and (J): D-homoalanine. p & P stand for D-proline and L-proline; subscripted L & D stand for L- and D- isomeric forms of amino acids; Suc- succinic acid; NH_2_-amine; Ter.- terminal.

Our structure-based sdAb paratope mimetic design (PMD) approach consisted of five steps, which are illustrated in Fig. 1 (1) selection of the major interacting CDR loop from the antigen-single-domain antibody complex; (2) design of cyclic peptides by selection of the dipeptide template and grafting of the loop onto the template; (3) synthesis of hybrid peptide variants; (4) in vitro screening against the target antigen; and (5) structural, mechanistic and translational characterization of the most promising peptide candidates. The applicability of this approach was tested here against influenza A virus HA.

### Design of influenza HA stem binding peptides

**Fig. 2:**
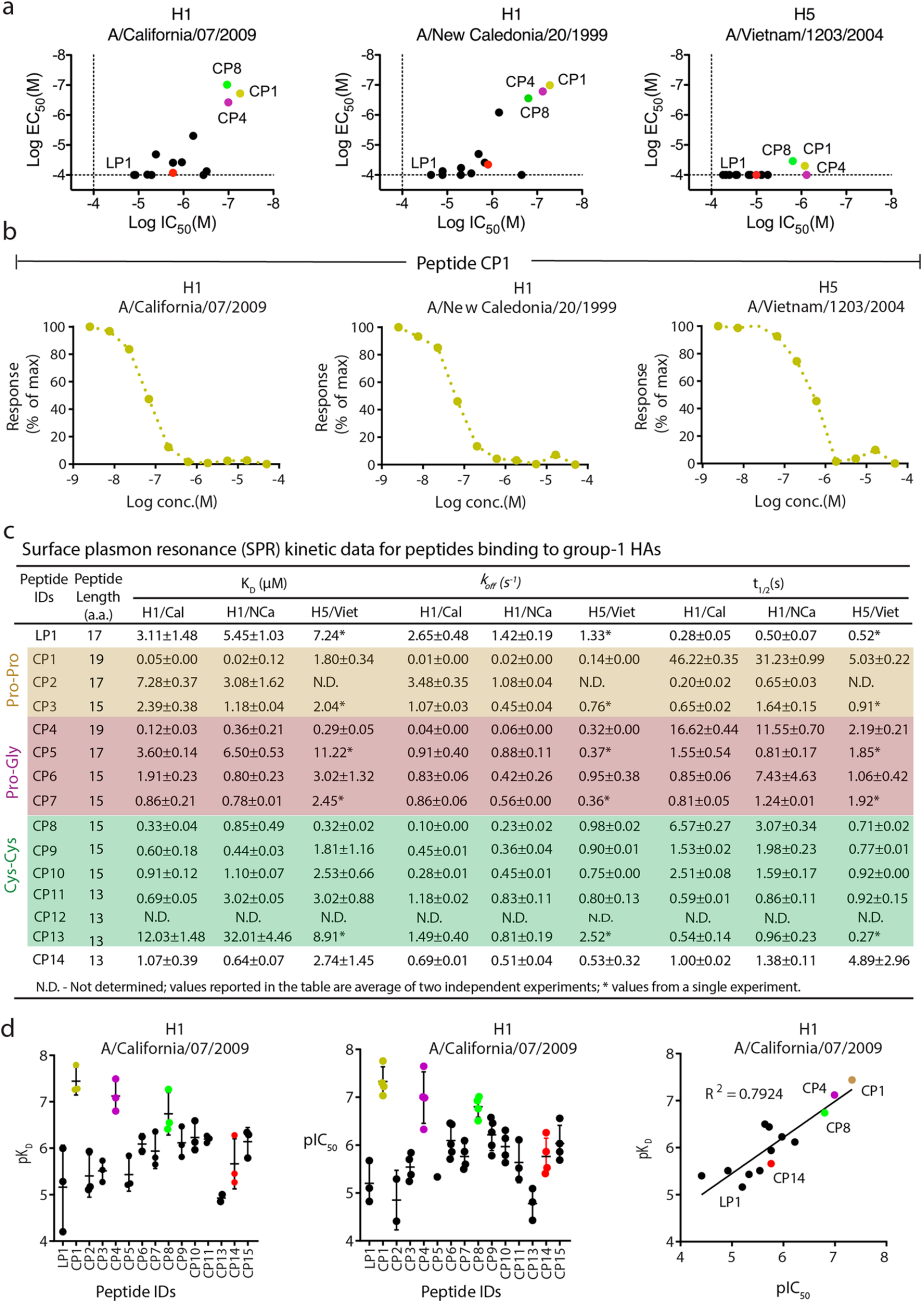
In vitro binding, kinetics and neutralization data for PMD based peptides. **a** Scatter plot depicting peptide-mediated binding potencies determined using an AlphaLISA competition binding assay (ALC), reported as log IC_50_ in molar (*x*-axis) *versus* neutralization activity determined using virus neutralization assay (VNA), reported as log EC_50_ in molar (*y*-axis). ALC and VNA assays were performed using H1N1 A/California/07/2009 (H1/Cal), H1N1 A/New Caledonia/20/1999 (H1/NCa), and H5N1 A/Vietnam/1203/2004 (H5/Viet) HAs and corresponding H1N1 and H5N1 virus strains respectively. Competition against the HA stem-binding protein HB80.4^34^ and Fab of HA head binding antibody 2D1^80^ were performed in the ALC assay. Each dot represents one tested peptide. The most potent peptides are highlighted as CP1, yellow; CP4, magenta, and CP8, green, whereas the non-template-based peptide (CP14, red) and other weak binding peptides are represented as black dots. Peptide CP5 was not included in the analysis. The dotted line represents the lower limit of quantification in the assays. **b** Dose-dependent competitive binding curves for peptide CP1 binding to the H1/Cal, H1/NCa and H5/Viet HAs, respectively. HA stem binding small protein HB80.4 and Fab of HA head binding antibody 2D1 used in the ALC assay. Peptide concentrations are reported in log molar (*x*-axis) versus % normalized binding response (*y*-axis). **c** Surface plasmon resonance (SPR) derived kinetic data for the peptides, as determined for H1/Cal, H1/NCa and H5/Viet HAs. Peptide half-life is calculated as *t*_1/2_ = ln 2/ *k*_off_. Values reported in the table are an average of two independent experiments. Errors are calculated as uncertainty in the mean, $\Delta {{\mathrm{x}}}_{{avg}}=R / (2\sqrt{N})$; where, *x* = experimental data values, *R*= difference between the maximum and minimum values of *x*. *N* = number of measurements. SPR measurements were not performed for peptide CP12. SPR response is measured in response units (RU). **d** Statistical comparison of binding data of peptides in surface plasmon resonance (SPR) and AlphaLISA assay.

**Fig. 3:**
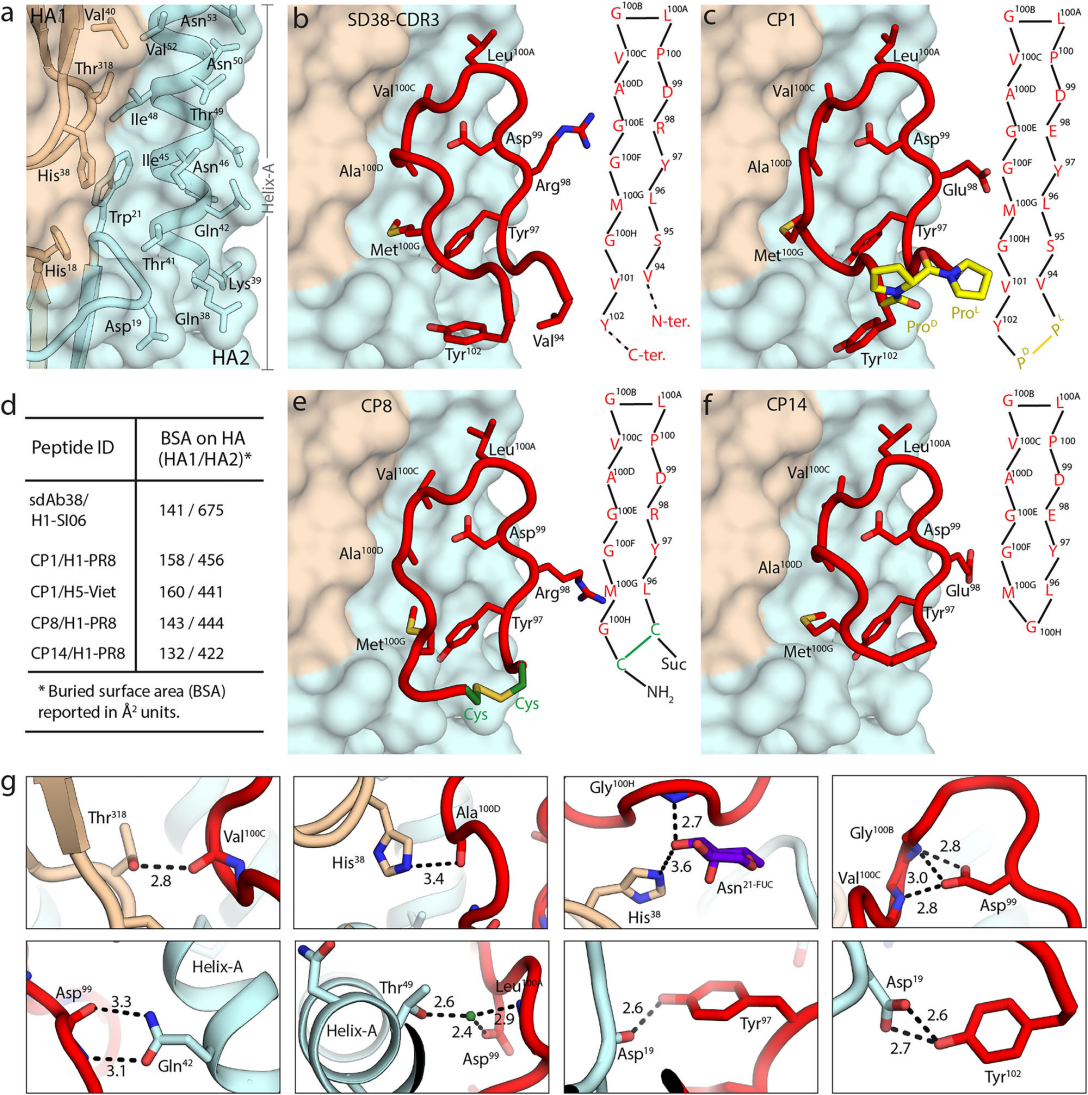
Structural elucidation of peptide binding to influenza HA. **a** Molecular surface of H1/SI06 HA (PDB ID 6FYT) (HA1- beige and HA2- light cyan; see Fig. 1a for overall location). **b** Binding mode of interacting CDR3 loop residues of sdAb38 on H1/SI06 HA (PDB ID 6FYT). **c** Binding mode of peptide CP1 in complex with H1/PR8 on H1/SI06 HA after superimposing (PDB ID 6FYT). **d** Surface area buried by sdAb38 and designed peptides on HA. Binding modes of peptides CP8 (**e**) and CP14 (**f**) in complexes with H1/PR8 on H1/SI06 HA after superimposing (PDB ID 6FYT). Peptide macrocycle is represented as a backbone tube with side chains as sticks (red) with Pro and Cys templates in yellow (CP1) and green (CP8), respectively. Corresponding 2D-represention and sequence is shown for the CDR3 loop of sdAb38 and peptides. Numbering of the peptide residues is retained with that of the CDR3 loop of sdAb38 (Fig. 3b). **g** Non-covalent interactions of peptide CP1 in complex with H1/PR8 HA. Peptide- main-chain tube with side-chain sticks (red), water molecule- green sphere, and fucose residue in *Asn*^*21*^ glycan- purple sticks (for clarity only the fucose residue from the *Asn*^*21*^ glycan is shown), and HA1- beige and HA2- light cyan. Hydrogen bond distances are measured in angstroms (Å).

Previously, we isolated and characterized a potent camelid single domain antibody, sdAb38, that targets HAs from influenza A group 1 viruses [e.g., SPR K_D_ of 3 nM against A/Brisbane/59/07(H1N1)(H1/Bris) and microneutralization IC_50_ of 2 nM against A/California/07/09 (H1N1)(H1/Cal)] and, to a lesser extent, group 2 viruses [(K_D_ of 44 nM against A/Hong Kong/1/68(H3N2) and IC_50_ of 210 nM against A/Brisbane/10/07 (H3N2)]^54^. The crystal structure of sdAb38 in complex with group 1 H1N1 A/Solomon Islands/3/2006 (H1/SI06) HA^54^ established that the 17-residue CDR3 loop of sdAb38 serves as the dominant interacting motif and recognizes a highly conserved epitope at HA1/HA2 interface in the stem region of HA (Fig. 1a). Furthermore, the single hairpin loop with its intramolecular hydrogen-bonding network appeared to afford considerable conformational stability. The CDR3 loop sequence of sdAb38 was therefore selected as the starting point for peptide design (Fig. 1b). Design and synthesis of a 17-residue linear peptide LP1 was followed by design of cyclic peptides in which the CDR3 loop was constrained by covalent cyclization. We started by stabilizing longer peptides, containing four amino-acids of the framework, on proline-proline (Pro^D^-Pro^L^ or Pro^L^-Pro^D^)^61,62^ and proline-glycine (Pro^D^-Gly or Gly-Pro^D^) dipeptide templates that are prone to form β-turns. Cysteine-cysteine (Cys^L^-Cys^L^) dipeptide templates were also used as an alternative cyclization strategy that would allow modification of the peptide properties via N- or C-terminal appendages. In the next steps, framework amino acids were removed in pairs, and the same strategy was repeated, ensuring that the introduced turns did not clash with the remaining amino acids or target protein. Finally, direct head-to-tail cyclization was attempted. A small library of cyclic peptides (CP1-CP14) and a linear peptide (LP1) were synthesized using standard solid phase peptide synthesis and characterized by liquid chromatography-mass spectrometry (LC-MS) (Fig. 1b, c, and Supplementary Fig. 2). Peptides were cyclized either by head-tail lactam formation or by sidechain-sidechain disulfide bond formation (Fig. 1c and Supplementary Fig. 2). In the designed peptides, the Arg residue in CDRH3 of sdAb38 was replaced by a Glu residue, with the aim of improving binding, by enhancing shape complementarity with Asn46 of HA (Figs. 1b, c, 2 and 3), and peptide solubility (pI 5.93 to more acidic pI).

### In vitro binding, kinetic and virus neutralization

Peptides were tested in vitro for binding affinity by surface plasmon resonance (SPR), binding potency in an AlphaLISA competition assay (ALC), and neutralization efficacy in a virus neutralization assay (VNA) against HAs from a broad variety of influenza viruses, i.e., influenza A group 1 H1/Cal, H1N1 A/New Caledonia/20/1999 (H1/NCa) and H5N1 A/Vietnam/1203/2004 (H5/Viet)), group 2 H3/Bris and H7N7 A/Netherlands/219/2003 (H7/Neth)), and influenza B (B/Florida/4/2006) (B/Flo)). The representative panel of HAs in the binding assay captures represents a majority of the observed group 1 and group 2 sequence variation in the HA stem epitope.

Linear peptide LP1 displayed weak micromolar level binding to group 1 H1/Cal, H1/NCa, and H5/Viet HAs (K_D_ of 3–7 µM by SPR; IC_50_ of 11–47 μM in AlphaLISA) with a fast dissociation rate constant (*k*_off_ = 1.3–2.6 s^-1^), and short half-life (*t*_1/2_) of 0.2–0.5 s (Fig. 2a–d, Supplementary Figs. 3–5, and Supplementary Tables 2–4). Consistent with sdAb38, which showed much weaker or no potency towards group 2 and influenza B viruses, no binding was observed to HAs from these viruses (Supplementary Figs. 3 and 4, and Supplementary Table 2). However, in comparison to the linear peptide, the cyclic peptides with Pro^D^-Pro^L^ (CP1), Pro^D^-Gly (CP4), and Cys-Cys (CP8) templates showed improved binding, kinetics, and neutralization activity against HAs (Fig. 2a–d, Supplementary Figs. 5 and 6, and Supplementary Tables 2–4). In particular, the 19-residue D-proline-L-proline based peptide (CP1) showed significant 60-fold improvement in binding affinity (*K*_D_ of 50 nM by SPR) towards H1/Cal and 60- to 440-fold improvement in binding in AlphaLISA (IC_50_ of 0.052–0.84 μM) against H1 and H5 HAs (Fig. 2a–c, and Supplementary Table 2). Binding of CP1 also resulted in more stable complex formation as compared to LP1, showing 9- to 180-fold slower dissociation (*k*_off_) or longer half-life (*t*_1/2_) of 5–46 s (Fig. 2c), with good neutralization activity against H1N1 and modest activity against H5N1 viruses (Fig. 2a and Supplementary Table 3). The 19-residue CP1 appeared to be optimal in the Pro^D^-Pro^L^ series, as shorter variants with 17 or 15 residue cycles (CP2 and CP3, respectively), and swapping the template Pro^D^-Pro^L^ with Pro^L^-Pro^D^ (CP2) resulted in a 50- to 130-fold decrease in binding and a corresponding 22- to 348-fold faster dissociation rate compared to CP1. These findings rationalized the decrease or complete abolition of neutralization activity of other proline-template cyclized peptides compared to CP1 (Fig. 2a–d, and Supplementary Table 3).

The variation in template from proline-proline to proline-glycine for the same sequence and number of amino acids in peptides CP1-CP3 *vs* the corresponding CP4-CP6 designs, did not result in any significant change in binding against H1 HAs (Fig. 2a, c, and Supplementary Table 2). The HA-CP4 complex was also less stable compared to the CP1 complex, with 2- to 3-fold higher dissociation rate constants (*k*_off_ = 0.04–0.32 s^−1^) and correspondingly 2- to 3-fold reduced residence time (*t*_1/2_ = 2–16 s) against H1 and H5 HAs (Fig. 2c). These kinetic parameters could explain complete abolishment of neutralization activity against H5N1 virus by CP4 (Fig. 2a, c, and Supplementary Tables 2 and 3). Interestingly, decreasing the peptide cycle length (CP1 vs CP3, CP4 *vs* CP6) reduced binding, potency and neutralization towards HAs (Fig. 2a–d, and Supplementary Tables 2 and 3). Substitution of **Leu**^**100A**^ and **Ala**^**100D**^ in peptide CP6 with non-proteinogenic bulky amino acids, 2-amino-5-phenylpentanoic acid (1) and D-homoalanine (J), respectively, in CP7, did not improve binding or potency, but abolished neutralization activity (Figs. 1c and 2c, and Supplementary Tables 2 and 3).

The cysteine-cysteine class of peptides (CP8-CP13) all have relatively short loops with 13–15 amino acid residues. Proline-proline and proline-glycine template-based peptides CP3 and CP6, respectively, have similar length (15 amino acid residues) and composition as the cysteine-cysteine template-based peptide CP8 except for the **Glu**^**98**^→**Arg**^**98**^ substitution in CP8. Compared to CP3, CP8 demonstrated a significant improvement in binding (K_D_ of 0.32–0.33 µM by SPR) and potency (IC_50_ of 0.11–1.55 μM in AlphaLISA) against H1/Cal and H5/Viet HAs (Figs. 1c and 2c, and Supplementary Table 2). The HA-CP8 complex was also more stable compared to the CP3 and CP6 complexes, with 2- to 11-fold slower dissociation rate constants (*k*_off_ = 0.1–0.98 s^−^^1^) against H1 HAs (Fig. 2c). However, compared to the longer loop peptides with proline-proline and proline-glycine templates (CP1 and CP4 respectively; loop length of 17 to 19 residues), CP8 had 3- to 36-fold reduction in binding against H1 HAs (Fig. 2a, c, and Supplementary Table 2). The HA-CP8 complex was also less stable compared to the CP1 and CP4 complexes, with 2- to 11-fold higher dissociation rate constants and correspondingly 2- to 10-fold reduced residence time (*t*_1/2_ = 0.7–6.5 s) against H1 and H5 HAs (Fig. 2c). Nevertheless, virus neutralization activity against H1N1 and H5N1 viruses was retained (Supplementary Table 3).

The data from the cysteine-cysteine containing peptides suggest that decreasing the loop length (15-residue peptides CP9 *vs* 13 residue peptides CP12) reduced binding and neutralization (Fig. 1c, and Supplementary Tables 2 and 3). Further, retention of the original **Arg**^**98**^ from the CDR3 loop of sdAb38 did not affect binding affinity as compared to peptides with the **Arg**^**98**^→**Glu**^**98**^ mutation (cf. K_D_ of CP8 vs CP9) and IC_50_ of CP11 vs CP12) (Figs. 1c and 2c, and Supplementary Table 2).

Although the D-proline-L-proline template has been well-characterized for constraining β-hairpin like turns in proteins^61,62^, the current study enhances our understanding of the application of this template for constraining long antibody loops (Fig. 3). Further, comparison of the cyclic peptides based on different templates suggests that D-proline-L-proline and cysteine-cysteine are more favorable templates for retaining binding and neutralization as compared to the D-proline-glycine template (Supplementary Figs. 5 and 6 and Supplementary Tables 2–4).

### Structural basis of paratope mimetic peptides binding to HA

To elucidate the structural basis of binding and neutralization of influenza virus by the PMD-based peptides, crystal structures of the neutralizing (template-based CP1 and CP8) and non-neutralizing (non-template based CP14) peptides in complex with H1/PR8 and H5/Viet HAs were determined at 1.59–2.87 Å resolution, respectively (Fig. 3, Supplementary Figs. 7–10, and Supplementary Tables 5 and 6). All peptides recognize the hydrophobic surface and embedded cavities at the interface of HA1-HA2 in the HA stem region (Fig. 3a). This region is highly conserved in HAs across influenza A groups 1 and 2 and influenza B viruses, and the target of HA stem broadly neutralizing antibodies (bnAbs)^64–66^, including sdAb38^54^. This region contains a number of small hydrophobic pockets within the HA1-HA2 interface, emulating protein-protein interface-like surfaces and features^6^ (Fig. 3a).

Peptides (CP1, CP8 and CP14) all mimic the binding mode of sdAb38 by occupying the same hydrophobic groove in the HA1/HA2 interface on HA (Fig. 3b–f), and bury on average ~148/440 Å^2^ surface on HA1/HA2 respectively, which is comparable to the surface area buried by sdAb38 (~141/675 Å^2^) (Fig. 3d). Molecular interaction analysis of the most potent peptide in the series, CP1, in complex with H1/PR8 HA revealed that **Tyr**^**97**^, **Asp**^**99**^, **Leu**^**100A**^, **Val**^**100C**^, **Ala**^**100D**^, **Met**^**100G**^ and **Tyr**^**102**^ (Fig. 3c) from the peptide macrocycle make a series of polar and non-polar contacts with the stem region residues HA1 *His*^*18*^*, His*^*38*^*, Val*^*40*^, and *Thr*^*318*^ (HA1 residues in italics throughout) and HA2 from Asp^19^ - Trp^21^, which form a beta turn, and helix-A residues Gln^38^ – Asn^53^. Polar interactions include direct and water-mediated hydrogen bonds and also interactions with a fucose on the N-linked glycan at *Asn*^21^ on HA1 (Fig. 3g). Backbone carbonyls of **Val**^**100C**^ and **Ala**^**100D**^ make direct hydrogen bond interactions with the side chains of *Thr*^*318*^ and *His*^38^, whereas the backbone amide of **Gly**^**100H**^ mediates hydrogen bond interactions with the *His*^38^ side chain via a fucose residue on the N-linked glycan at *Asn*^21^ (Fig. 3g).

The structure of CP1 bound to HA strongly mimics the intramolecular interactions of the sdAb38 CDR3 loop^54^. The **Asp**^**99**^ side chain is oriented towards the inside of the peptide macrocycle and makes intramolecular hydrogen bonds with backbone of **Gly**^**100B**^ and **Val**^**100C**^ to constrain the conformation of the peptide loop (Fig. 3b, c, g) and allows peptide recognition of HA2 helix-A via hydrogen-bond interactions with Gln^42^ and Thr^49^ (Fig. 3g). Further, **Tyr**^**97**^ occupies the same hydrophobic groove on HA as a conserved germline-encoded Tyr (D or J region) from HA-stem targeting *V*_*H*_*1-69* bnAbs CR9114 (**Tyr**^98^)^66^, CR6261 (**Tyr**^98^)^67^, 27F3 (**Tyr**^100a^)^68^, and F10 (**Tyr**^102^)^69^ (Fig. 3c and Supplementary Fig. 9). Similar to sdAb38, **Tyr**^**97**^ **and Tyr**^**102**^ make direct hydrogen bond interactions, respectively, with the backbone carbonyl and side-chain carboxyl of Asp^19^ from HA2 (Fig. 3g). Thus, this network of polar and non-polar interactions of peptide CP1 with HA1/HA2 residues acts as a molecular glue that holds the HA1/HA2 interface together and stabilizes the pre-fusion conformation of HA.

Next, we elucidated the structural basis for the effect of different templates. Crystal structures of peptides carrying the templates D-proline- L-proline (CP1), cysteine-cysteine (CP8) and the peptide without template (CP14), in complex with H1/PR8 HA were compared (Fig. 3c–f). All three peptides show similar binding modes, recapitulating the key interactions of sdAb38 with HA. Comparison of the buried surface area of the peptides demonstrated that especially CP1 preserved the attributes of sdAb38 in this respect, with 158 and 456 Å^2^ of the surface of H1/PR8 HA1 and HA2 buried.

To evaluate the structural basis of the decrease in binding and neutralization activity of CP1 against H5 HA versus H1 HA, we compared the crystal structures of CP1 bound to H1/PR8 and H5/Viet HAs (Supplementary Fig. 10). The presence of HA1 Val^40^ in the binding site of H1/PR8 HA allows for better steric accessibility of the peptide macrocycle, whereas the corresponding Gln^40^ in H5 has a relatively long and flexible side chain. The peptide macrocycle then moves ~1 Å away from binding site surface in H5/Viet HA, potentially contributing towards a 14-fold faster *k*_off_ rate and corresponding decrease in binding and neutralization activity of CP1 against H5 (Figs. 1c and 2c, Supplementary Fig. 10, and Supplementary Tables 2 and 3).

### HA binding specificity and mechanism of fusion inhibition

**Fig. 4:**
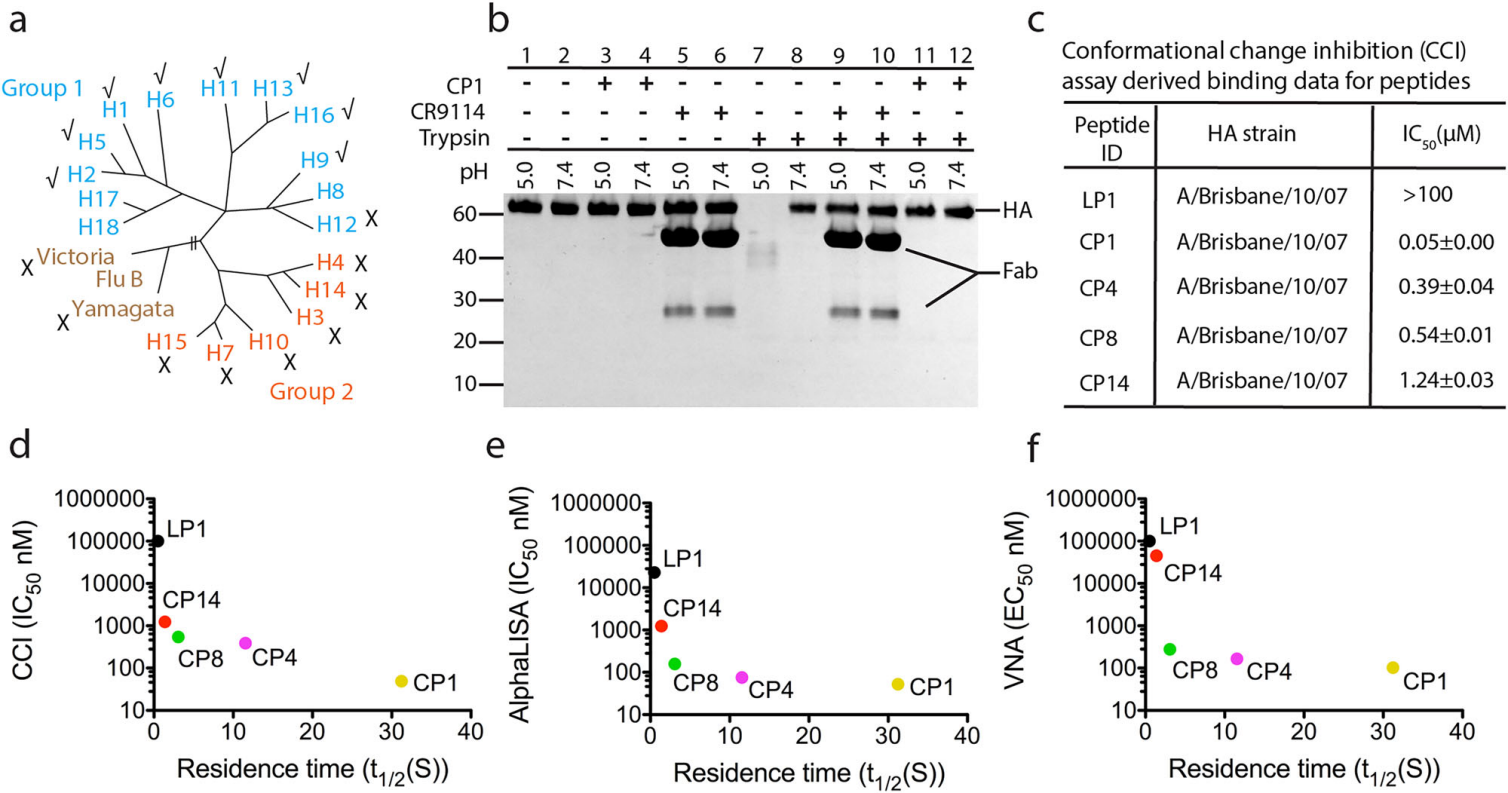
Breadth of binding, specificity and mechanism of fusion inhibition. **a** Phylogenetic tree representation showing the 18 HA subtypes of influenza A virus [group 1 (light blue) and 2 (orange)] and influenza B (brown). The breadth of binding of the peptide CP1 is marked as ($\surd$) and no binding as ($\times )$. Binding against H8, bat H17 and bat H18 HAs was not tested. **b** Trypsin susceptibility assay for peptide CP1 with H1/PR8. HA stem-binding antibody CR9114 Fab was used as positive control. Exposure to low pH renders the H1/PR8 HA sensitive to trypsin digestion (lanes 7 *vs* 8). CR9114 Fab (lanes 9 *vs* 7) and peptide CP1 (lanes 11 *vs* 7) protect HA by preventing its conversion to a trypsin-susceptible conformation. **c** Conformational change inhibition (CCI) assay derived IC_50_ values for peptides against H1N1 A/Brisbane/59/2007 (H1/Bri) HA. **d**–**f** Correlation of CCI, AlphaLISA, and VNA derived IC_50_ and EC_50_ respectively, with SPR-derived residence time t_1**/**2_. Peptides are represented as LP1 (black), CP1 (yellow), CP4 (magenta), CP8 (green) and CP14 (red) dots.

When tested against a broad panel of HAs in SPR, peptide CP1 shows heterosubtypic group 1 HA breadth and specificity (Fig. 4a and Supplementary Table 7), but no binding against group 2 or influenza B HAs. The major differences in the peptide binding site in group 1 H1 HA and the corresponding region on group 2 H3 HA are the presence of a glycosylation site at residue HA1 38, the orientation of Trp^21^, and a mutation in helix-A Thr^49^ in H1 HAs to Asn^49^ in H3 HAs. These key differences contribute to group 1 specific binding of bnAbs CR6261^67^, F10^69^ and the previously reported^17^ peptide P7, may also restrict CP1 specificity to group 1 HAs. As compared to the most potent known peptidic inhibitor^17^ (peptide P7) targeting a similar binding site in the HA stem, peptide CP1 shows an extended breadth across the group 1 HAs by binding additionally to H2, H5, H6, H9, H11, H13, and H16 HAs (albeit more weakly than to H1 HAs) (Fig. 4a, Supplementary Fig. 11, and Supplementary Table 7).

Since structural analysis of the PMD-based peptide-HA complexes revealed that these peptides mimic the binding mode of previously reported stem-targeting peptide P7, bnAbs, sdAb38 and others^17,54,65–67^, we proceeded to investigate if they also mimic their mechanism of action. After entry of the virus in the endosomal compartment, the low pH of the endosome triggers large conformational rearrangements in HA, which eventually lead to the post-fusion conformation. Peptide P7 and bnAbs stabilize the pre-fusion conformation of HA and prevent the significant rearrangement associated with the low pH-induced fusion process^17,65–69^.

First, crystals of peptide CP8 with H1/PR8 HA were obtained under low pH conditions (pH 4.2) (Supplementary Table 5). Although the crystallization pH is well below the pH of membrane fusion^70^, the structure of the H1/PR8 HA complex with CP8 is essentially identical to the pre-fusion apo form of H1/PR8 and H1/Cal HAs at pH 8.0 (Supplementary Fig. 12). This observation is consistent with the previously reported crystal structures of HA-antibody^65–67^and HA-peptide^17^ complexes obtained at low pH and that were also reported to inhibit fusion. We then performed a trypsin susceptibility (TS) assay, which measures the ability of inhibitors to prevent low pH-induced conformational changes in HA^71^. Peptide CP1 inhibits the conversion of H1/PR8 HA to the post-fusion conformation at pH 5.0, thereby preventing its susceptibility to trypsin cleavage (Fig. 4b, lane 11) that is similar to Fab CR9114 inhibition of HA conformational changes at low pH (Fig. 4b, lane 9)^66^.

Moreover, the conformational change inhibition (CCI) assay^17^ assesses dose-dependency of the inhibition of low pH induced HA conformational changes. All peptides described here, except linear peptide LP1, prevented conformational changes at low pH (Fig. 4c, and Supplementary Table 8). Further, the activity values derived from CCI, AlphaLISA and VNA correlate well with the kinetic parameters derived from SPR (Fig. 4d–f). The correlation with the kinetic data shows that peptides require on average 15 s of residence time to neutralize the virus. Once this residence time is achieved, the EC_50_ values do not significantly improve further (Fig. 4f). The cumulative data from these experiments imply that the peptides can indeed block the conformational changes associated with fusion of the influenza virus membrane with the host endosomal membrane.

### Translational properties of designed peptides

To investigate the suitability of the designed peptides for further preclinical and clinical development, we performed peptide cytotoxicity screen, and membrane permeability assays. The cytotoxicity screen was performed in the Cultured Human Airway Epithelial Cell line (Calu-3) (Supplementary Fig. 13). The cells were incubated at 37 °C with different concentrations of the peptides and cell viability was measured for up to 4 days. None of the designed peptides showed any signs of cytotoxicity in the Calu-3 cell line at the highest concentration of 100 µM (Supplementary Fig. 13).

To assess the P-glycoprotein (P-gp) substrate specificity and permeability across the membrane, peptides CP1 and CP14 were tested in vitro using a polarized monolayer of LLC-parent and LLC-MDR1 (multidrug resistance protein 1, also known as P-glycoprotein 1, P-gp) cell lines. Both peptides had efflux ratios <2 in the presence and absence of elacridar (GF120918), a well-known P-gp inhibitor (Supplementary Table 9), suggesting that both peptides are not substrates for P-gp. The analysis of membrane permeability of both peptides demonstrated low but measurable apparent transfer (P_app_) from apical to basolateral and reverse (Supplementary Table 9), thus revealing some permeability.

## Discussion

Here we report a unique, efficient and effective structure-based strategy to design peptidic modalities based on a single domain antibody. This strategy was used to graft the relatively long and flexible CDR3 loop of the single domain antibody sdAb38 onto dipeptide templates to design mimetics that can potently bind and neutralize influenza A viruses. We show that a 19-meric cyclic peptide based on a Pro^D^-Pro^L^ framework, CP1, demonstrated potent binding, kinetic and neutralization activity against many strains of group 1 influenza A viruses. Structural and functional characterization of CP1 and peptide variants in complex with HAs illustrate that these peptides mimic the binding mode and functionality of neutralizing antibodies by stabilizing the pre-fusion conformation. As a future optimization strategy to improve the cell permeability of these peptides, substituting nonessential polar side chains in the macrocyclic peptide (for example, replacing Glu^98^ with Leu) in combination with N-methylation of the backbone amides could be beneficial to potentially enhance peptide permeability^17,72,73^. Based on the data reported here, CP1 may be considered as a suitable candidate for further optimization and development of broad-spectrum therapeutics against influenza HA.

## Methods

### Peptide synthesis

All peptides were synthesized by manual solid phase Fmoc peptide chemistry. Rink Amide MBHA resin was used for C-terminal carboxamide linear peptides and 2-chlorotrityl chloride resin for head-to-tail lactam-cyclized peptides. Amino-acid side chain functionalities were protected as *N*-Boc (W), *O*-*t*-Bu (D,E,S,Y), *C*-*t*-Bu (C), and *N*-Pbf (R) groups (Boc: *tert*. Butoxycarbonyl, *t*-Bu: *tert*. Butyl, Fmoc: 9-Fluorenylmethoxycarbonyl, Pbf: 2,2,4,6,7-pentamethyldihydrobenzofuran-5-sulfonyl). Characterization of peptides was performed using high-performance liquid chromatography (HPLC) and mass spectrometry.

#### Synthesis of linear peptide

Linear peptide, LP1, was prepared by manual solid phase Fmoc chemistry on a Rink amide MBHA resin (0.53 mmol/g). The resin was swelled in DMF (dimethylformamide) for 1 h and treated with 20% piperidine in DMF (2 ×15 min) to effect Fmoc removal. All acylation reactions were carried out using threefold excess of Fmoc-amino acid activated with 0.95 eq. of HBTU ((2-(1H-benzotriazole-1-yl)-1,1,3,3-tetramethyluronium hexafluorophosphate) in presence of 2 eq. of DIPEA (*N*, *N*-diisopropylethylamine), with a coupling time of 1.5–2 h. *N*-terminal succinylation was performed by treating the peptide-resin with succinic anhydride (5 eq.) in presence of DIPEA (10 eq.) for 1 h. Peptide was cleaved from the resin and deprotected using trifluoroacetic acid/thioanisole/1,2-ethanedithiol/water (90:5:2.5:2.5) for 3 h. The resin was removed by filtration, after which the filtrate was poured into ice-cold *tert*-butyl methyl ether resulting in the precipitation of the crude peptide. Final purification was carried out using RP-HPLC (Reversed-Phase High Performance Liquid Chromatography).

#### Synthesis of head-to-tail lactam cyclized peptides

Linear peptides were prepared by manual solid phase Fmoc chemistry on glycine preloaded 2-chlorotrityl chloride resin (0.5 mmol/g) according to the protocol above. Peptides were cleaved from the resin by swirling the peptide-resin for 1 h in a mixture of dichloromethane /hexafluoroisopropanol/trifluoroethanol/triisopropylsilane (6.5:2:1:0.5). The resin was filtered off, and peptide precipitated from the filtrate by addition of cold *tert*-butyl methyl ether. The peptide was dried under vacuum and used as such in the subsequent lactam cyclization step. Lactam cyclization was performed at high dilution by dissolving the linear side-chain protected peptide in DMF (0.001 M), to which a solution of PyBOP (benzotriazol-1-yl-oxytripyrrolidinophosphonium hexafluorophosphate, 2 eq., 0.004 M) and *N*-methylmorpholine (6 eq., 0.012 M) in DMF was added dropwise. The reaction mixture was stirred at room temperature until complete conversion was observed. Removal of the solvent under reduced pressure afforded the cyclized peptide, which was completely deprotected by treatment with a mixture of trifluoroacetic acid/thioanisole/1,2-ethanedithiol/water (90:5:2.5:2.5) at room temperature for 3 h. The reaction mixture was poured into ice-cold *tert*-butyl methyl ether resulting in precipitation of the crude peptide. Final purification was done using RP-HPLC.

#### Synthesis of sidechain-sidechain lactam cyclized peptides

Disulfide cyclization was effected by iodine oxidation. The crude linear peptide precursor, prepared as described above, was dissolved in water/acetonitrile (7:3) and an iodine solution (0.1 M in methanol) was added until permanent discoloration of the reaction mixture was observed. Subsequent lyophilization afforded the crude cyclized peptide, which was purified using RP-HPLC. All linear and cyclic peptides were characterized using LC-MS (Supplementary Fig. 1).

### Expression and purification of the hemagglutinin for binding and crystallography studies

The HA ectodomain was expressed using a baculovirus expression system in Sf9 cells (Expres2ion Biotechnologies and Gibco)^17^. Briefly, each HA was fused with gp67 signal peptide at the N-terminus and to a thrombin, BirA biotinylation site, trimerization domain, and His-tag at the C-terminus. Expressed HAs were purified using metal affinity chromatography using Ni- NTA resin. For binding studies, HA0 was biotinylated with BirA (biotinylation reaction consist of 100 mM Tris pH 8.5, 10 mM magnesium acetate, 10 mM ATP, 50 µM biotin and <50 mM NaCl, at 37 °C for 1 h) and purified using size exclusion chromatography using a Superdex 200 16/90 column (GE Healthcare) with buffer (20 mM Tris-HCL, 150 mM NaCl at pH 8.0) on an ÄKTA protein purification system (GE Healthcare). Biotinylated HAs were concentrated to ~50 µg/ml and tested for biotinylation reaction by loading onto a streptavidin (SA) biosensor using Octet Red96 (Pall ForteBio) system and stored at −80 °C until the binding experiment. For crystallization studies, the HAs were purified by Ni-NTA affinity chromatography-based and then digested with trypsin (New England Biolabs, 5 mU trypsin per mg HA, overnight at 4 °C) to produce uniformly cleaved HA1/HA2, and to remove the trimerization domain and His-tag. The digested material was purified by gel filtration using the column and buffer composition as described above.

### Site-specific modification and labeling of the hemagglutinin for conformational change inhibition assay

The HA H1/Bri = A/Brisbane/59/07 (H1N1) HA was recombinantly expressed in Expi293F (Thermo Fisher Scientific) mammalian cells using a previous protocol^17^. Briefly, H1/Bri HA was expressed with a gp67 signal peptide, polyG (5xG) peptide sequence, and followed by three alanines, encoding for a NotI site, at the N-terminus, and to a fibritin trimerization domain, Sortase A recognition site (LPETG), and Histidine-tag (His_6_) at the C-terminus. Cells were transfected with a pCDNA2004 mammalian expression plasmid and the cell culture supernatant was harvested 7 days post transfection. The HA was purified by size exclusion chromatography using a Superdex 200 column (GE Healthcare) with a final formulation buffer of 20 mM Tris, 150 mM NaCl at pH 7.5 and used for CCI studies.

### AlphaLISA-based competition (ALC) binding assay

Peptides were dissolved at 10 mM in 100% DMSO and serially diluted 1:3 nine times in half-area 96-well plates. The peptides were further diluted 1:40 in assay buffer (PBS, 0.05% BSA, 0.05% Tween-20) and spun down for 15 min at 1000 g to separate any insoluble material. 10 µl of the supernatants were incubated for 1 h with 10 µl biotinylated HA (2.5 nM in assay buffer). Then, 10 µl of labeled binding protein (diluted in assay buffer) was added followed by another 1 h incubation. Subsequently, 10 µl of AlphaLISA acceptor beads (Perkin Elmer 50 µg/ml in assay buffer, specific for the binding protein label) was added and incubated for 1 h. Finally, 10 µl of streptavidin-coated donor beads (50 µg/ml in assay buffer) were added followed by 1 h incubation. Two solvent controls, with and without HA, were setup for each sample. Plates were read on a microplate reader at 615 nm.

### Virus neutralization assay (VNA)

Threefold serial dilutions of the peptides were prepared as described above. Cultured Human Airway Epithelial Cells (Calu-3) (ATCC) were seeded at least 1 day to maximum 10 days in advance in black, clear bottom, 96-well plates at 40,000 cells/well. On the day of the experiment, a 33.3× pre-dilution of the peptides was prepared in incomplete medium (DMEM, 1× L-glutamine, 1× pen/strep) and spun down at 1000 × *g* for 15 min to separate any insoluble material. After spinning, 50 µl supernatant was transferred to a pre-incubation plate followed by 50 µl of the respective virus dilutions prepared in the medium. Two solvent controls, with and without virus, were setup for each sample. Plates were incubated for 1 h at 37 °C. During incubation, the culture medium from the Calu-3 cells was replaced by 50 µl incomplete medium plus 3% heat-inactivated fetal bovine serum (FBS). Then, 100 µl of the virus/peptide mixture was added to the cells followed by incubation for 4 days at 37 °C under 10% CO_2_. After incubation, the supernatant was removed from the cells and 200 µl of ice-cold 80% acetone was added to fix the cells. After 15 min, the fixative was removed and the plates were air-dried for 20 min. Detection of virus nucleoprotein was performed using mouse anti-influenza A NP antibody in PBS, 1% BSA. After an hour, cells were washed followed by incubation with goat anti-mouse HRP-conjugated antibody in PBS with 1% BSA for 1 h. After another wash step, 50 µl BM Chemiluminescence ELISA Substrate (Roche) was added to the wells and the plate read for luminescence. Sd12-038 used as a positive control for group 1 influenza HA strains and sd12-036 for group 2 HA strains.

### Conformational change inhibition (CCI) assay

Threefold serial dilutions of the peptides were prepared as described above. Half-area high binding plates were coated overnight with 50 µl 0.5 µg/ml streptavidin in PBS. The plates were then washed thrice with 150 µl PBS, 0.05% Tween-20, and blocked by addition of 100 µl CCI assay buffer (PBS, 1% BSA, 0.1% Tween-20) per well. After overnight blocking, the plates were washed again followed by the addition of 50 µl C-terminal biotinylated H1/Bri HA (0.1 µg/ml in assay buffer) per well. Plates were incubated for 1 h on a plate shaker. A 100× dilution of the peptides was prepared in a 96-well plate by adding 2 µl of peptide to 198 µl assay buffer. Two controls were set up in the dilution plate, one of which received 198 µl of a positive control antibody (final concentration of 4 nM in assay buffer). After 1 h, the assay plate was washed and 50 µl was transferred from the peptide dilution plate to the assay plate followed by another 1 h on a plate shaker. Then 10 µl of 1 M acetate, pH 5.25, was added to all wells followed by 20 min on a plate shaker. The plate was washed followed by addition of 2.5 mM DTT in PBS for 1 h on a plate shaker after which the plate was washed and 0.5 µg/ml CH65-HRP (HRP-labeled antibody to the HA receptor binding site) added. The plate was washed and 50 µl of POD (Chemiluminescence ELISA Substrate) was added to the wells and read for luminescence on a microplate reader 5 min later.

### Peptide toxicity screen (PTS)

Threefold serial dilutions of the peptides were prepared as described above. Calu-3 cells were seeded at least 1 day to maximum 10 days in advance of the experiment in black clear-bottom 96-wells plates at 40,000 cells/well. On the day of the experiment, a 33.3× pre-dilution of the peptides was prepared in incomplete medium (DMEM, 1× L-glutamine, 1× pen/strep). Controls were added separately to the plate, which was then spun down at 1000 × *g* for 15 min. After spinning, 60 µl of the supernatant was transferred to a pre-incubation plate followed by 60 µl incomplete medium. Column 12 in the 96-wells plate received 60 µl incomplete medium plus 33.3% DMSO to kill the cells (low control). Plates were incubated for 1 h at 37 °C. During the incubation, the culture medium from the Calu-3 cells was replaced by 50 µl incomplete medium plus 3% heat inactivated FBS. Then 100 µl of the virus/peptide mixture was added to the cells followed by an incubation period of 4 days at 37 °C, 10% CO_2_. After 4 days, 70 µl of the supernatant was removed from the 96-wells plates and replaced by 70 µl ATPlite 1step Luminescence reagent (Perkin Elmer). The plate was read for luminescence. As controls, we included a negative control consisting of cells treated with vehicle alone (no DMSO) and blank wells to measure background. A positive control was provided by treating cells with 12% DMSO, a condition that yielded non-viable cells and defined the maximal cytotoxic response. Data were normalized to the negative control (100% viability) and interpreted relative to the positive control to establish assay dynamic range.

### In vitro cell permeability assay

Measurement of the apparent permeability as well as evaluation of P-gp substrate potential was performed in Lilly Laboratories Cell Porcine Kidney (LLC-PK1) parental cells and LLC-PK1-MDR1 (multidrug resistance protein 1, also known as P-glycoprotein 1, P-gp) cells stably transduced with the MDR1 transporter. These cells were seeded for 5 days for confluent cell monolayer formation using opti-MEM medium on 24-well cell culture inserts (Millicell^®^-PCF, 0.4 µm, 13 mm diameter, 0.7 cm^2^) at 400,000 cells/cm^2^. A known P-gp substrate (^3^H-Digoxin) and P-gp inhibitor (elacridar; GF120918), and peptides CP1 and CP14 were diluted with the transport buffer (1% BSA, PBS, pH 7.4) from a 5 mM stock solution to a concentration of 5 μM and applied to the apical (A) or basolateral (B) side of the cell monolayer. Permeation of the peptides from A to B or B to A direction was determined in triplicate over 120 min by incubation at 37 °C and with 5% CO_2_. Control compound samples were counted by Liquid Scintillation Counting and test peptides were quantified by LC-MS/MS and the efflux ratio (BA/AB) of control and each peptide were also determined and reported in Supplementary Table 9.

### Trypsin susceptibility (TS) assay

In the TS assay, 5 μM H1/PR8 = A/Puerto Rico/8/1934 (H1N1) HA was pre-incubated separately with 50 μM of peptide CP1 and 10 μM CR9114 Fab for 30 min at room temperature. Control reactions were incubated with 2% DMSO. The pH of each reaction was lowered using 1 M sodium acetate buffer (pH 5.0). One reaction was retained at pH 7.4 to assess any digestion at neutral pH. The reaction solutions were then thoroughly mixed and incubated for 20 min at 37 °C. After incubation, the reaction solutions were equilibrated at room temperature and the pH was neutralized by addition of 200 mM Tris buffer, pH 8.5. Trypsin-ultra™ (NEB Inc.) was added to all samples at final ratio of 1:50 by mass and the samples were digested for 30 min at 37 °C. After incubation with trypsin, the reaction solutions were equilibrated at room temperature and quenched by addition of non-reducing SDS buffer and boiled for 2 min at 100 °C. All samples were analyzed by 4–20% SDS-PAGE gel and imaged using BioRad ChemDoc™ imaging system.

### Surface plasmon resonance (SPR)

All SPR experiments were performed using a Biacore T200 instrument operating at 25 °C. For peptide binding to HA, biotinylated HA was covalently immobilized on a streptavidin-coated, carboxymethylated dextran sensor surface (SA chip, GE Healthcare). Peptides were dissolved at 10 mM in 100% DMSO and then diluted in the running buffer (20 mM PBS, 137 mM NaCl, 0.05% P-20 surfactant, pH 7.4 (GE Healthcare), supplemented with 2% DMSO). Binding constants were obtained from a series of injections of peptides from 0.1 nM to 10 µM with a flow rate of 30 µl/min. Data from single-cycle kinetics were analyzed using BIAevaluation software. Baselines were adjusted to zero for all curves, and injection start times were aligned. The reference sensorgrams were subtracted from the experimental sensorgrams to yield curves representing specific binding followed by background subtraction (i.e., double-referencing). Binding kinetics were evaluated using a 1:1 binding model (Langmuir) to obtain association rate constants (ka) and dissociation rate constants (kd). Binding affinity (K_D_) was estimated from the concentration dependence of the observed steady-state responses.

### Crystallization and structure determination of the peptide-PR8 HA complexes

Gel filtration fractions containing H1 A/Puerto Rico/8/1934 (H1/PR8) and H5 A/Vietnam/1203/2004 (H5/Viet) HAs were concentrated to ~10–11 mg/mL in 10 mM Tris, pH 8.0, and 100 mM NaCl. Peptides at ~5 molar excess were incubated with H1/PR8 HA and H5/Viet HA for 1 h at room temperature and centrifuged at 14,000 × *g* for ~30 s before crystallization. Crystallization screens were set up using the sitting drop vapor diffusion method with our automated CrystalMation robotic system (Rigaku) at The Scripps Research Institute. Within 3–7 days, diffraction quality crystals had grown. The resulting crystals were cryoprotected by addition of 5–15% ethylene glycol, and flash cooled and stored in liquid nitrogen until data collection. Diffraction data were collected at 100 K on the NIH General Medicine and Cancer Institute Collaborative Access Team (GM/CA-CAT) 23-IDD beamline at the Advanced Photon Source at the Argonne National Laboratory, and at Stanford Synchrotron Radiation Lightsource beamline 12-2. The diffraction data were processed with HKL-2000^74^. Initial phases were determined by molecular replacement using Phaser^75,76^ with an HA model from PDB codes 1RU7 (H1 HA) and 4FQI (H5 HA). Refinement was carried out in Phenix^77^, alternating with manual rebuilding and adjustment in COOT^78^. Data collection and refinement statistics are summarized in Supplementary Table 6. The final coordinates were validated using MolProbity^79^.

### Structural analyses

The surface area on the HA buried upon peptide binding was calculated with the Protein Interfaces, Surfaces and Assemblies (PISA) server at the European Bioinformatics Institute. MacPyMol (DeLano Scientific) and Bioware (http://bioware.ucd.ie/~cyclops/cgi-bin/webpep.cgi) were used to render the structure figures.

### Reporting summary

Further information on research design is available in the Nature Research Reporting Summary linked to this article.

## Supplementary information

Supplementary Information

Reporting Summary

## Data Availability

The x-ray coordinates and structure factors for the peptide complexes with H1/PR8 and H5/Viet HAs have been deposited with the Protein Data Bank under accession codes CP1-H1/PR8 (9PHS); CP8-H1/PR8 (9PHD); CP14-H1/PR8 (9PHQ) and CP1-H5/Viet04 (9PHT). All other data are in the manuscript or Supplementary Information.
