# Supplementary material for: VHH antibody loop guides design of a synthetic macrocyclic peptide that potently blocks influenza virus membrane fusion
Source: Npj Viruses. 2025 Dec 18;3:83. doi: 10.1038/s44298-025-00166-1 (PMC12714728; doi:10.1038/s44298-025-00166-1)
Supplement: Supplementary file 1 — Supplementary Information [file 44298_2025_166_MOESM1_ESM.pdf]

V$_{H}$H antibody loop guides design of a synthetic macrocyclic peptide that potently blocks influenza virus membrane fusion

Rameshwar U. Kadam[1§±*], Jarek Juraszek[2§^], Boerries Brandenburg[2], Divita Garg[1], Xueyong Zhu[1], Mandy Jongeneelen[2], Wim B.G. Schepens[3], Bart Stoops[3#], Jan Vermond[2], Wouter Goutier[2], Chan Tang[2], Sven Blokland[2], Ronald Vogels[2], Robert H. E. Friesen[2^], Maria J.P. van Dongen[2,3&] and Ian A. Wilson[1,4 *]

[1] Department of Integrative Structural and Computational Biology, The Scripps Research Institute, La Jolla, CA 92037, USA.

[2] Johnson & Johnson Innovative Medicine, 2333 CN Leiden, Netherlands.

[3] Johnson & Johnson Innovative Medicine, 2340 Beerse, Belgium.

[4] The Skaggs Institute for Chemical Biology, The Scripps Research Institute, La Jolla, CA 92037, USA.

[±]Current affiliation: Structural & Protein Science, Johnson & Johnson Innovative Medicine, San Diego, CA, 92121, USA.

[^]Current affiliation: Avidicure, 2342 DH Oegstgeest, Netherlands.

[#]Current affiliation: Onco3R Therapeutics, 3001 Leuven, Belgium

[&]Current affiliation: Allegria Therapeutics, 4051 Basel, Switzerland.

[§] These authors contributed equally to this work.

[*] Corresponding authors. E-mail: rkadam3@its.jnj.com (R.U.K); wilson@scripps.edu (I.A.W.)

Supplementary Information:

Supplementary Figures 1 to 14

Tables 1 to 9

References (1-3)

Supplementary Fig. 1. Comparative analysis of the CDR3 loop of antibodies (Abs) and single domain antibodies (sdAbs) against a diverse set of targets.

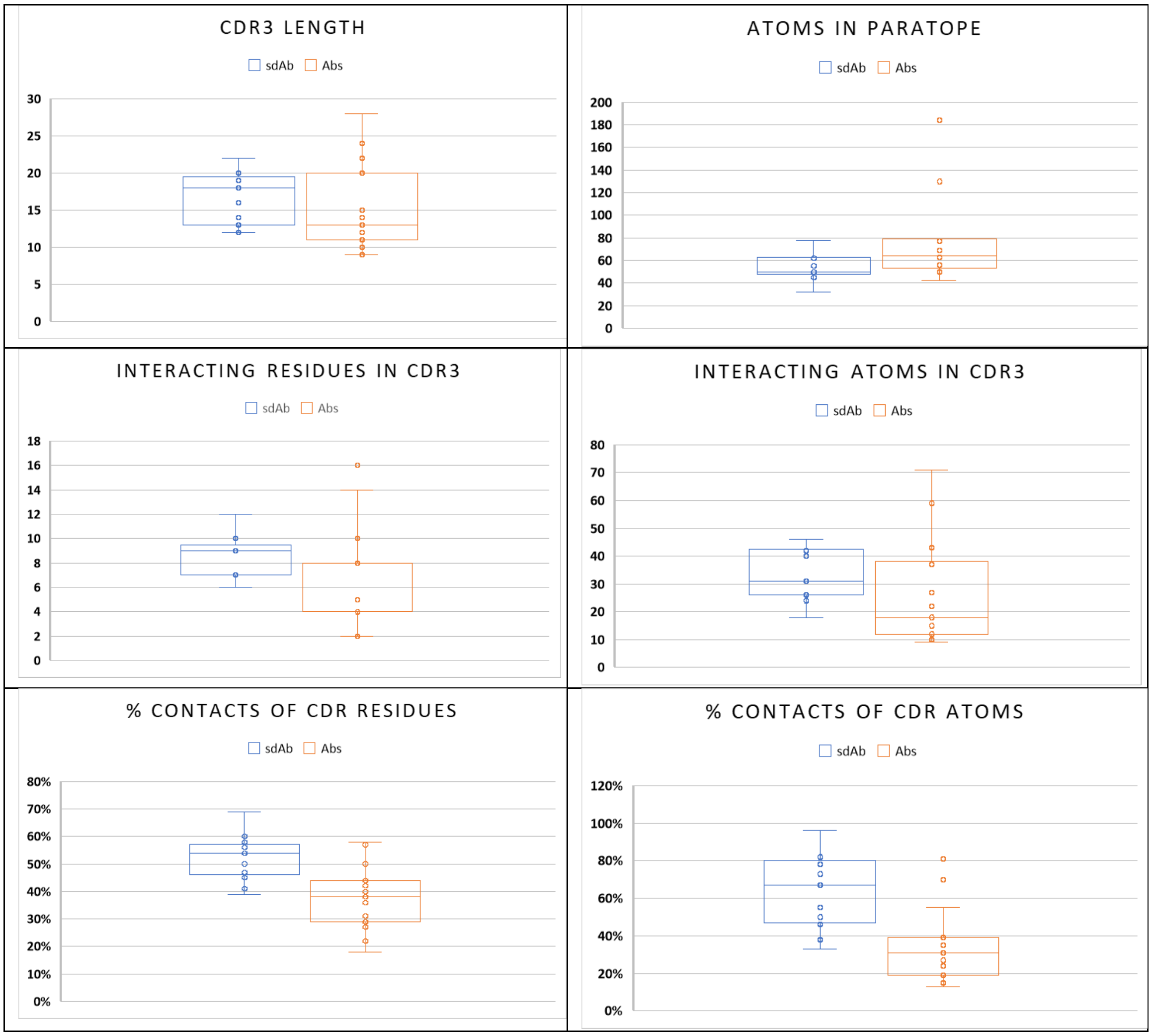

Supplementary Fig. 2. LC-MS characterization of synthesized peptides. The purity of all synthesized peptides was determined using an Agilent 1200 HPLC system using a ZORBAX Eclipse XDB-C18 column (5 µm, 4.6 x 150 mm) with a flow rate of 0.8 mL/min and a column temperature of 30°C. A linear AB gradient of 2% B/min was applied starting at 10% B to 50% B, followed by a linear AB gradient of 2.6% B/min to 90% B (solvent A: 0.1% TFA in water, solvent B: 0.05% TFA in ACN). Detection was done using a diode-array (DAD) and Mass Selective Detector (MSD). Peptide masses were calculated from the experimental mass to charge (m/z) ratios. Mass spectral peaks corresponding to the adduct are denoted as (e.g., $[M+2H]^{2+}$).

Supplementary Fig. 2

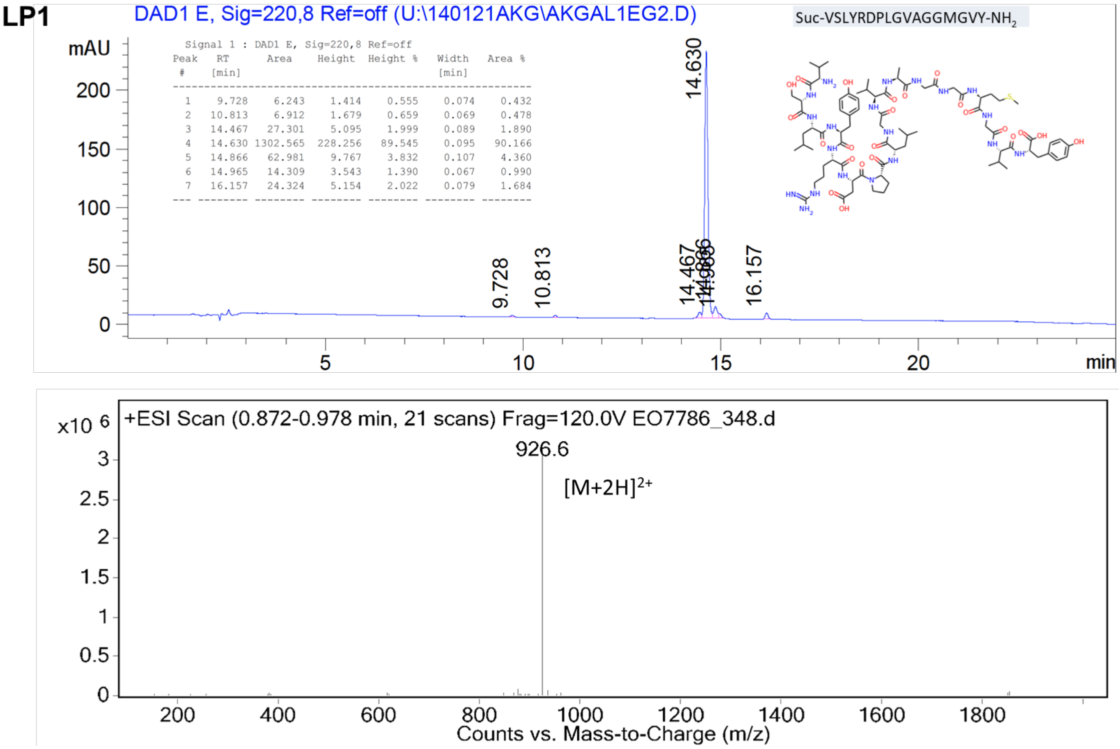

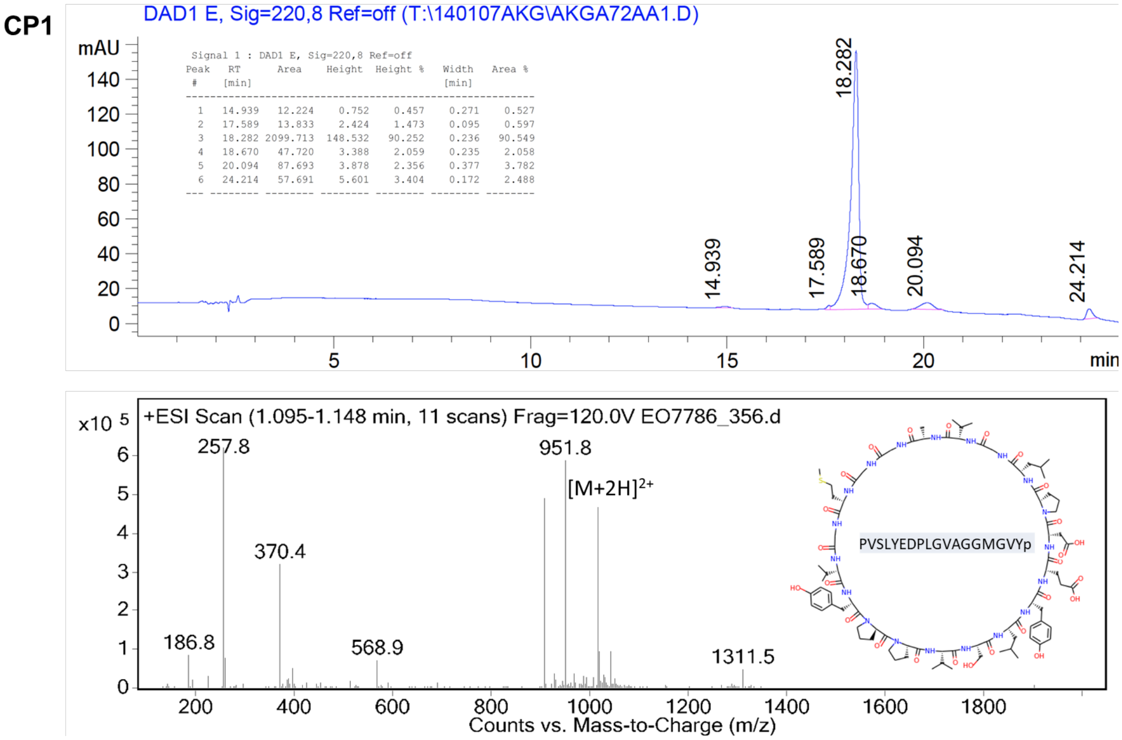

Fig. S2 (ctd.)

CP2

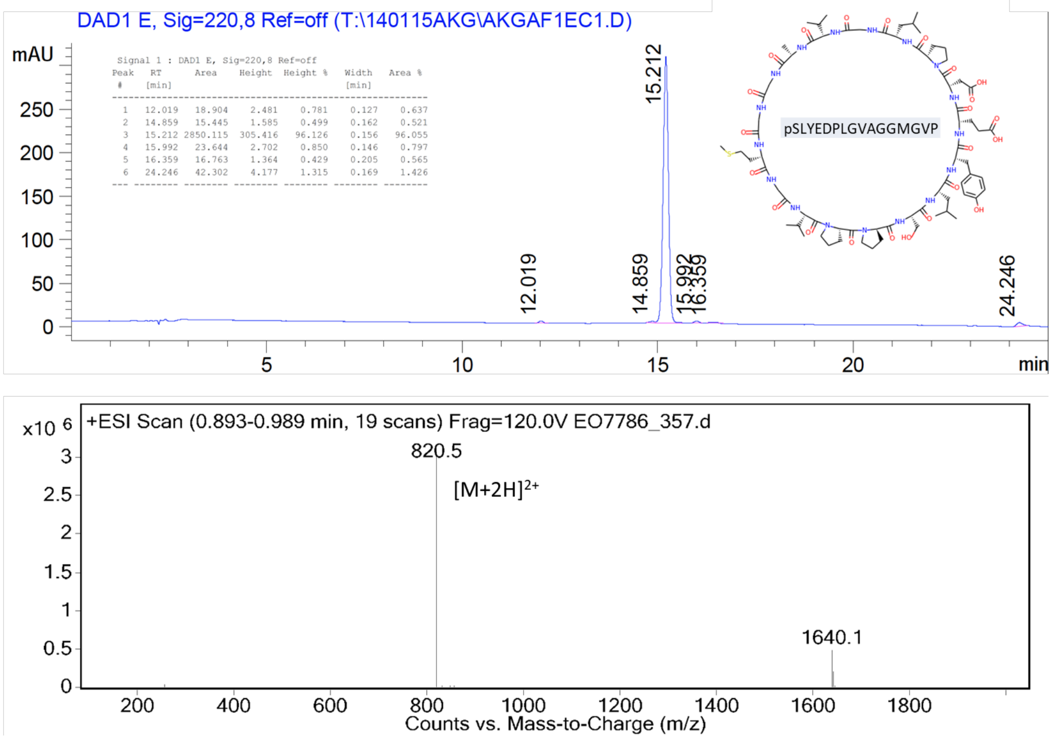

CP3

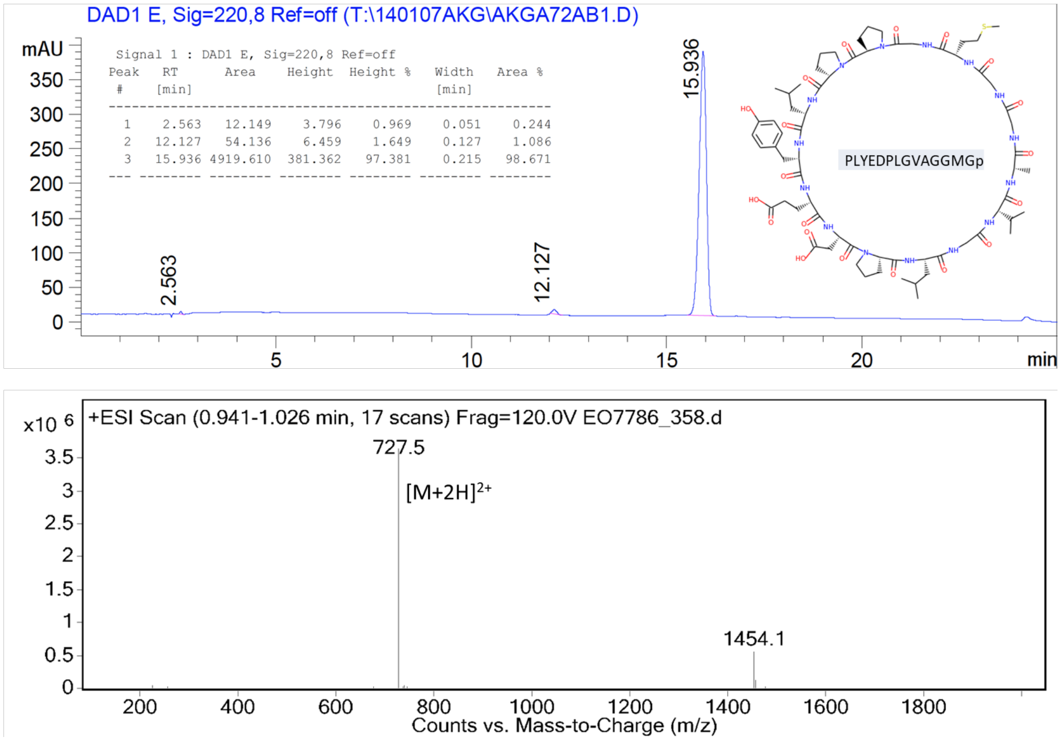

Fig. S2 (ctd.)

CP4

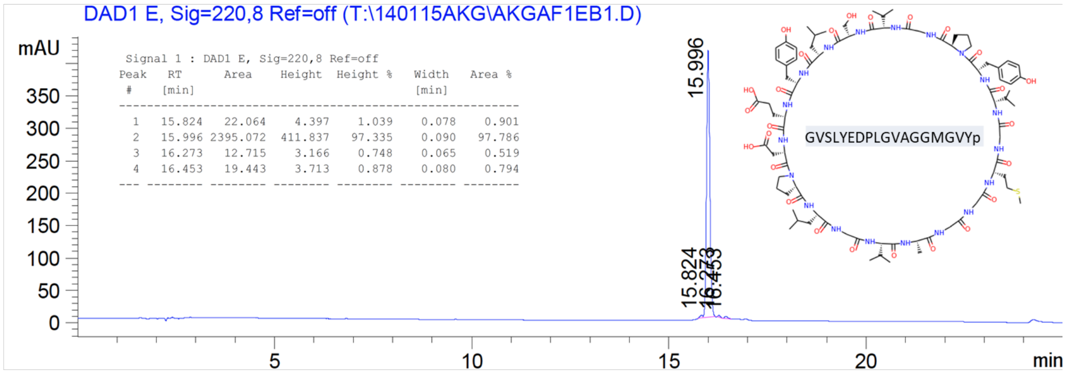

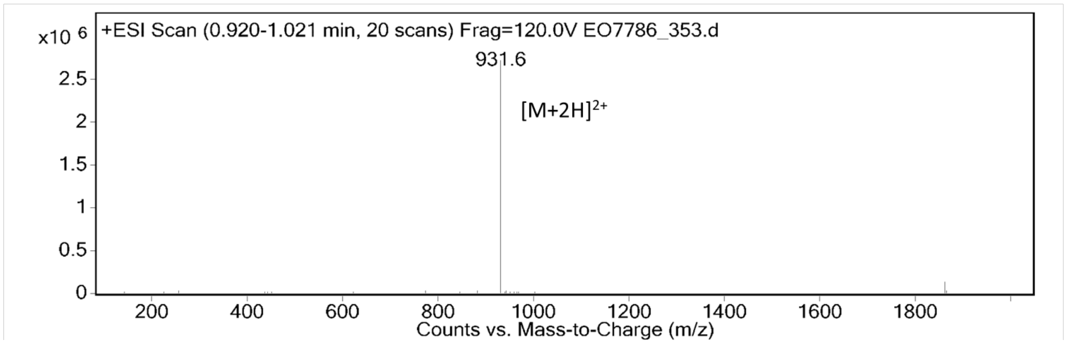

CP5

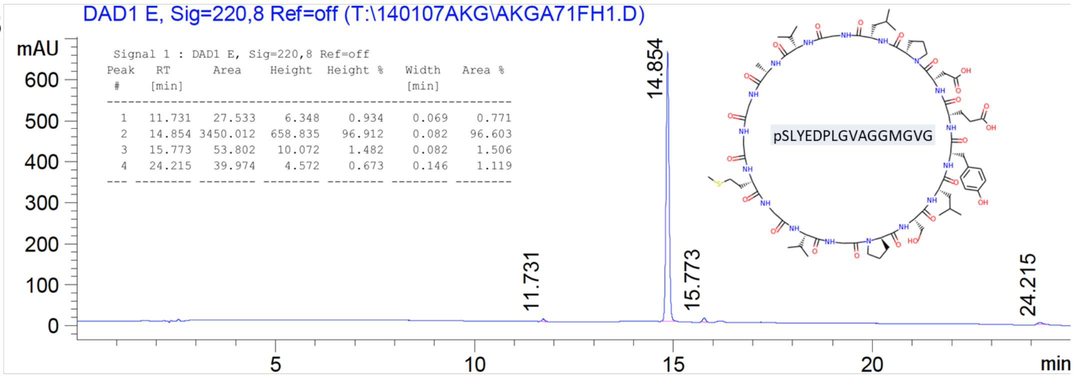

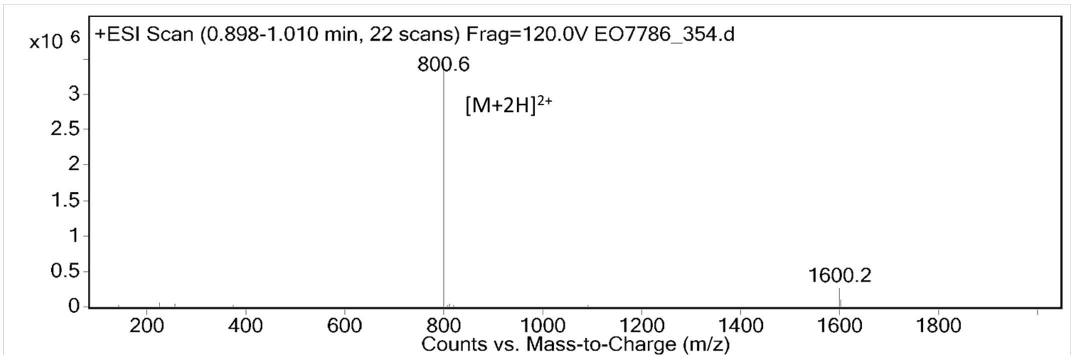

Fig. S2 (ctd.)

CP6

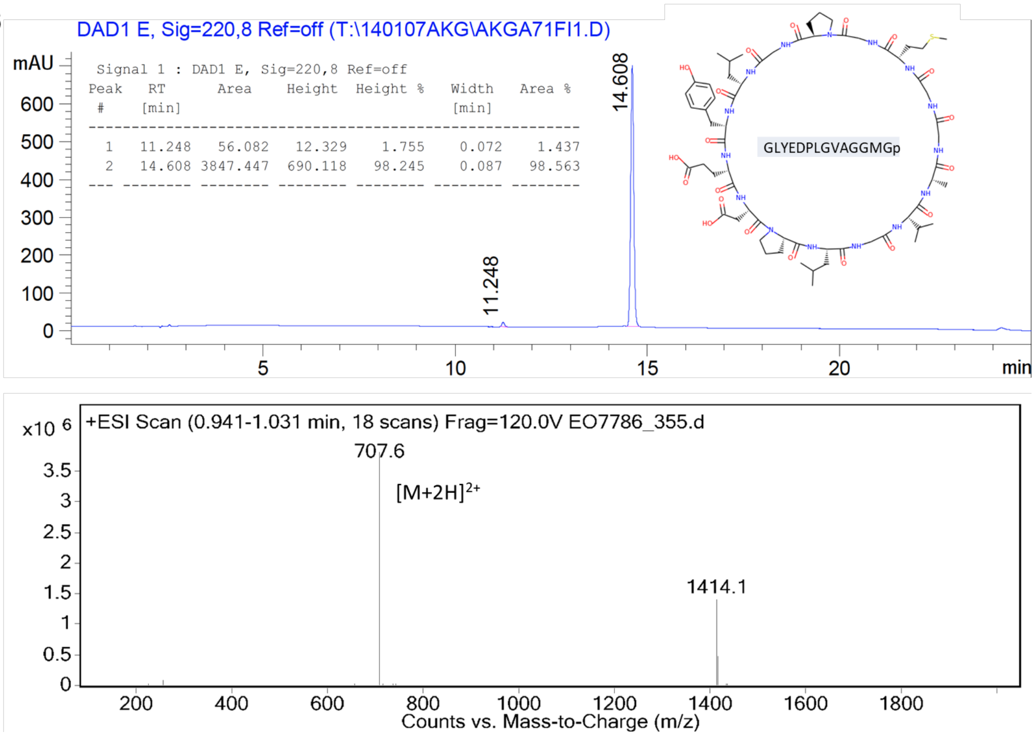

CP7

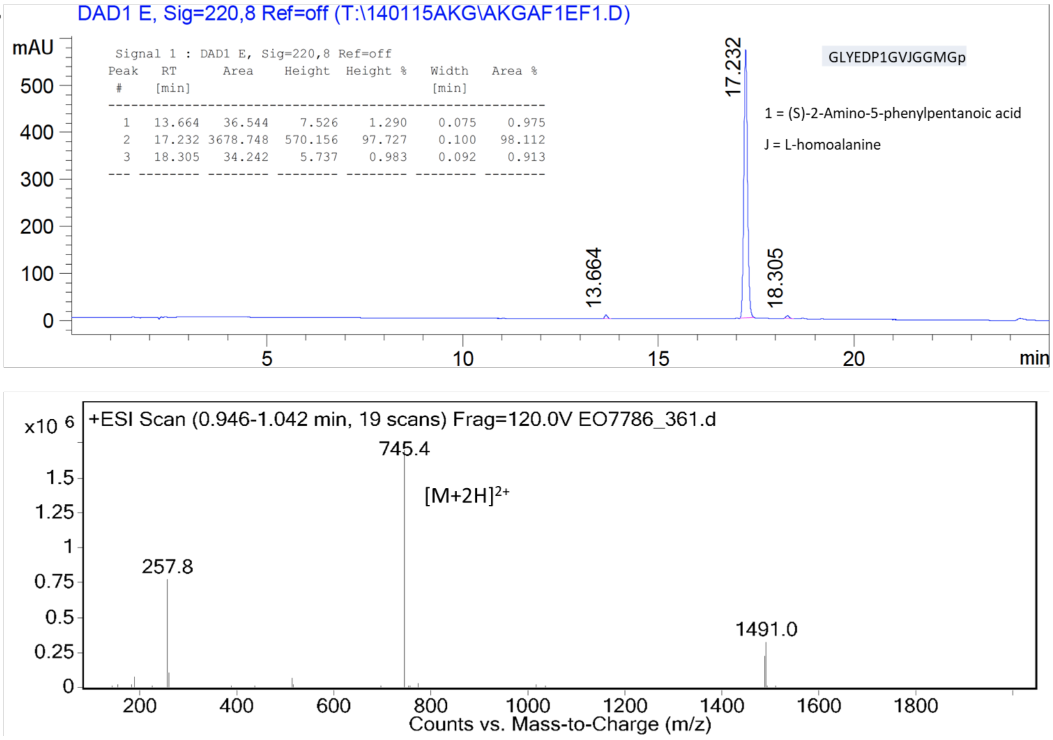

Fig. S2 (ctd.)

CP8

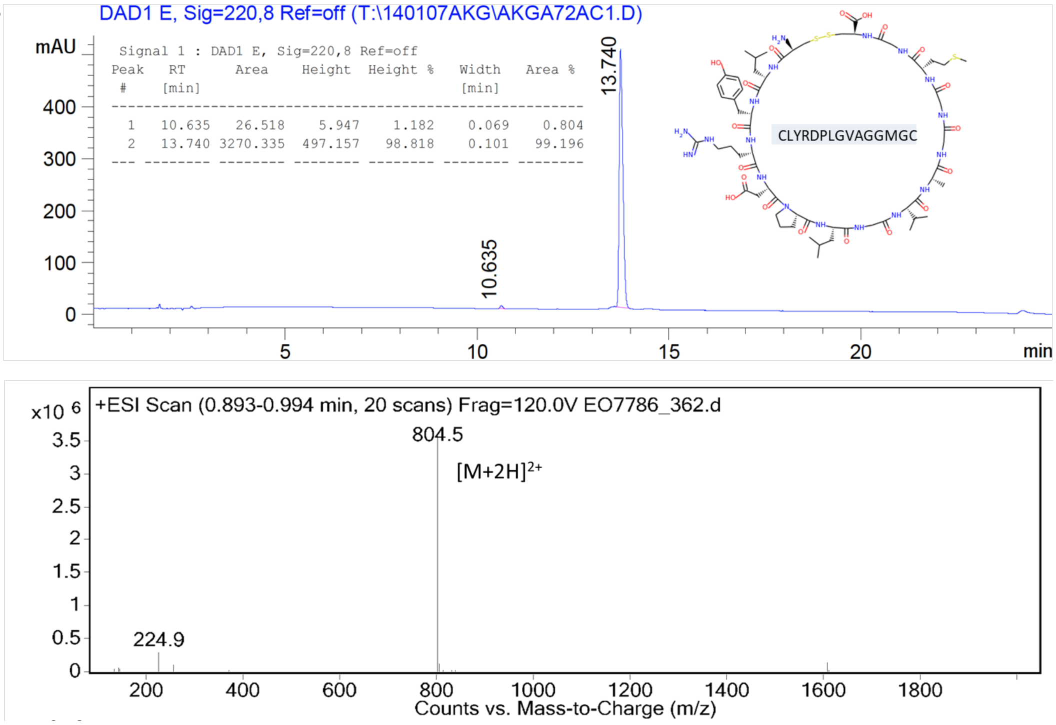

CP9

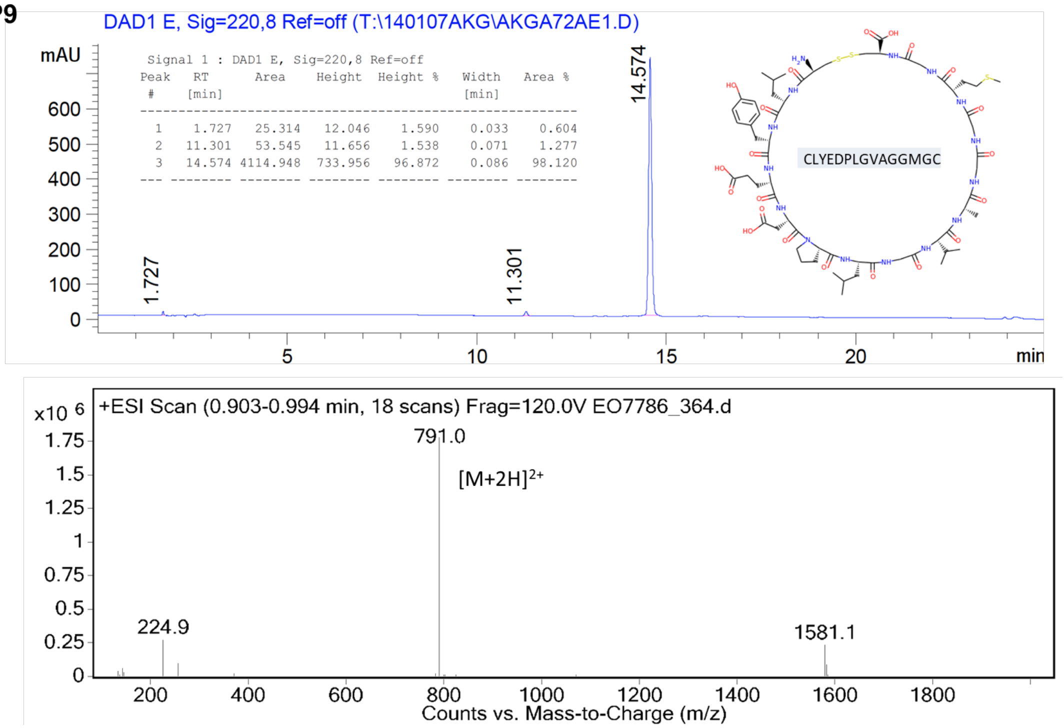

Fig. S2 (ctd.)

CP10

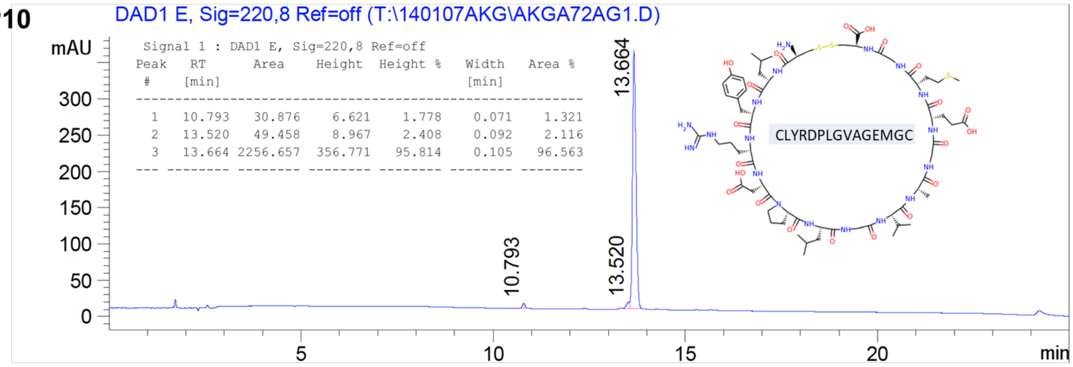

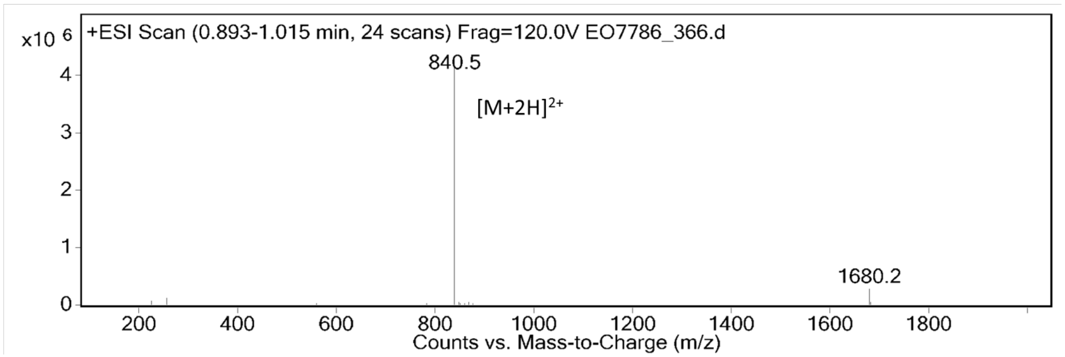

CP11

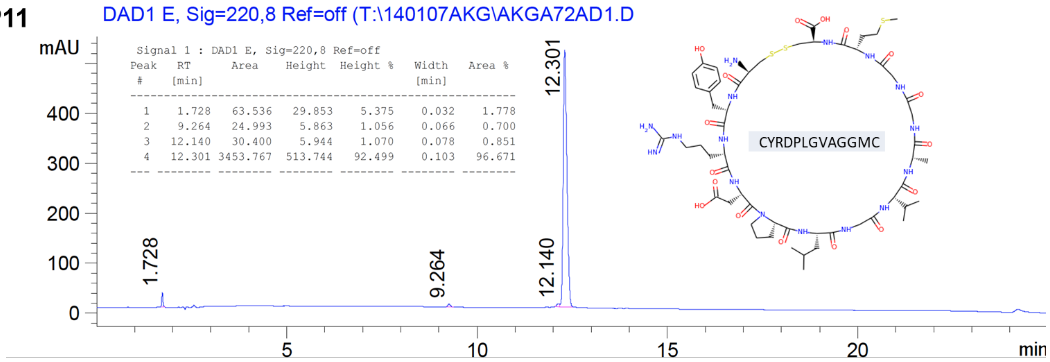

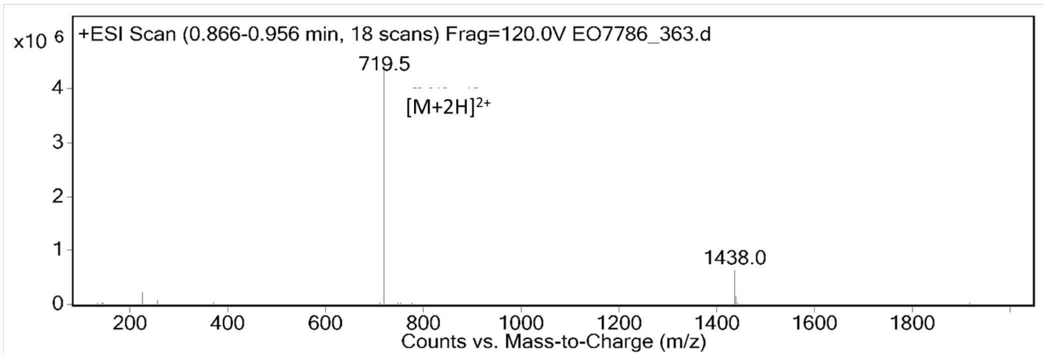

Fig. S2 (ctd.)

CP12

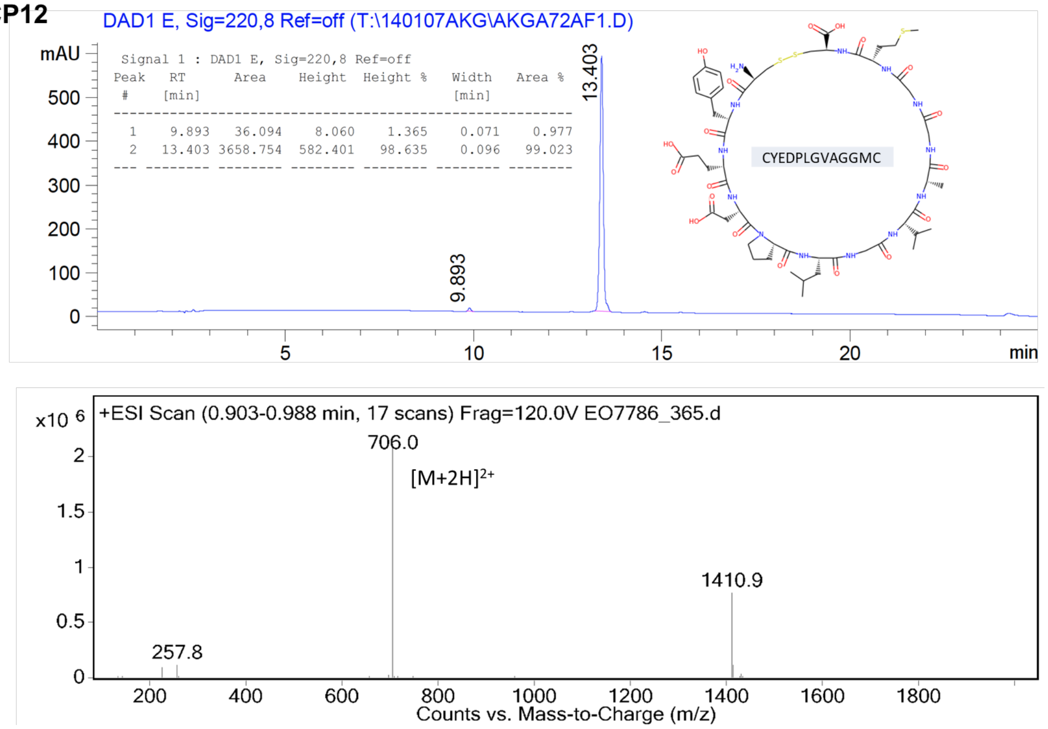

CP13

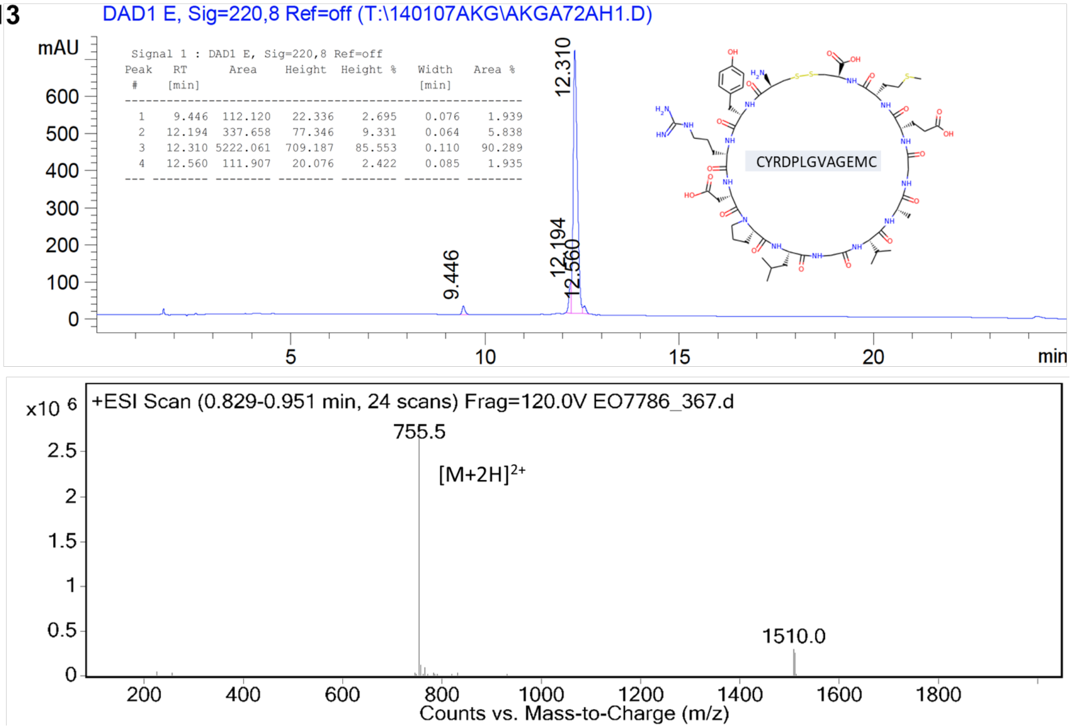

Fig. S2 (ctd.)

CP14

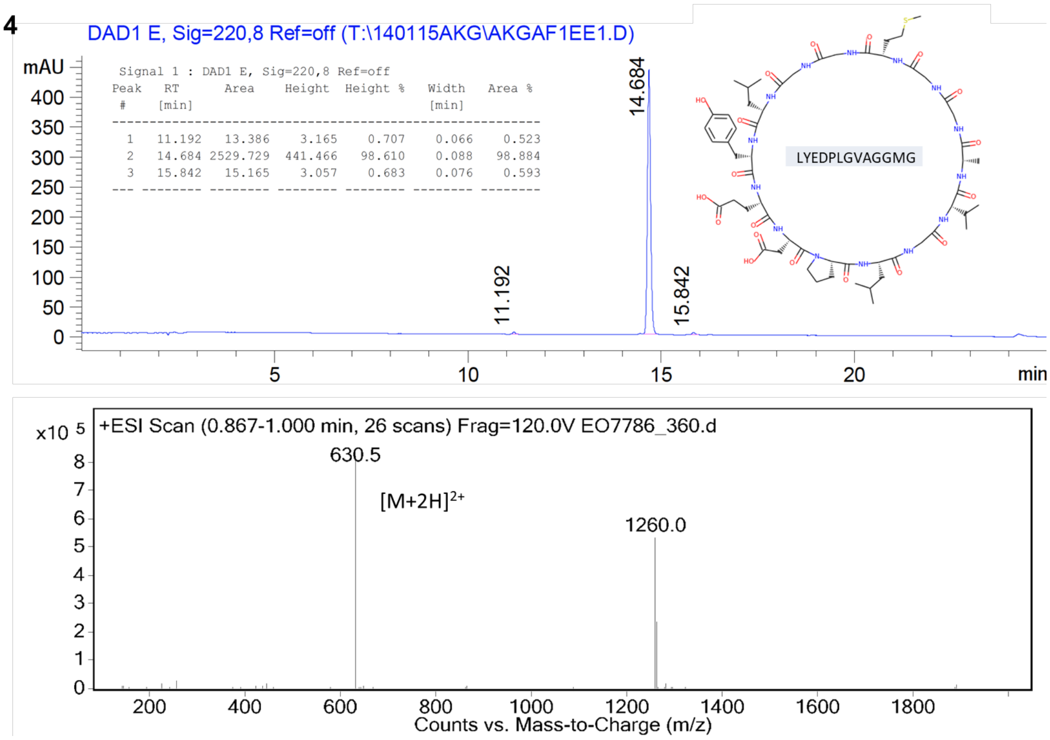

Supplementary Fig. 3. AlphaLISA competition binding assay curves for HA-peptide binding. Peptides reported in Fig. 1c were tested in AlphaLISA assay against group 1 H1 HAs (H1/Cal = A/California/07/2009 (H1N1); H1/NCa = A/New Caledonia/20/1999 (H1N1)); H5 HA (H5/Viet = A/Vietnam/1203/2004 (H5N1)); group 2 H3 HA (H3/Bris = A/Brisbane/10/2007(H3N2); H7 HA (H7/Neth = A/Netherlands/219/2003 (H7N7)) and an HA from influenza B virus (B/Flo = B/Florida/4/2006). The competition experiments were performed using a small protein HB80.4 that was designed to bind to the HA stem[1] and Fab 2D1 of an HA head binding antibody[2] as controls. Experiments were performed in duplicate to ensure reproducibility; the curves of one experiment are shown.

Supplementary Fig. 3

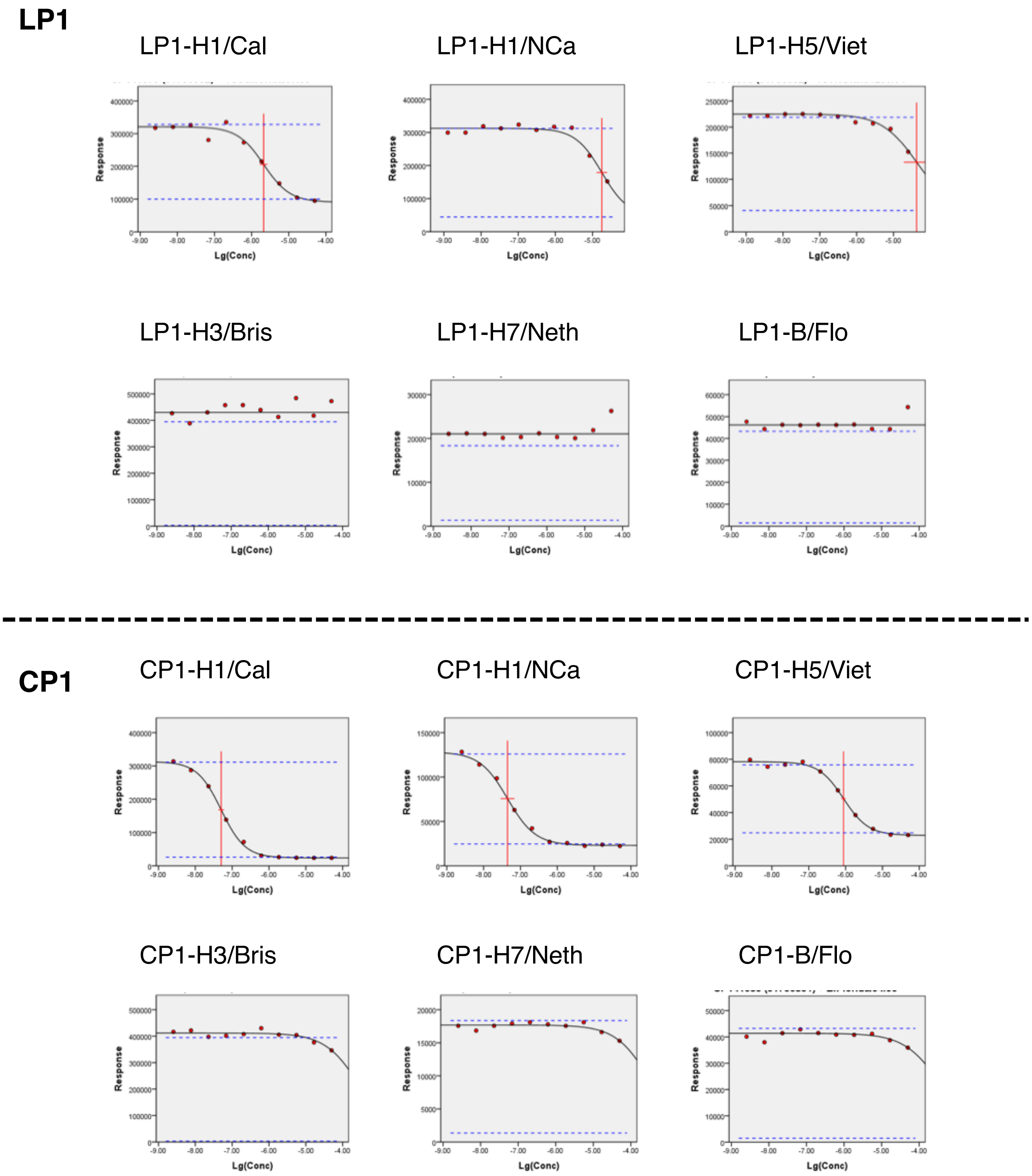

Fig. S3 (ctd.)

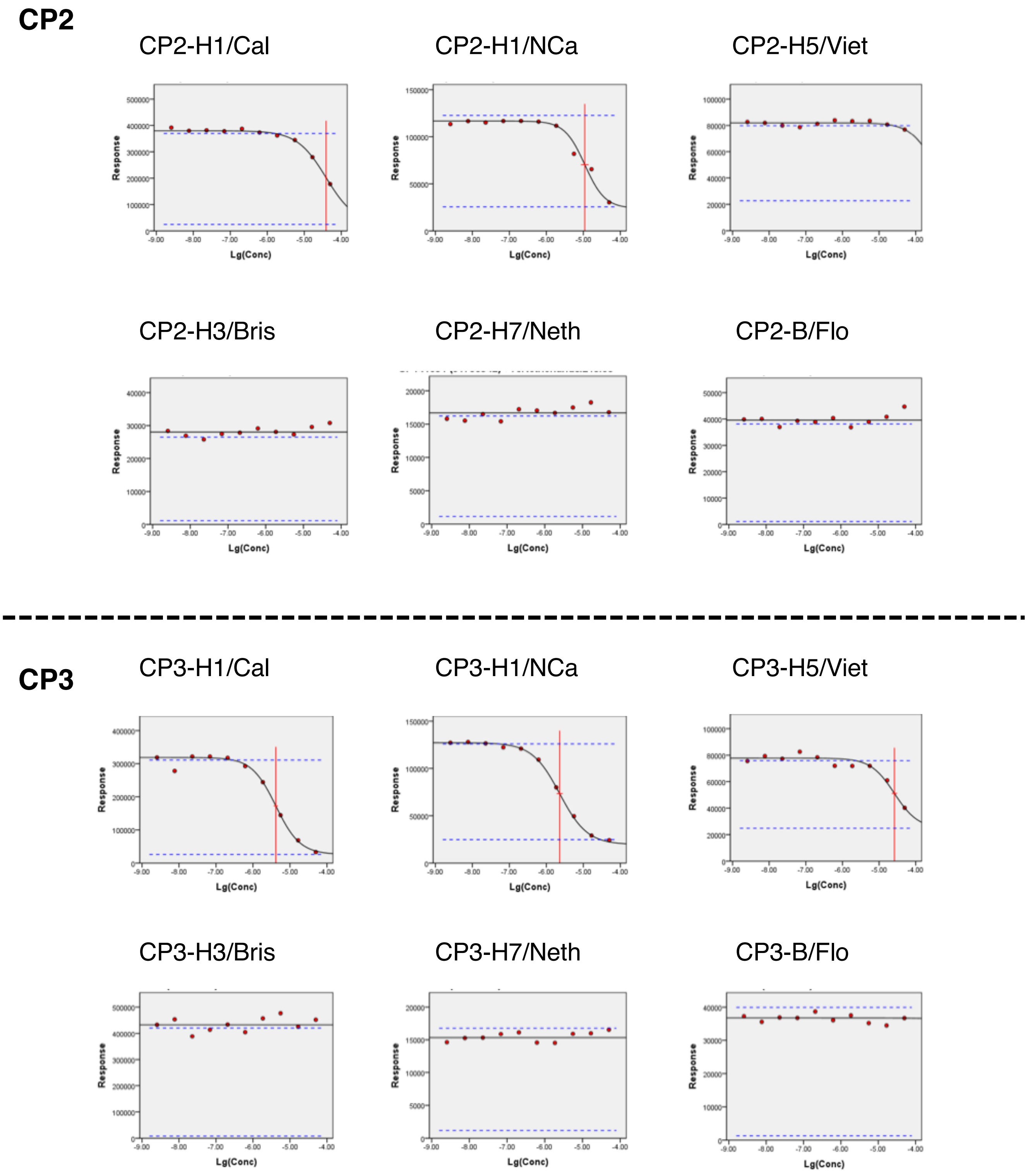
CP2
CP2-H1/Cal
CP2-H1/NCa
CP2-H5/Viet
CP2-H3/Bris
CP2-H7/Neth
CP2-B/Flo
CP3
CP3-H1/Cal
CP3-H1/NCa
CP3-H5/Viet
CP3-H3/Bris
CP3-H7/Neth
CP3-B/Flo
Response
Lg(Conc)

Fig. S3 (ctd.)

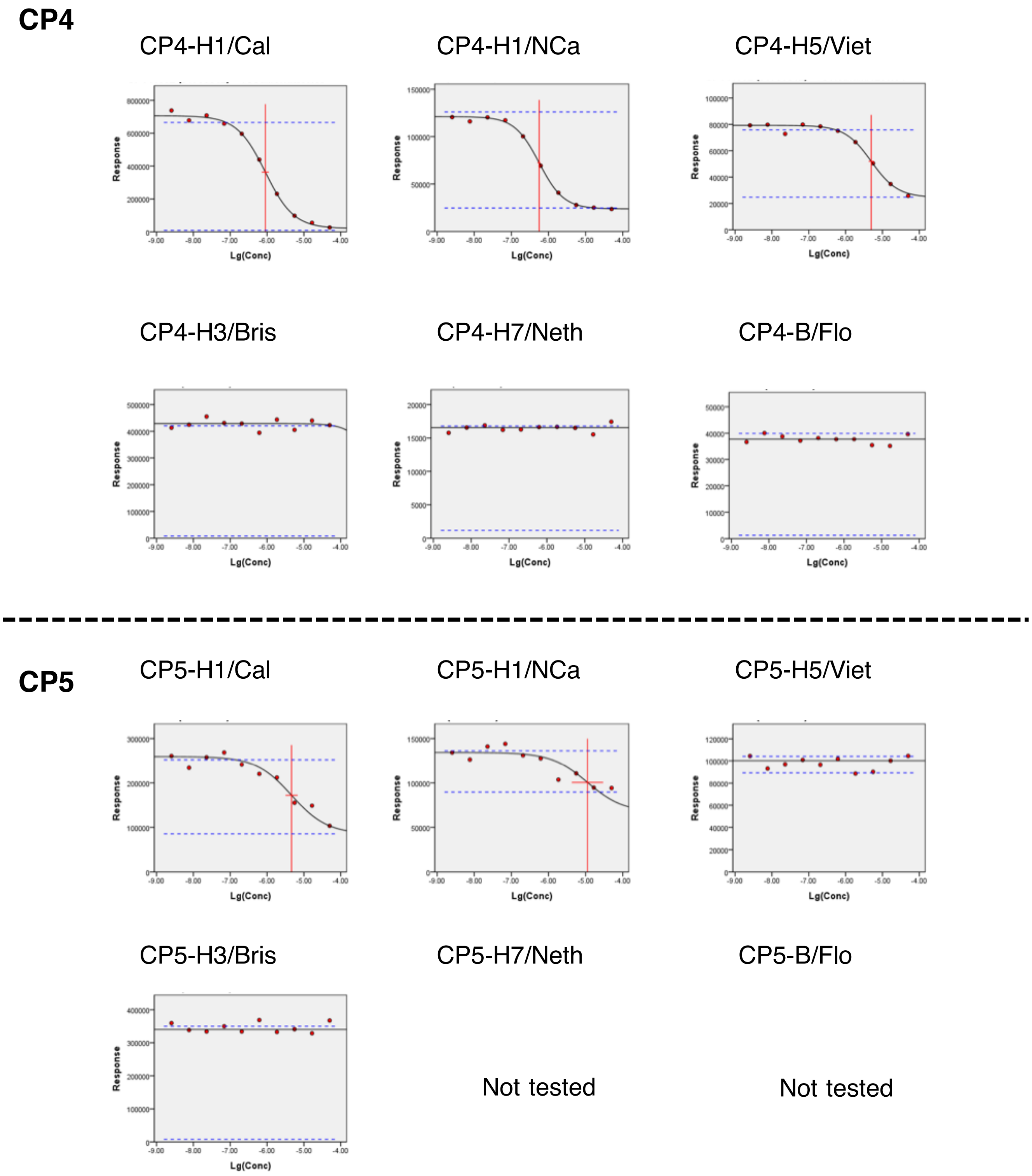
CP4
CP4-H1/Cal
CP4-H1/NCa
CP4-H5/Viet
CP4-H3/Bris
CP4-H7/Neth
CP4-B/Flo
CP5
CP5-H1/Cal
CP5-H1/NCa
CP5-H5/Viet
CP5-H3/Bris
CP5-H7/Neth
CP5-B/Flo
Not tested
Not tested
Response
Lg(Conc)

Fig. S3 (ctd.)

CP6

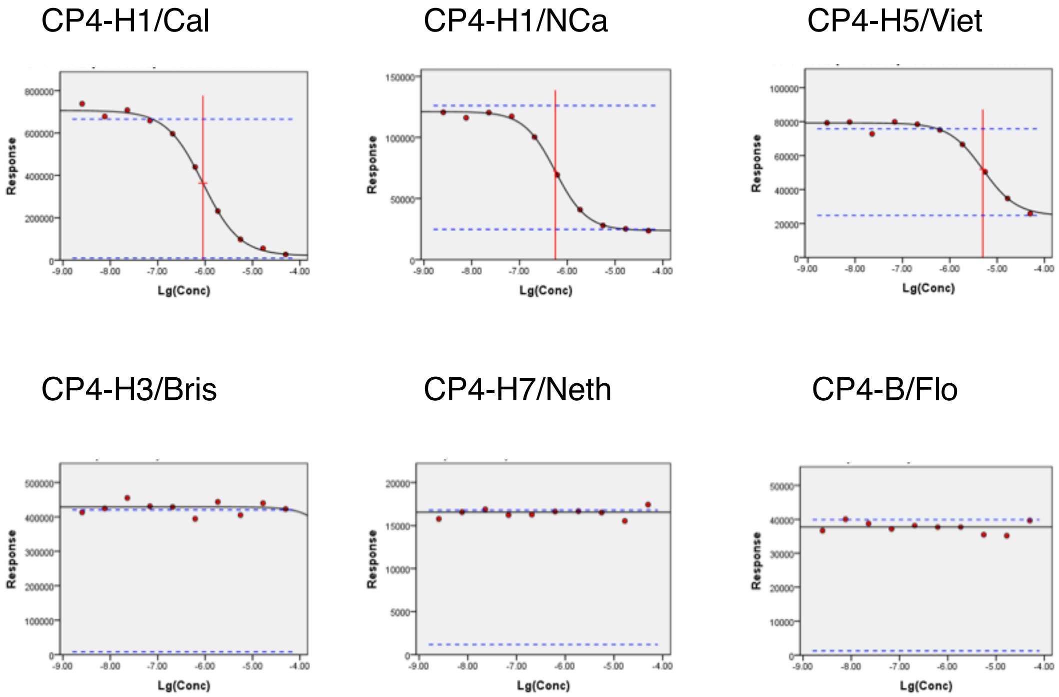

CP7

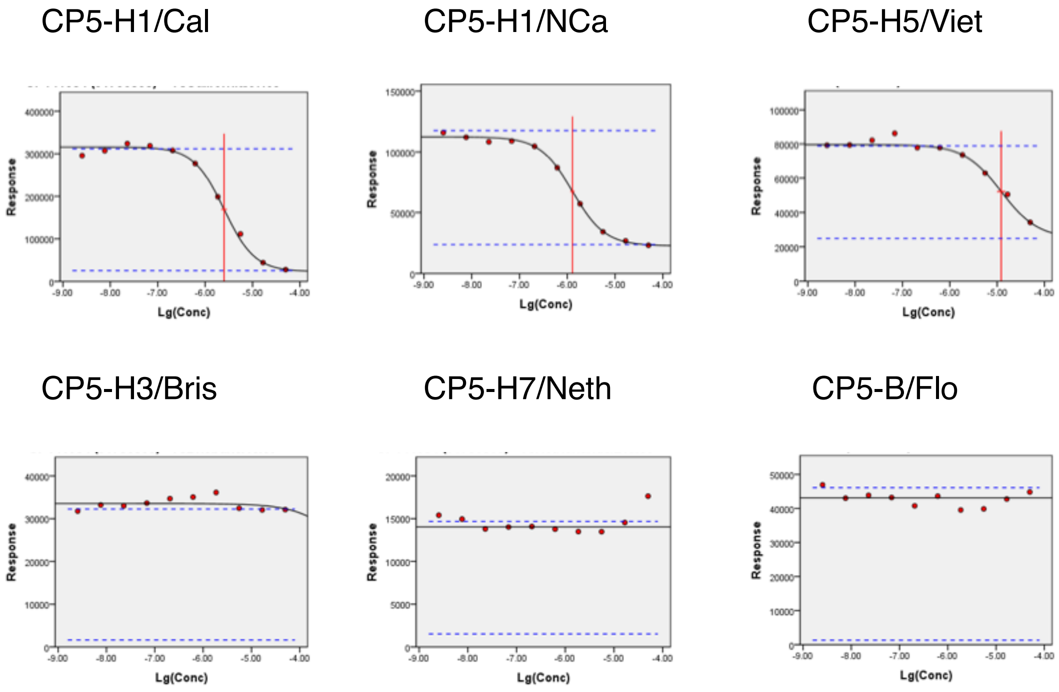

Fig. S3 (ctd.)

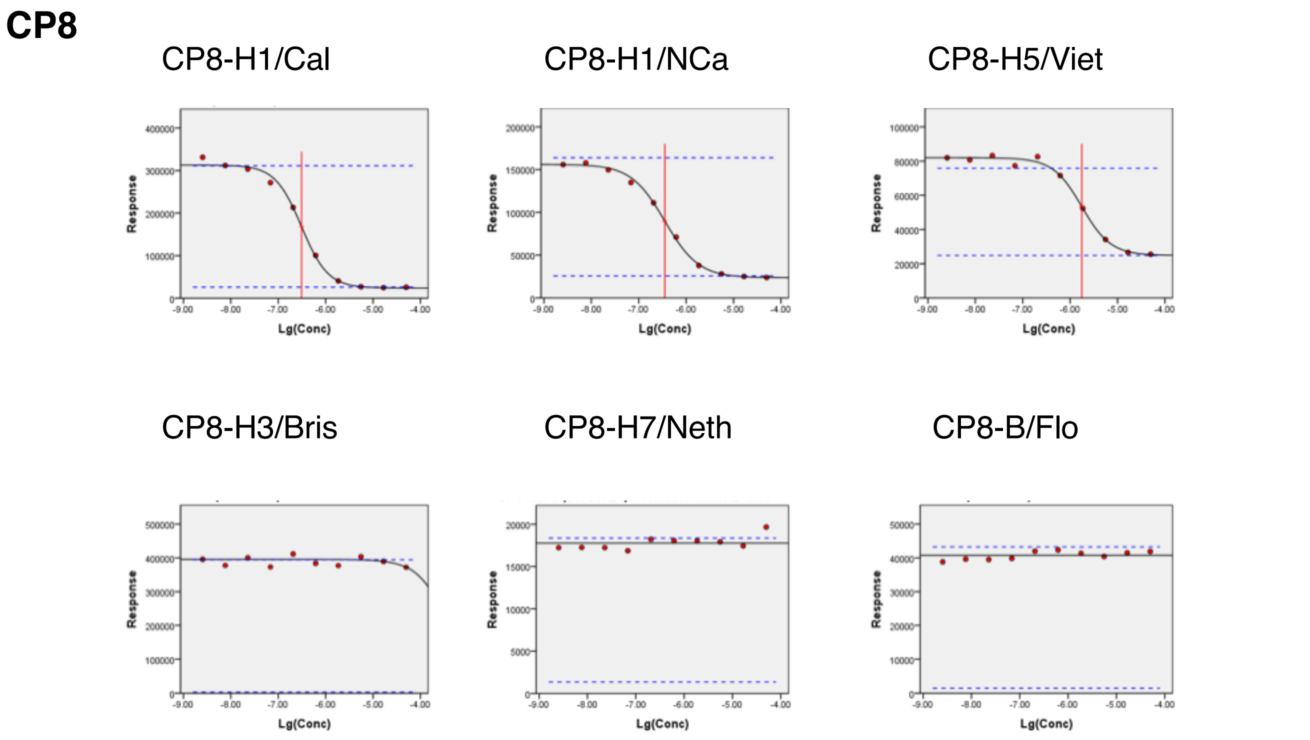
CP8
CP8-H1/Cal
CP8-H1/NCa
CP8-H5/Viet
CP8-H3/Bris
CP8-H7/Neth
CP8-B/Flo
Response
Lg(Conc)

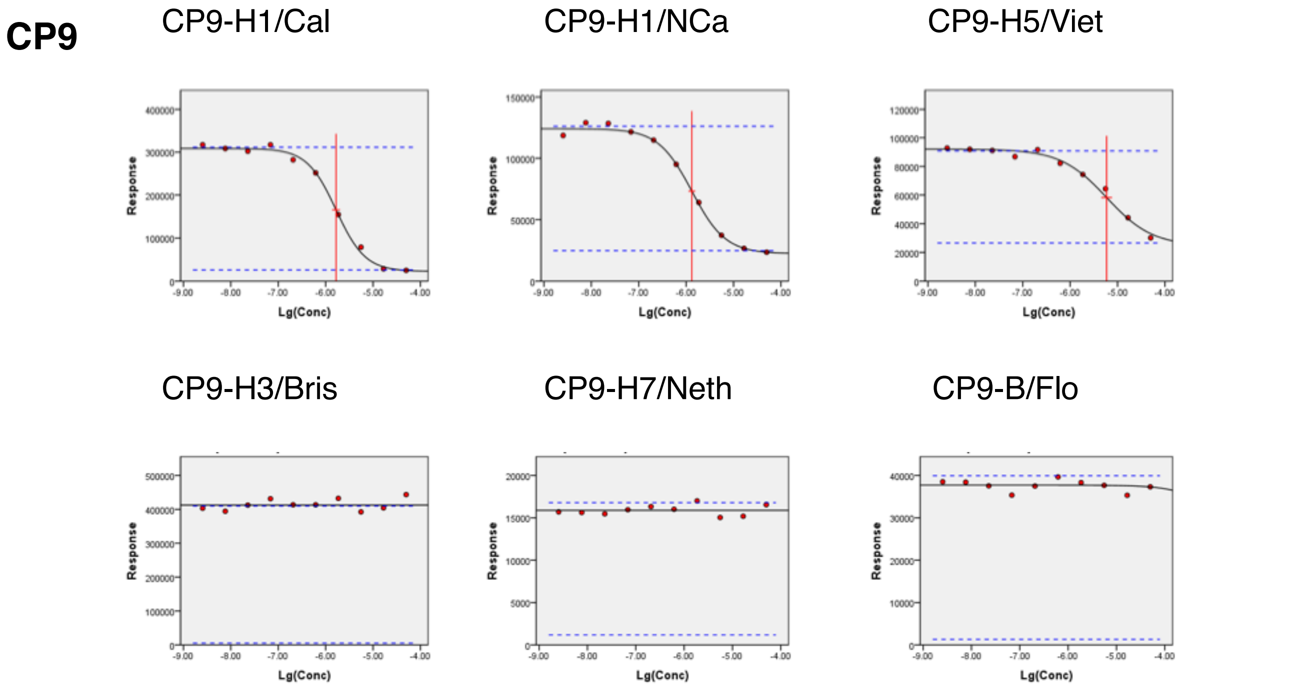
CP9
CP9-H1/Cal
CP9-H1/NCa
CP9-H5/Viet
CP9-H3/Bris
CP9-H7/Neth
CP9-B/Flo
Response
Lg(Conc)

Fig. S3 (ctd.)

CP10

CP10-H1/Cal

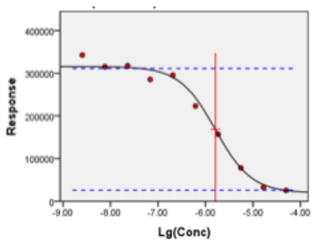

CP10-H1/NCa

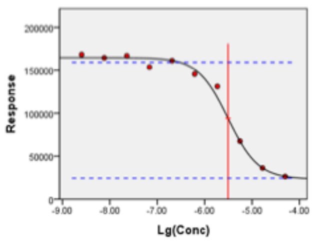

CP10-H5/Viet

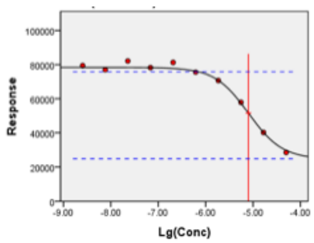

CP10-H3/Bris

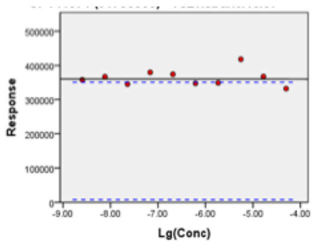

CP10-H7/Neth

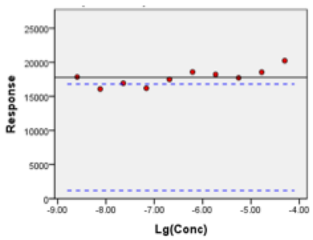

CP10-B/Flo

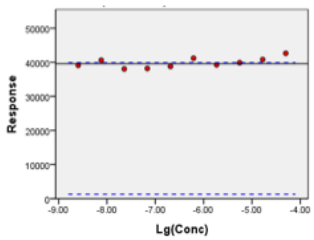

CP11

CP11-H1/Cal

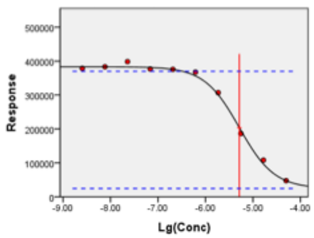

CP11-H1/NCa

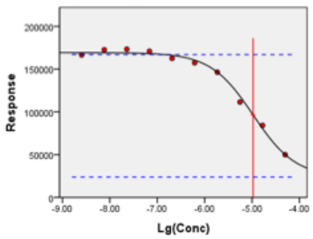

CP11-H5/Viet

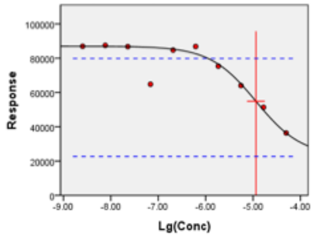

CP11-H3/Bris

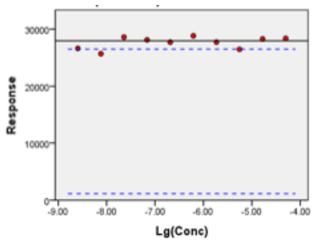

CP11-H7/Neth

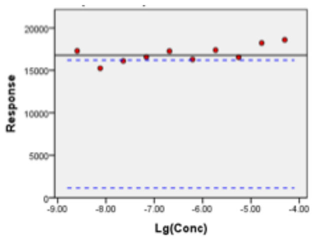

CP11-B/Flo

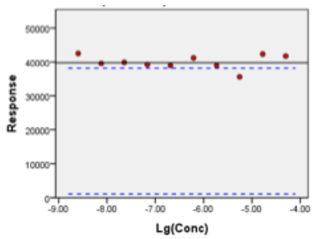

Fig. S3 (ctd.)

CP12

CP12-H1/Cal

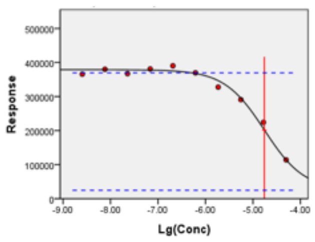

CP12-H1/NCa

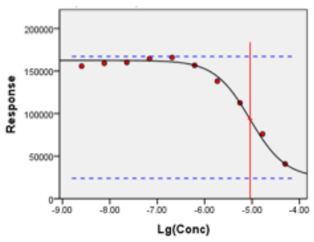

CP12-H5/Viet

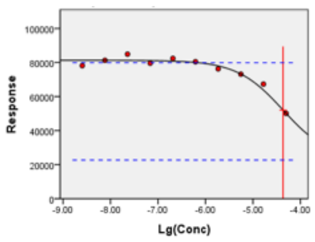

CP12-H3/Bris

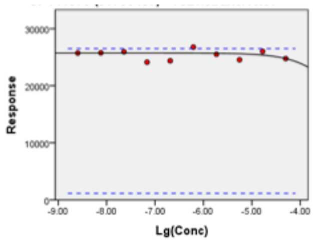

CP12-H7/Neth

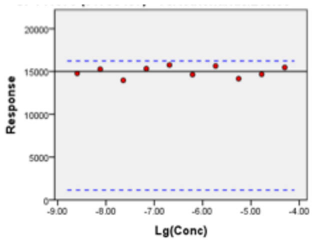

CP12-B/Flo

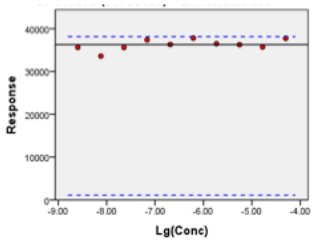

CP13

CP13-H1/Cal

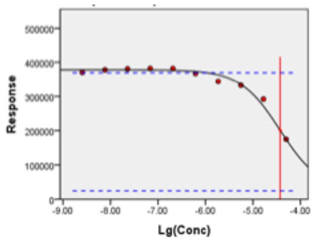

CP13-H1/NCa

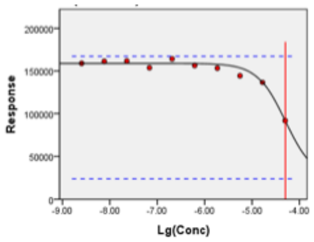

CP13-H5/Viet

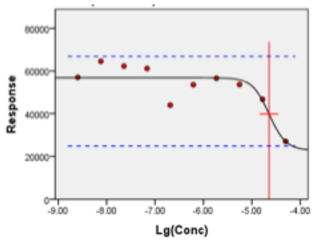

CP13-H3/Bris

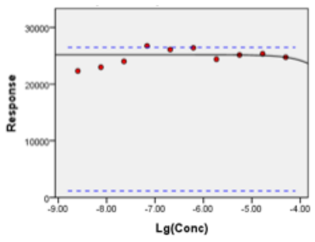

CP13-H7/Neth

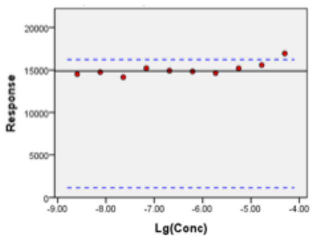

CP13-B/Flo

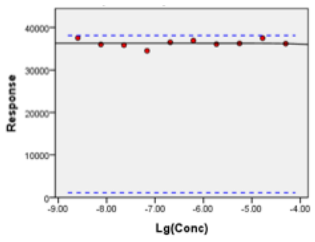

Fig. S3 (ctd.)

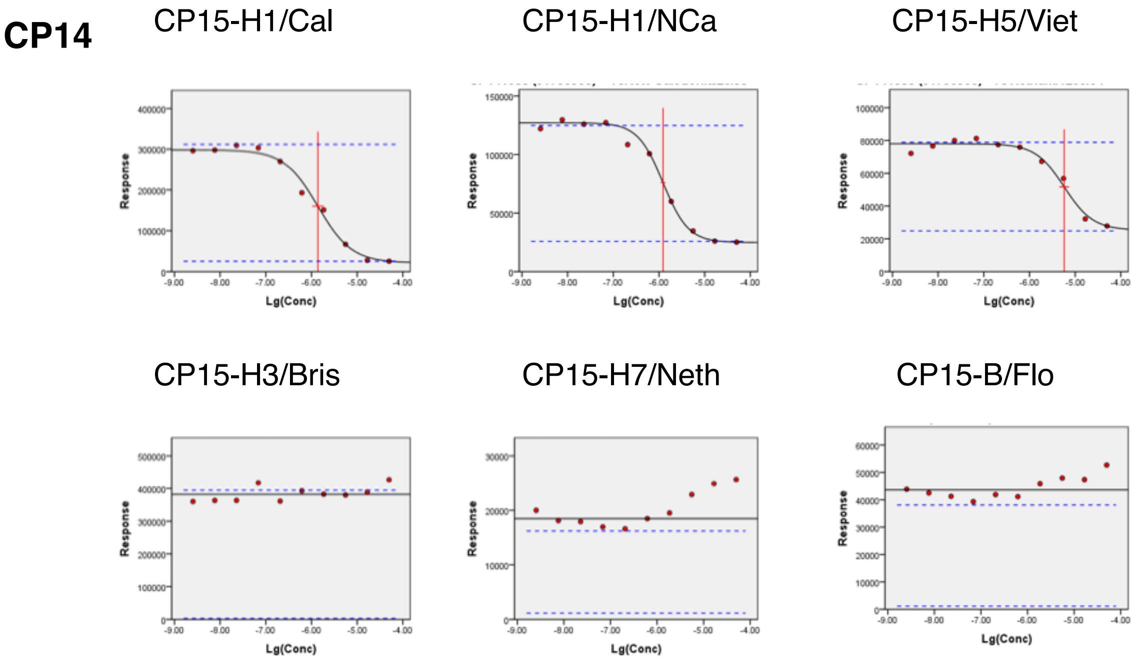
CP14
CP15-H1/Cal
CP15-H1/NCa
CP15-H5/Viet
CP15-H3/Bris
CP15-H7/Neth
CP15-B/Flo
Response
Lg(Conc)

Supplementary Fig. 4. Surface plasmon resonance (SPR) sensorgrams for HA-peptide binding. Single-cycle kinetics were run on a Biacore T200 to investigate the binding kinetics of peptides to HAs from H1 and H5 strains: H1/Cal = A/California/07/2009 (H1N1), H1/NCa = A/New Caledonia/20/1999 (H1N1), and H5/Viet = (A/Vietnam/1203/2004 (H5N1). Representative sensorgrams in response units (RU) plotted against time of injection are shown. Curves are the experimental trace (yellow lines) obtained from SPR experiments and the overlaid black lines are the best global fits (1:1 Langmuir binding model) to the data used to calculate the association rate constants (K_a) and dissociation rate constants (K_d). Experiments were performed in duplicate to ensure reproducibility; the results of one experiment are shown. Plots for each peptide are represented as follows: first plot- SPR sensorgram; second plot- SPR derived steady state kinetic dose-response curve; third plot- k_{off} response over time and fourth plot- k_{on} response over time.

Supplementary Fig. 4

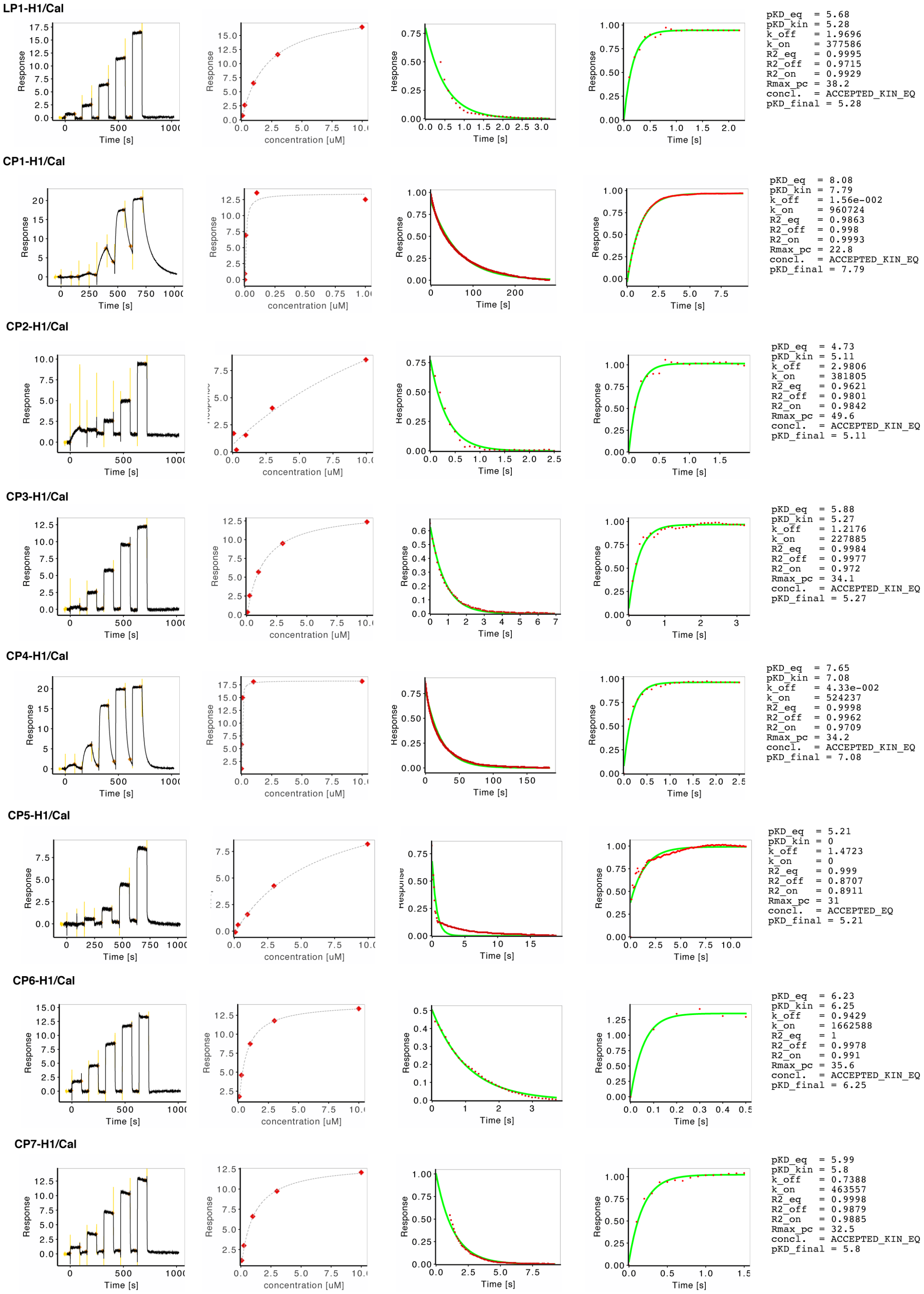

Fig. S4 (ctd.)

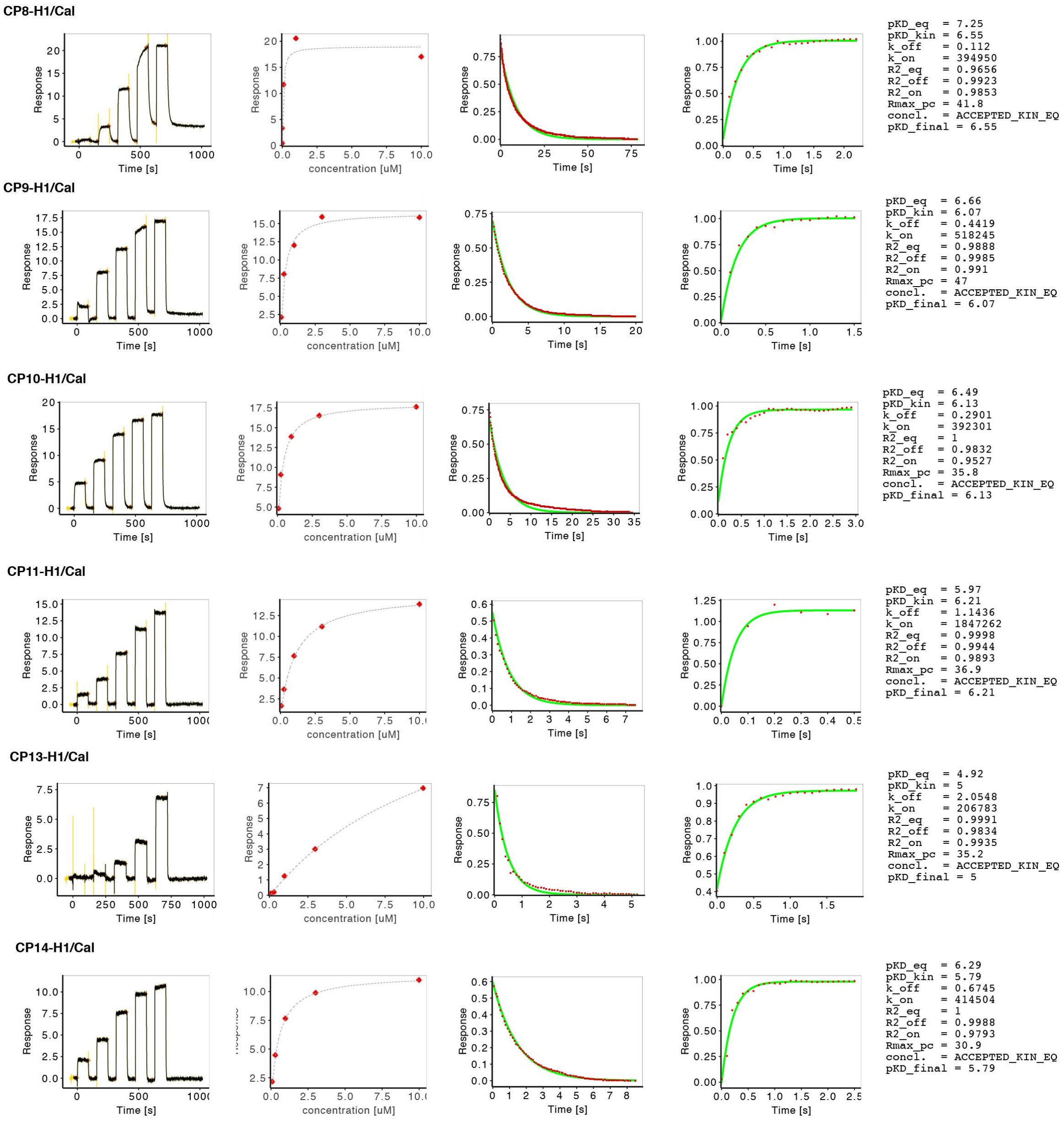
CP8-H1/Cal
pKD_eq = 7.25
pKD_kin = 6.55
k_off = 0.112
k_on = 394950
R2_eq = 0.9656
R2_off = 0.9923
R2_on = 0.9853
Rmax_pc = 41.8
concl. = ACCEPTED_KIN_EQ
pKD_final = 6.55
CP9-H1/Cal
pKD_eq = 6.66
pKD_kin = 6.07
k_off = 0.4419
k_on = 518245
R2_eq = 0.9888
R2_off = 0.9985
R2_on = 0.991
Rmax_pc = 47
concl. = ACCEPTED_KIN_EQ
pKD_final = 6.07
CP10-H1/Cal
pKD_eq = 6.49
pKD_kin = 6.13
k_off = 0.2901
k_on = 392301
R2_eq = 1
R2_off = 0.9832
R2_on = 0.9527
Rmax_pc = 35.8
concl. = ACCEPTED_KIN_EQ
pKD_final = 6.13
CP11-H1/Cal
pKD_eq = 5.97
pKD_kin = 6.21
k_off = 1.1436
k_on = 1847262
R2_eq = 0.9998
R2_off = 0.9944
R2_on = 0.9893
Rmax_pc = 36.9
concl. = ACCEPTED_KIN_EQ
pKD_final = 6.21
CP13-H1/Cal
pKD_eq = 4.92
pKD_kin = 5
k_off = 2.0548
k_on = 206783
R2_eq = 0.9991
R2_off = 0.9834
R2_on = 0.9935
Rmax_pc = 35.2
concl. = ACCEPTED_KIN_EQ
pKD_final = 5
CP14-H1/Cal
pKD_eq = 6.29
pKD_kin = 5.79
k_off = 0.6745
k_on = 414504
R2_eq = 1
R2_off = 0.9988
R2_on = 0.9793
Rmax_pc = 30.9
concl. = ACCEPTED_KIN_EQ
pKD_final = 5.79
Response
Time [s]
concentration [uM]

Fig. S4 (ctd.)

LP1-H1/NCa

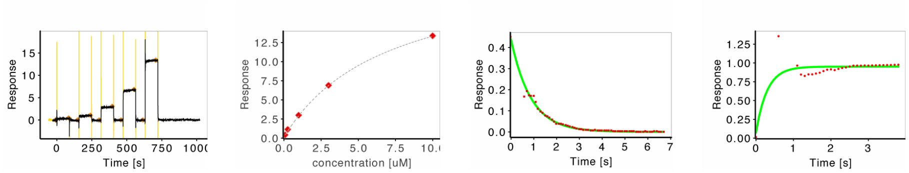


```
pKD_eq  = 5.16
pKD_kin = 5.29
k_off   = 1.1488
k_on    = 224587
R2_eq   = 0.9999
R2_off  = 0.9896
R2_on   = 0.6831
Rmax_pc = 33
concl.  = ACCEPTED_EQ
pKD_final = 5.16
```

CP1-H1/NCa

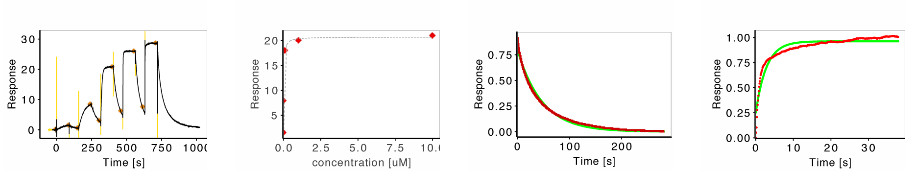


```
pKD_eq  = 7.77
pKD_kin = 6.19
k_off   = 2.12e-002
k_on    = 33212
R2_eq   = 0.999
R2_off  = 0.9945
R2_on   = 0.8939
Rmax_pc = 29.2
concl.  = ACCEPTED_EQ
pKD_final = 7.77
```

CP2-H1/NCa

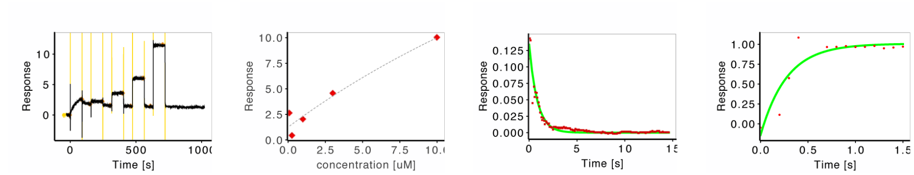


```
pKD_eq  = 4.38
pKD_kin = 5.31
k_off   = 1.1853
k_on    = 243446
R2_eq   = 0.9441
R2_off  = 0.9401
R2_on   = 0.8388
Rmax_pc = 76.3
concl.  = REJECTED
pKD_final = 0
```

CP3-H1/NCa

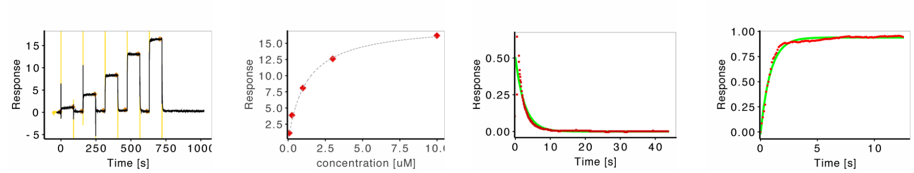


```
pKD_eq  = 5.91
pKD_kin = 5.32
k_off   = 0.3756
k_on    = 77821
R2_eq   = 0.9985
R2_off  = 0.8529
R2_on   = 0.9851
Rmax_pc = 34.4
concl.  = ACCEPTED_EQ
pKD_final = 5.91
```

CP4-H1/NCa

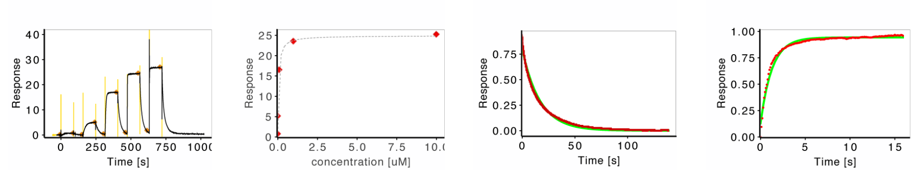


```
pKD_eq  = 7.29
pKD_kin = 6.09
k_off   = 5.92e-002
k_on    = 72035
R2_eq   = 0.9988
R2_off  = 0.9941
R2_on   = 0.9782
Rmax_pc = 35.9
concl.  = ACCEPTED_KIN_EQ
pKD_final = 6.09
```

CP5-H1/NCa

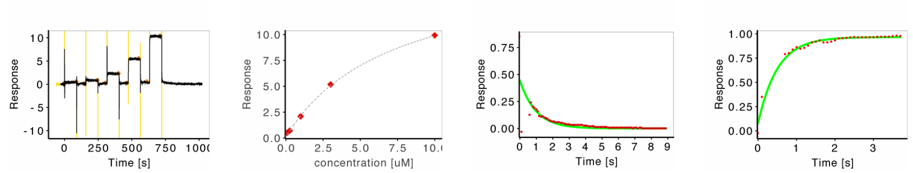


```
pKD_eq  = 5.14
pKD_kin = 4.97
k_off   = 1.0298
k_on    = 96292
R2_eq   = 0.9992
R2_off  = 0.6401
R2_on   = 0.9756
Rmax_pc = 29.6
concl.  = ACCEPTED_EQ
pKD_final = 5.14
```

CP6-H1/NCa

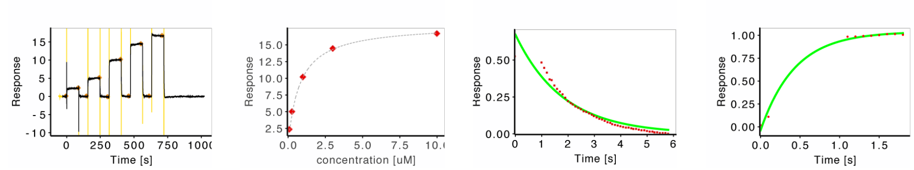


```
pKD_eq  = 6.1
pKD_kin = 5.5
k_off   = 0.5536
k_on    = 175841
R2_eq   = 0.9998
R2_off  = 0.9664
R2_on   = 0.9927
Rmax_pc = 33.6
concl.  = ACCEPTED_EQ
pKD_final = 6.1
```

CP7-H1/NCa

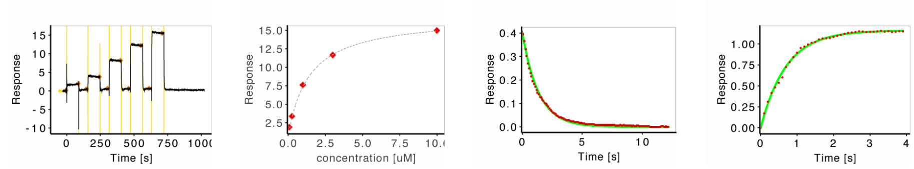


```
pKD_eq  = 5.85
pKD_kin = 4.97
k_off   = 0.7397
k_on    = 69185
R2_eq   = 0.9993
R2_off  = 0.9973
R2_on   = 0.9965
Rmax_pc = 31.5
concl.  = ACCEPTED_KIN_EQ
pKD_final = 4.97
```

Fig. S4 (ctd.)

CP8-H1/NCa

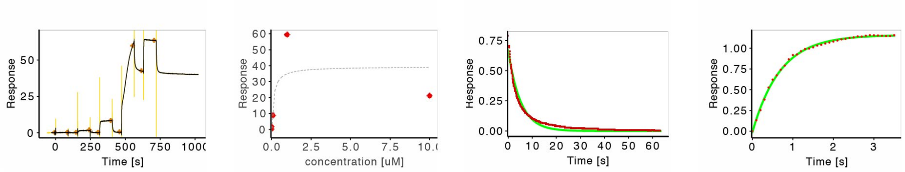


```
pKD_eq   = 6.93
pKD_kin  = 5.81
k_off    = 0.1952
k_on     = 126232
R2_eq    = 0.5879
R2_off   = 0.9783
R2_on    = 0.9979
Rmax_pc  = 72.7
concl.   = ACCEPTED_KIN
pKD_final = 5.81
```

CP9-H1/NCa

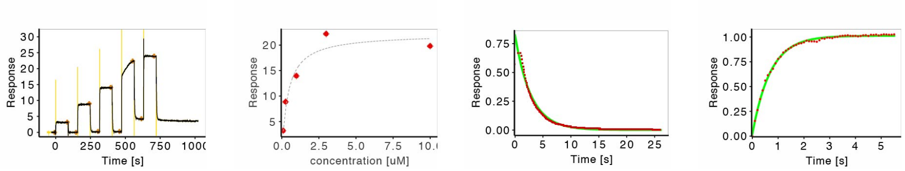


```
pKD_eq   = 6.39
pKD_kin  = 5.49
k_off    = 0.3509
k_on     = 107750
R2_eq    = 0.9481
R2_off   = 0.9797
R2_on    = 0.9975
Rmax_pc  = 42.2
concl.   = ACCEPTED_KIN
pKD_final = 5.49
```

CP10-H1/NCa

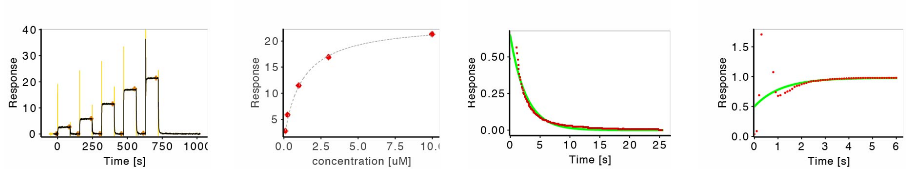


```
pKD_eq   = 5.92
pKD_kin  = 5.12
k_off    = 0.3782
k_on     = 50294
R2_eq    = 0.9994
R2_off   = 0.9682
R2_on    = 0.2834
Rmax_pc  = 36.4
concl.   = ACCEPTED_EQ
pKD_final = 5.92
```

CP11-H1/NCa

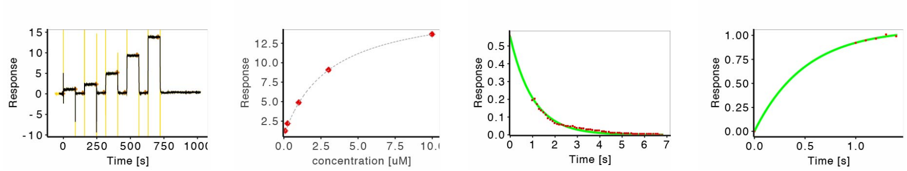


```
pKD_eq   = 5.51
pKD_kin  = 5.01
k_off    = 0.9805
k_on     = 101327
R2_eq    = 1
R2_off   = 0.9939
R2_on    = 0.9992
Rmax_pc  = 32.9
concl.   = ACCEPTED_EQ
pKD_final = 5.51
```

CP13-H1/NCa

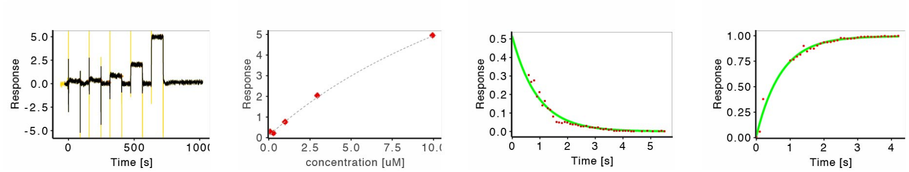


```
pKD_eq   = 4.68
pKD_kin  = 4.42
k_off    = 1.0865
k_on     = 28350
R2_eq    = 0.9985
R2_off   = 0.9764
R2_on    = 0.9855
Rmax_pc  = 27.6
concl.   = ACCEPTED_KIN_EQ
pKD_final = 4.42
```

CP14-H1/NCa

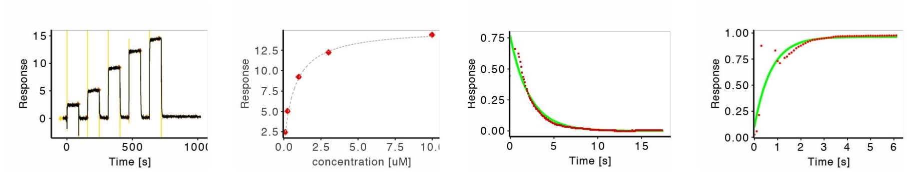


```
pKD_eq   = 6.13
pKD_kin  = 5.32
k_off    = 0.4502
k_on     = 93071
R2_eq    = 0.9993
R2_off   = 0.9497
R2_on    = 0.8582
Rmax_pc  = 30.9
concl.   = ACCEPTED_EQ
pKD_final = 6.13
```

Fig. S4 (ctd.)

LP1-H5/Viet

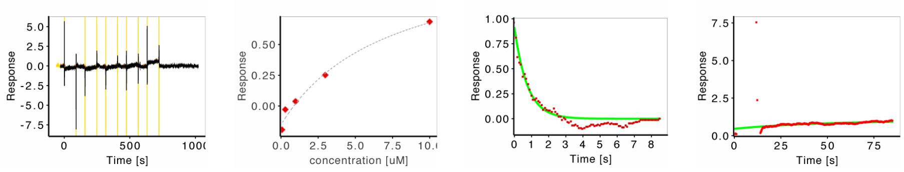


```
pKD_eq   = 5.14
pKD_kin  = 0
k_off    = 1.3328
k_on     = 0
R2_eq    = 0.9801
R2_off   = 0.938
R2_on    = 0.1143
Rmax_pc  = 1.8
concl.   = ACCEPTED_EQ
pKD_final = 5.14
```

CP1-H5/Viet

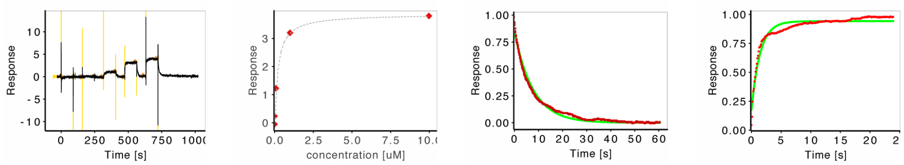


```
pKD_eq   = 6.67
pKD_kin  = 5.64
k_off    = 0.1295
k_on     = 56550
R2_eq    = 0.9991
R2_off   = 0.9905
R2_on    = 0.9337
Rmax_pc  = 6.8
concl.   = ACCEPTED_KIN_EQ
pKD_final = 5.64
```

CP2-H5/Viet

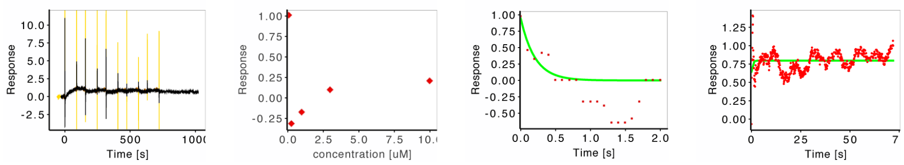


```
pKD_eq   = 0
pKD_kin  = 0
k_off    = 4.9059
k_on     = 0
R2_eq    = 1.4e-003
R2_off   = 0.3806
R2_on    = 6.3e-003
Rmax_pc  = 0
concl.   = REJECTED
pKD_final = 0
```

CP3-H5/Viet

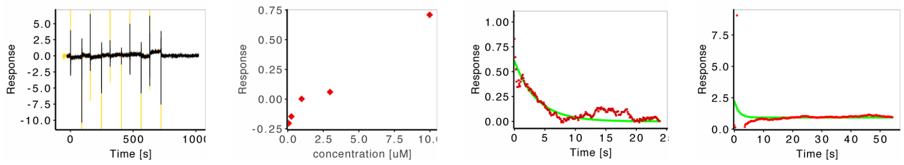


```
pKD_eq   = 0
pKD_kin  = 5.15
k_off    = 0.2701
k_on     = 38142
R2_eq    = 0.9835
R2_off   = 0.8656
R2_on    = 4.22e-002
Rmax_pc  = 0
concl.   = REJECTED
pKD_final = 0
```

CP4-H5/Viet

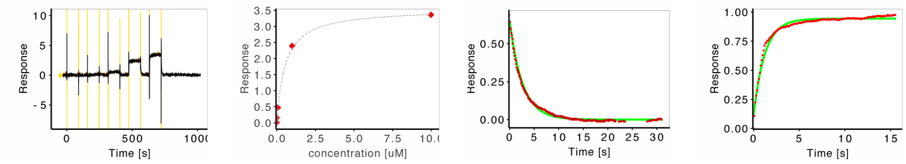


```
pKD_eq   = 6.27
pKD_kin  = 5.05
k_off    = 0.38
k_on     = 42957
R2_eq    = 0.9975
R2_off   = 0.9954
R2_on    = 0.9781
Rmax_pc  = 7
concl.   = ACCEPTED_KIN_EQ
pKD_final = 5.05
```

CP5-H5/Viet

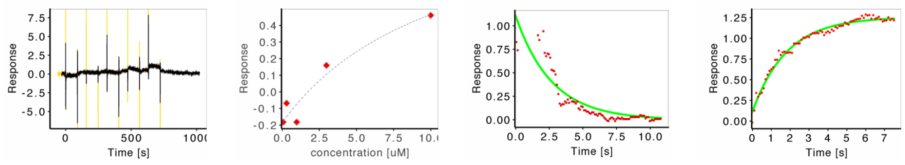


```
pKD_eq   = 4.95
pKD_kin  = 4.67
k_off    = 0.3738
k_on     = 17597
R2_eq    = 0.9271
R2_off   = 0.8685
R2_on    = 0.9804
Rmax_pc  = 2
concl.   = ACCEPTED_EQ
pKD_final = 4.95
```

CP6-H5/Viet

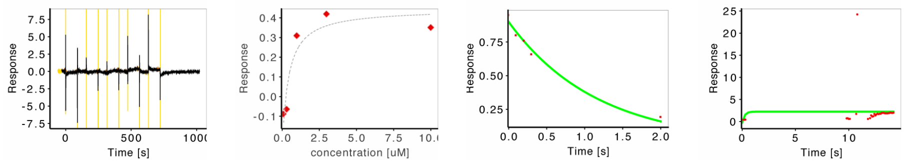


```
pKD_eq   = 6.39
pKD_kin  = 5.62
k_off    = 0.8625
k_on     = 360196
R2_eq    = 0.897
R2_off   = 0.9979
R2_on    = 1.37e-002
Rmax_pc  = 2.2
concl.   = REJECTED
pKD_final = 0
```

CP7-H5/Viet

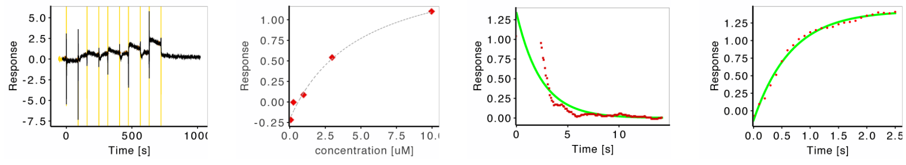


```
pKD_eq   = 5.31
pKD_kin  = 5.42
k_off    = 0.4159
k_on     = 109825
R2_eq    = 0.9889
R2_off   = 0.8315
R2_on    = 0.9903
Rmax_pc  = 4.6
concl.   = ACCEPTED_EQ
pKD_final = 5.31
```

Fig. S4 (ctd.)

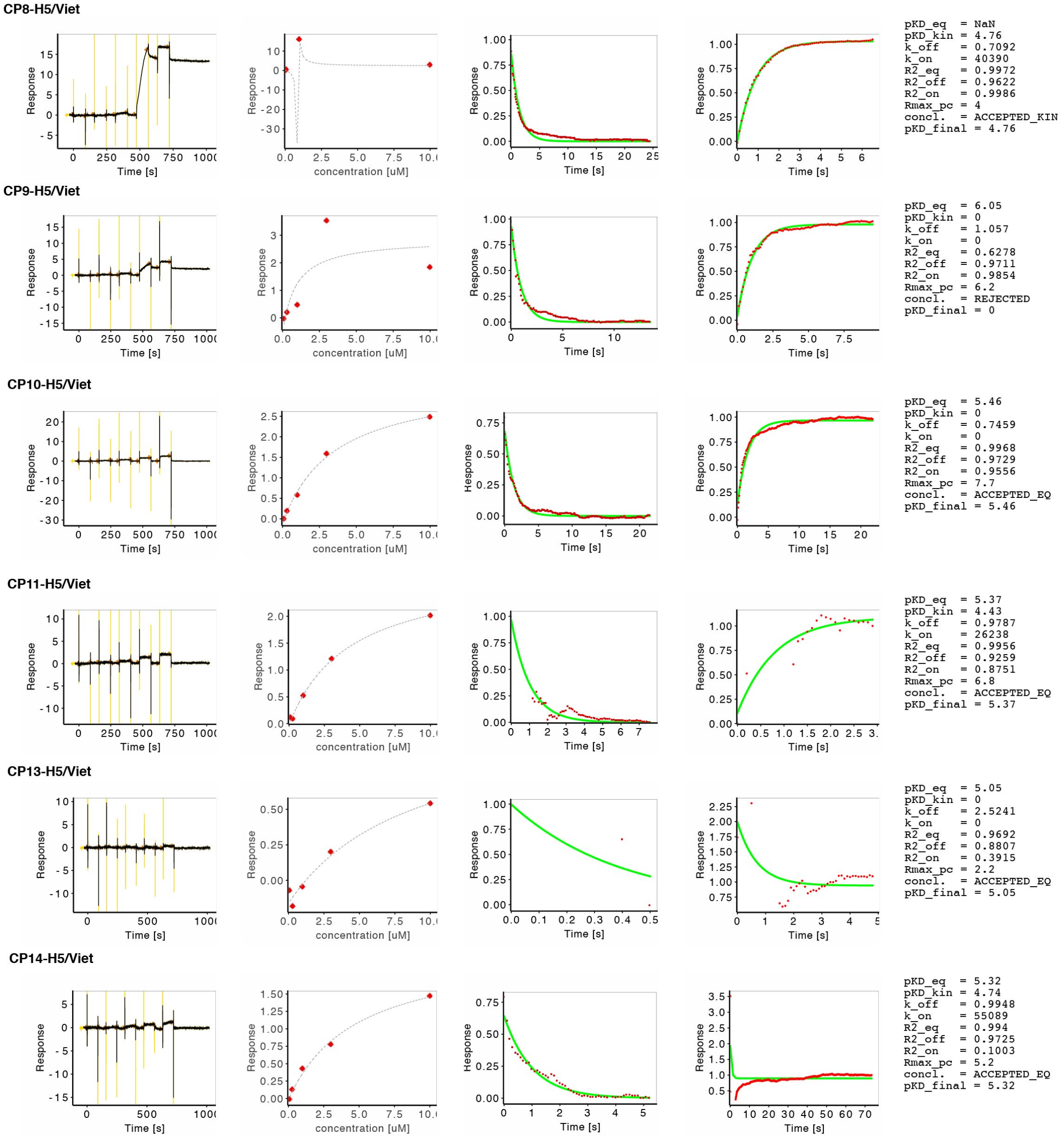
CP8-H5/Viet
pKD_eq = NaN
pKD_kin = 4.76
k_off = 0.7092
k_on = 40390
R2_eq = 0.9972
R2_off = 0.9622
R2_on = 0.9986
Rmax_pc = 4
concl. = ACCEPTED_KIN
pKD_final = 4.76
CP9-H5/Viet
pKD_eq = 6.05
pKD_kin = 0
k_off = 1.057
k_on = 0
R2_eq = 0.6278
R2_off = 0.9711
R2_on = 0.9854
Rmax_pc = 6.2
concl. = REJECTED
pKD_final = 0
CP10-H5/Viet
pKD_eq = 5.46
pKD_kin = 0
k_off = 0.7459
k_on = 0
R2_eq = 0.9968
R2_off = 0.9729
R2_on = 0.9556
Rmax_pc = 7.7
concl. = ACCEPTED_EQ
pKD_final = 5.46
CP11-H5/Viet
pKD_eq = 5.37
pKD_kin = 4.43
k_off = 0.9787
k_on = 26238
R2_eq = 0.9956
R2_off = 0.9259
R2_on = 0.8751
Rmax_pc = 6.8
concl. = ACCEPTED_EQ
pKD_final = 5.37
CP13-H5/Viet
pKD_eq = 5.05
pKD_kin = 0
k_off = 2.5241
k_on = 0
R2_eq = 0.9692
R2_off = 0.8807
R2_on = 0.3915
Rmax_pc = 2.2
concl. = ACCEPTED_EQ
pKD_final = 5.05
CP14-H5/Viet
pKD_eq = 5.32
pKD_kin = 4.74
k_off = 0.9948
k_on = 55089
R2_eq = 0.994
R2_off = 0.9725
R2_on = 0.1003
Rmax_pc = 5.2
concl. = ACCEPTED_EQ
pKD_final = 5.32

Supplementary Fig. 5. Statistical comparison of binding data of peptides in surface plasmon resonance (SPR) and AlphaLISA assay. HAs from H1 and H5 strains: H1/Cal = A/California/07/2009 (H1N1), H1/NCa = A/New Caledonia/20/1999 (H1N1), and H5/Viet = (A/Vietnam/1203/2004 (H5N1).

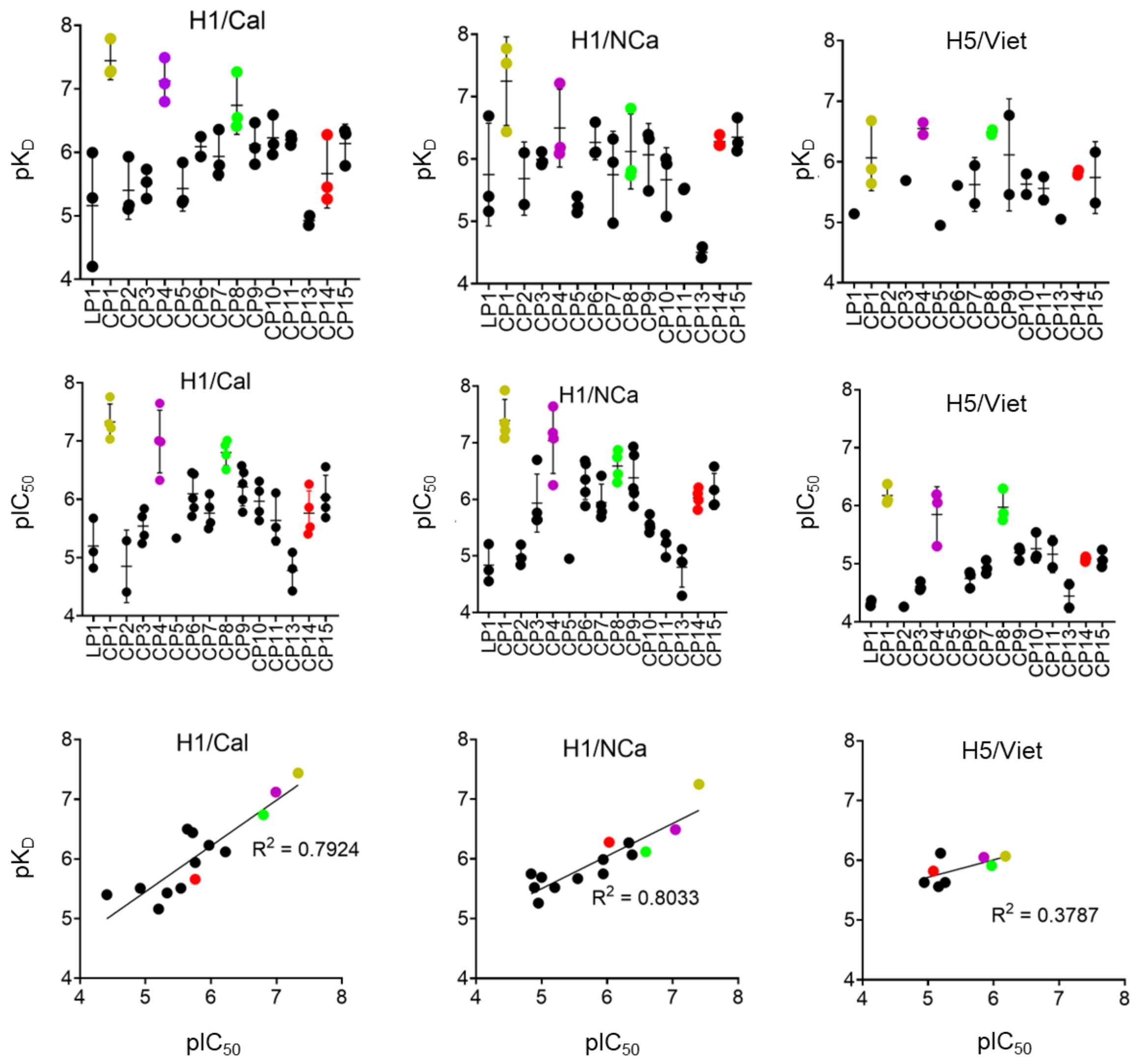

Supplementary Fig. 6. Relationship between peptide loop length and A) binding data, and B) neutralization data from HA from H1 strain: H1/NCa = A/New Caledonia/20/1999 (H1N1).

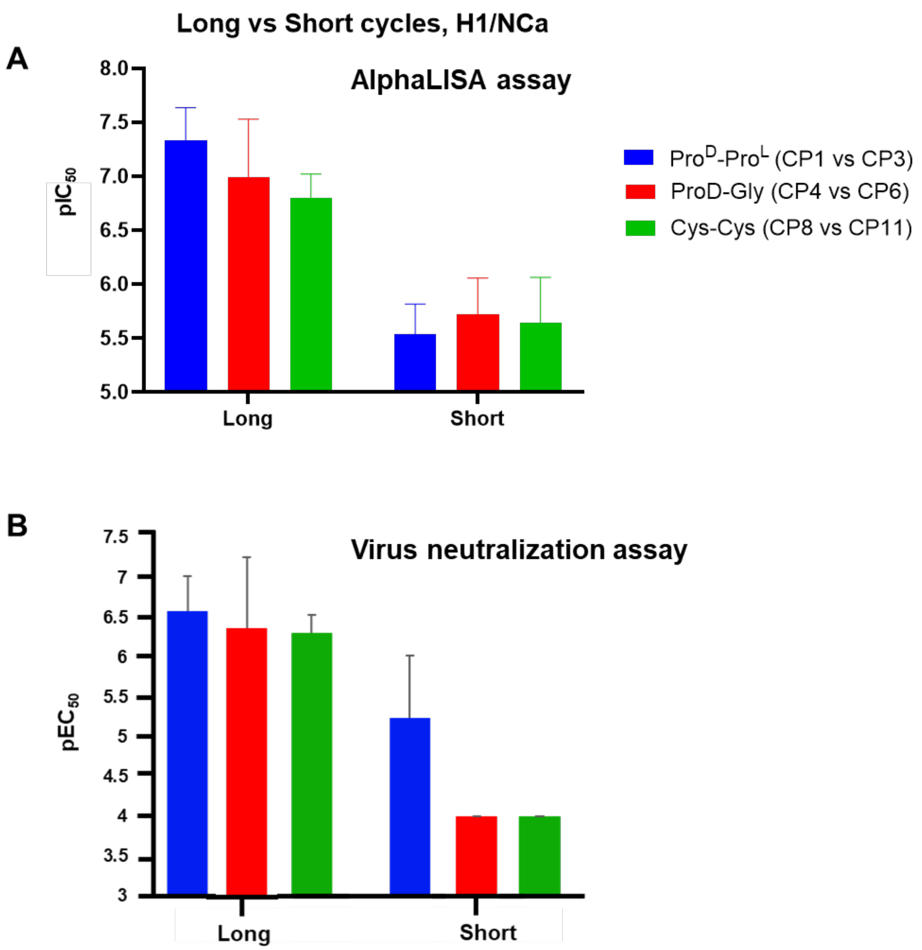

Supplementary Fig. 7. 2Fo-Fc electron density maps contoured at 1.0-1.5σ for designed peptides in complex with H1/PR8 and H5/Viet04 HAs. Cyclized peptides (**A**) CP1-H1/PR8 (1.5σ), (**B**) CP1-H5/Viet (1.0σ), (**C**) CP8-H1/PR8 (1.5σ) and (**D**) CP14-H1/PR8 (1.0σ). In the CP8-H1/PR8 complex, cystine residues are found in two alternate conformations. The peptide C, O, N and S atoms are represented in green/red/blue and yellow sticks, respectively. Electron density maps are represented in a light blue mesh. Succinic acid is labeled as SIN.

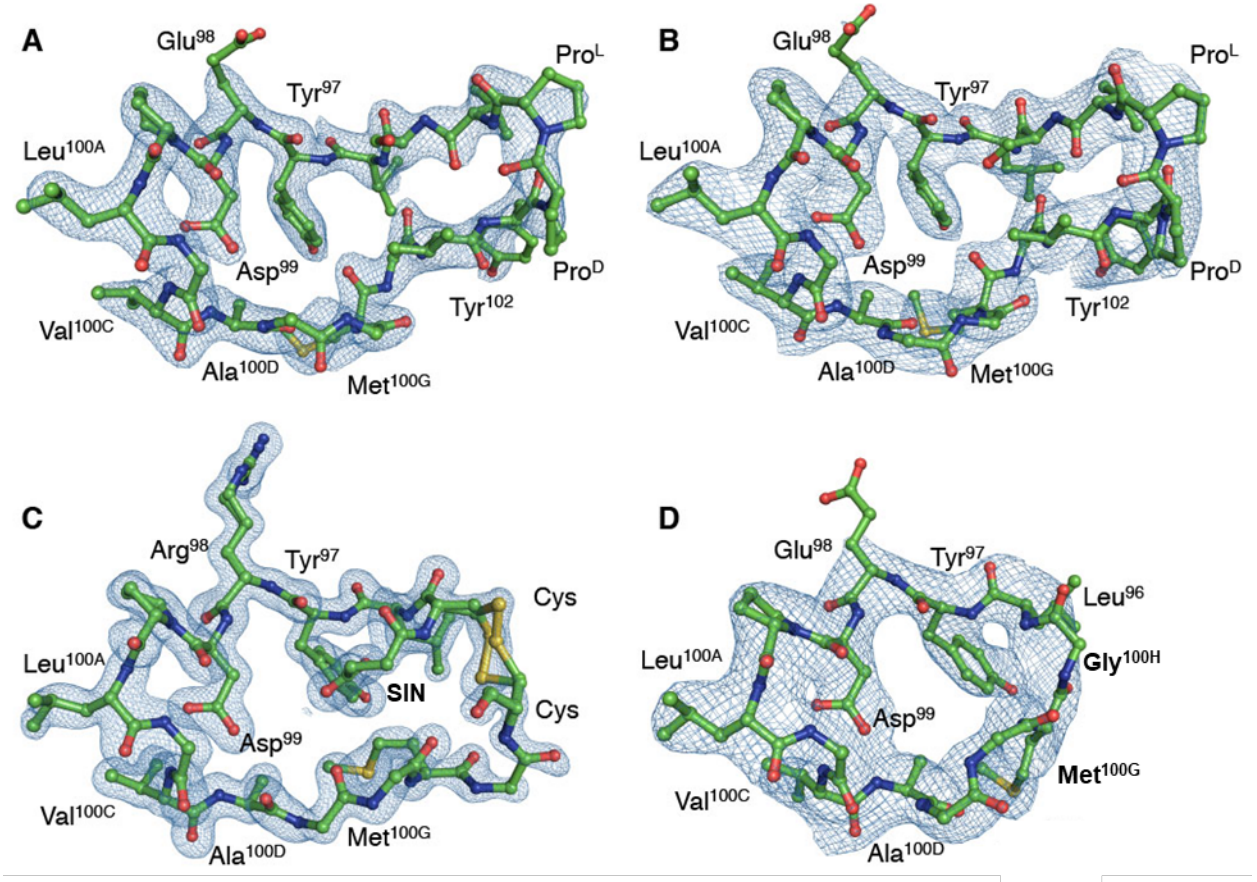

Supplementary Fig. 8. Simulated annealing (SA) omit maps contoured at 2.5σ for peptides in complex with H1/PR8 and H5/Viet04 HAs. (**A**) CP1-H1/PR8, (**B**) CP1-H5/Viet, (**C**) CP8-H1/PR8 and (**D**) CP14-H1/PR8. In the CP8-H1/PR8 complex, cystine residues are found in two alternate conformations. The peptide C, O, N and S atoms are represented in magenta/red/blue and yellow sticks, respectively. Electron density maps are represented in a light blue mesh. The peptides were not included during refinement. Succinic acid is labeled as SIN.

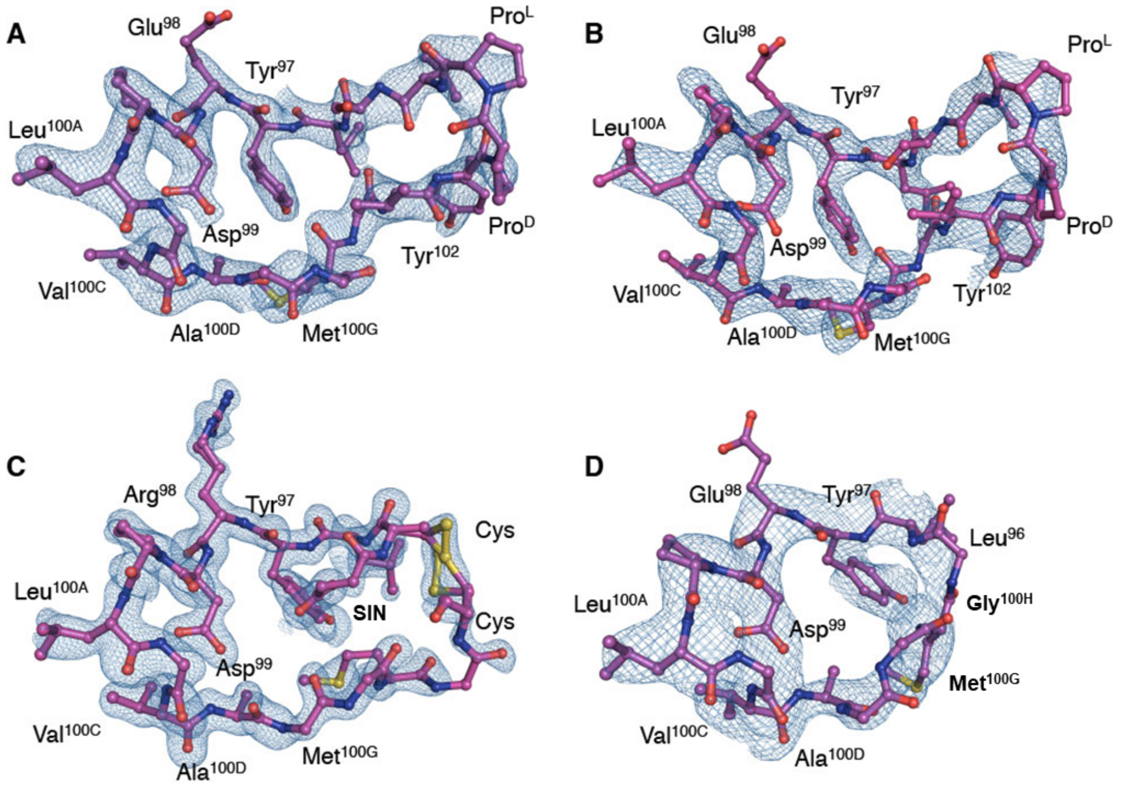

Supplementary Fig. 9. Comparison of binding mode of peptide CP1 to H1/PR8 versus human V_H1-69 broadly neutralizing antibodies (bnAbs) 27F3, F10, CR9114 and CR6261. V_H1-69 bnAbs insert a conserved germline-encoded Tyr (D or J region) and make conserved interactions with a hydrophobic pocket in the HA stem (grey surface with tube representation for HA backbone and sticks for HA2 Trp^{21}. Peptide CP1 and its parent bnAb sdAb38 also insert Tyr^{97} in the same position and orientation onto the HA as the bnAbs.

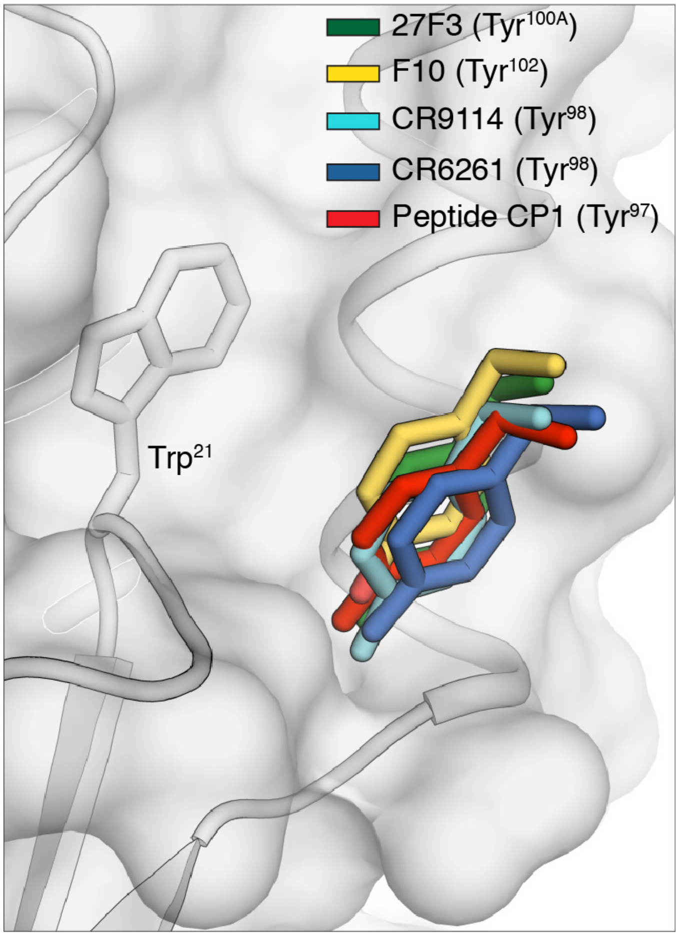

Supplementary Fig. 10. Comparison of peptide CP1 bound structures of H1/PR8 and H5/Viet HAs. (**a**) The binding mode of peptide CP1 (red tube with sticks) on H1/PR8 HA (light grey cartoon). HA1 Val^{40} in the HA stem epitope is shown as a grey stick. (**b**) Superimposition of CP1 peptide-bound conformations of H1/PR8 (light grey) and H5/Viet (dark grey) HAs. (**c**) The binding mode of peptide CP1 (blue tube with sticks) on H5/Viet HA (dark grey cartoon). HA1 Gln^{40} in the HA stem epitope is shown as a yellow stick.

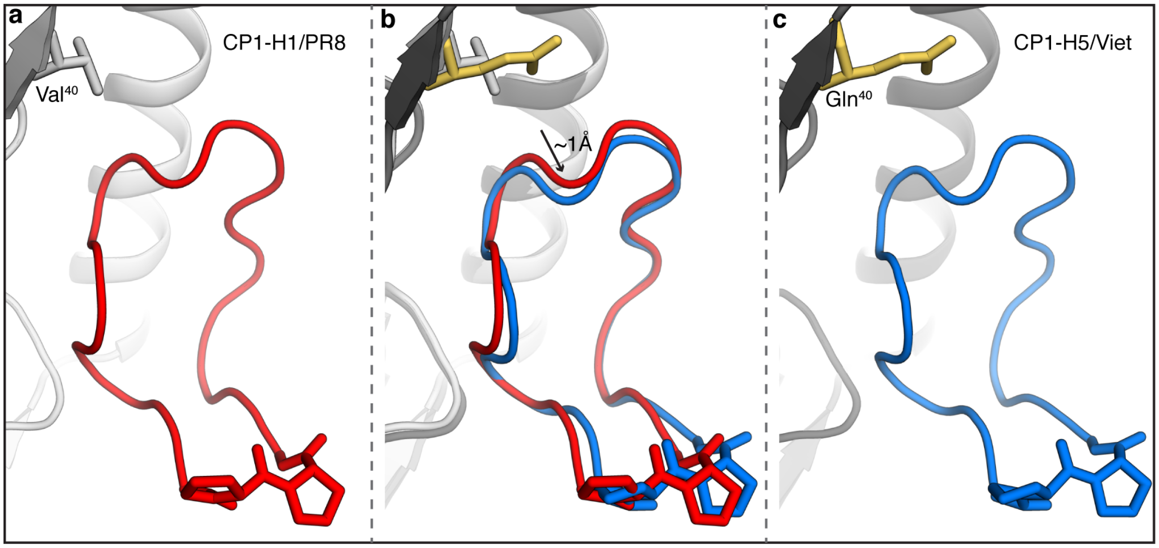

Supplementary Fig. 11. Surface plasmon resonance (SPR) sensorgrams for CP1-HA binding.

Binding kinetics of peptide CP1 against influenza A and B HAs. Group-1 HAs: H1/SC = A/South Carolina/1/1918, H1/PR8 = A/Puerto Rico/8/1934, H2/Jap = A/Japan/305/1957, H6/TM = A/turkey/Massachusetts/3740/ 1965, H9/TW = A/turkey/Wisconsin/1/1966, H11/DE = A/duck/England/1/1956, H12/DA = A/duck/Alberta/ 60/1976, H13/GM = A/gull/Maryland/704/1977, H16/BS = A/black-headed gull/Sweden /4/1999. Group-2 HAs: H3/HK68 = A/Hong Kong/1/1968, H3/Vict = A/Victoria/361/2011, H4/DC = A/duck/Czechoslovakia/1956, H7/Neth = A/Netherlands/219/2003, H10/CG = A/chicken/Germany/N/1949, H14/MA = A/mallard/Astrakhan/263/1982, H15/SWA = A/shearwater/W. Australia/2576/79. Influenza B HAs: FluB/Bris = B/Brisbane/33/2008 and FluB/Mas = B/Massachusetts/02/2012. Results of the experiment are summarized in Supplementary Table 7.

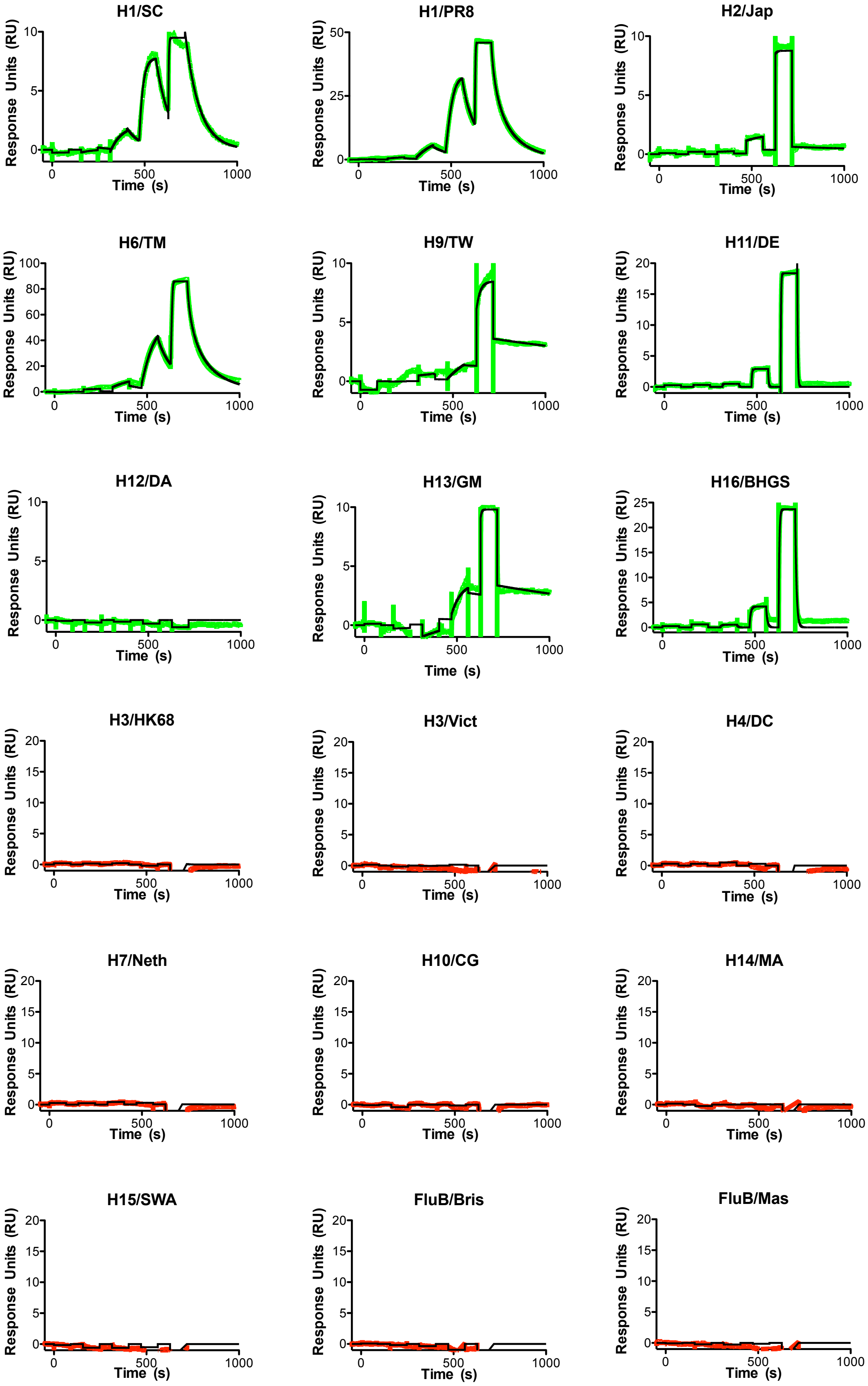
H1/SC
H1/PR8
H2/Jap
H6/TM
H9/TW
H11/DE
H12/DA
H13/GM
H16/BHGS
H3/HK68
H3/Vict
H4/DC
H7/Neth
H10/CG
H14/MA
H15/SWA
FluB/Bris
FluB/Mas
Response Units (RU)
Time (s)

Supplementary Fig. 12. Comparison of the H1/PR8 HA-peptide CP8 bound structure at pH 4.0 versus apo H1/PR8 and H1/Cal HA structures at pH 8. (A). Superimposition of CP8 peptide-bound conformations of PR8 HA (green) with apo H1/PR8 HA rendered in purple (PDB 1RU7) (RMSD 0.53 Å for all heavy atoms). (B) Superimposition of CP8 peptide-bound conformation of PR8 HA (green) with apo H1/Cal HA rendered in yellow (PDB 3LZG) (RMSD 0.76 Å for all heavy atoms). Only one protomer of the HA trimer is shown and the bound peptide is not shown for clarity.

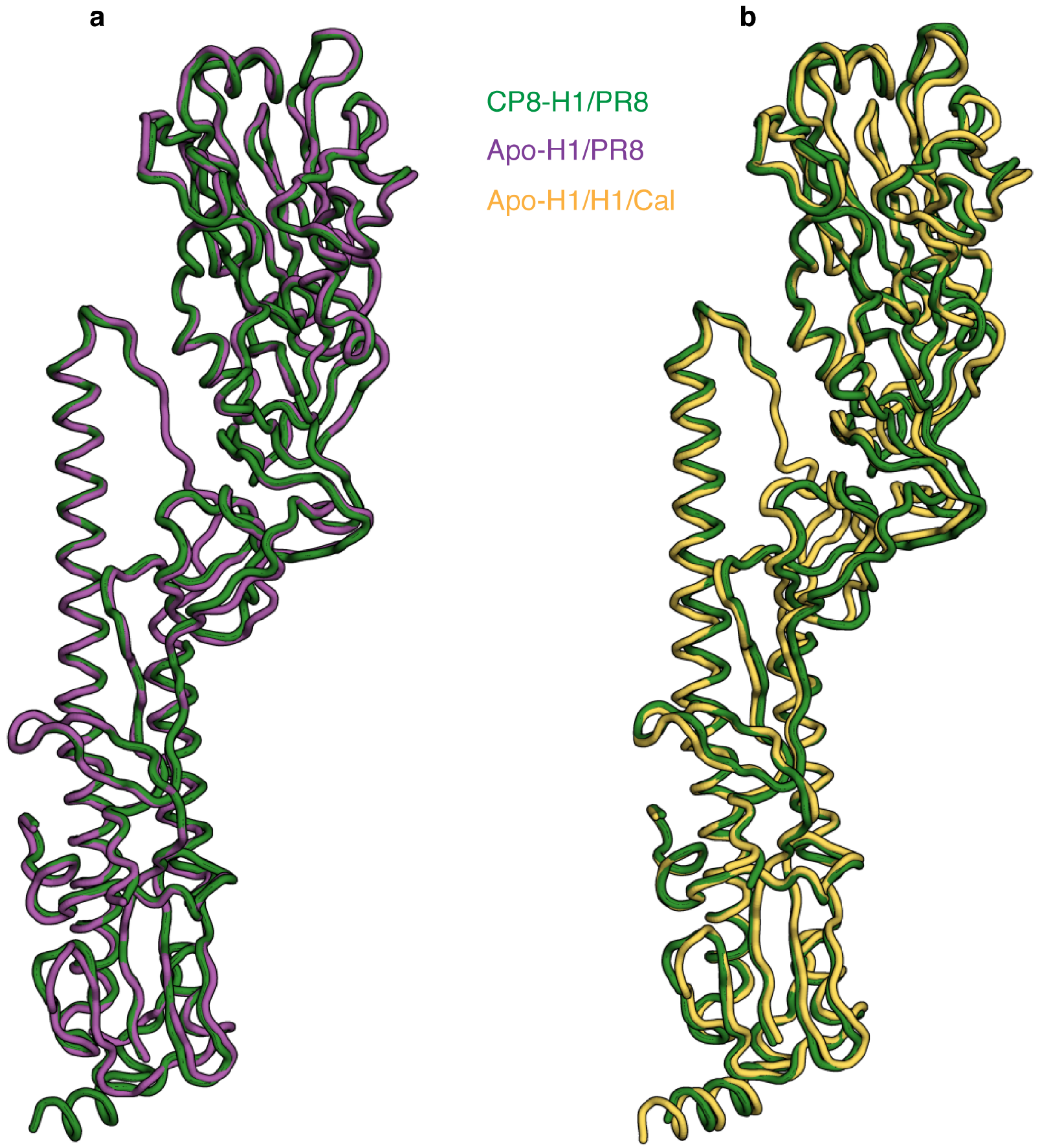
a
b
CP8-H1/PR8
Apo-H1/PR8
Apo-H1/H1/Cal

Supplementary Fig. 13. Calu-3 cell line toxicity data for the peptide ligands reported in Fig. 1c.

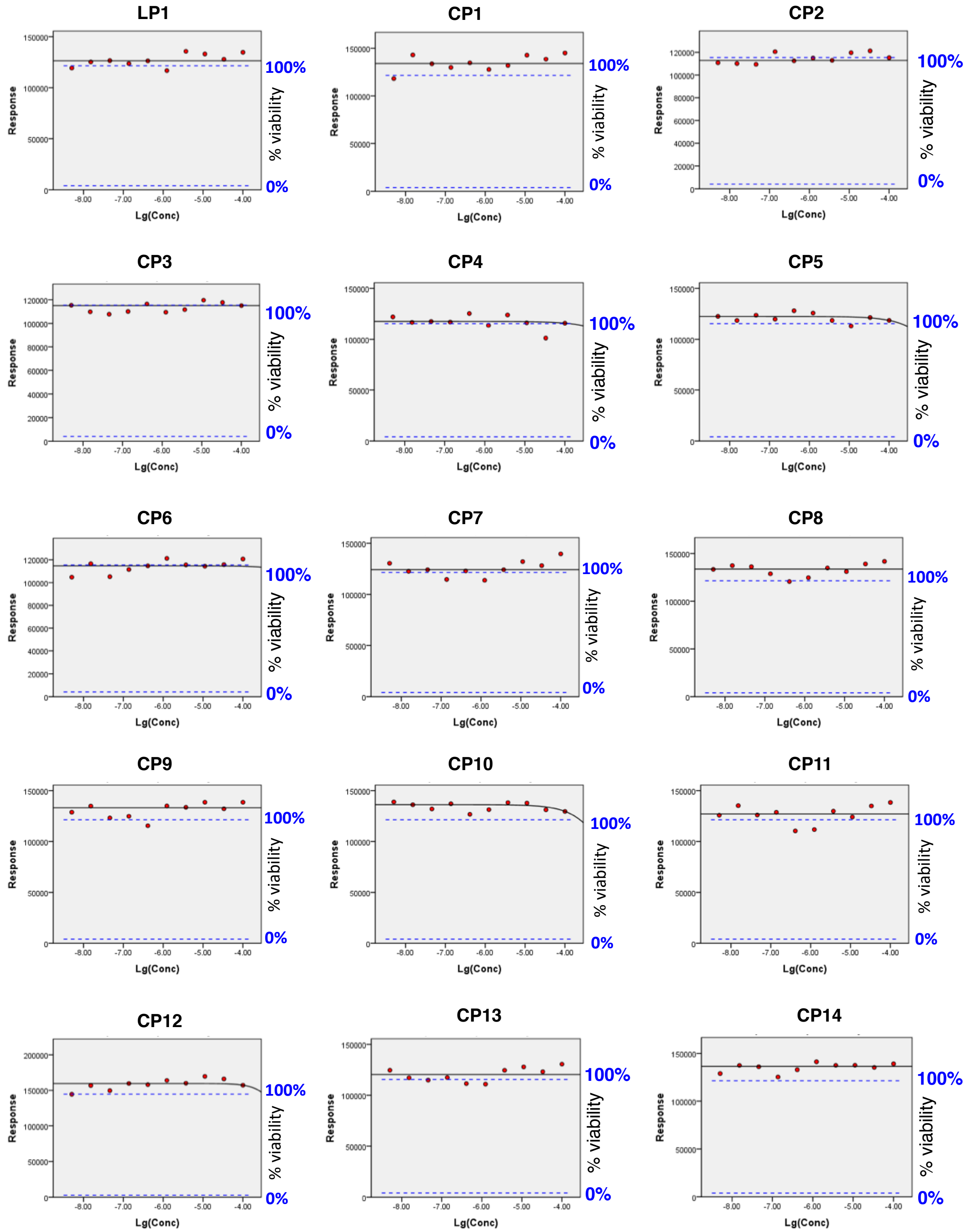

Supplementary Fig. 14. Amino acid sequences of influenza HA used in crystal structure determination and *in vitro* binding studies.

```
> A/South Carolina/1/1918 (H1N1)
DTICIGYHANNSTDTVDTVLEKNVTVTHSVNLLEDSHNGKLCKLKGIAPLQLGKCNIAGWLLGNPECDLLL
TASSWSYIVETSNSENGTCYPGDFIDYEELREQLSSVSSFEKFEIFPKTSSWPNHETTKGVTAACSYAGAS
SFYRNLLWLTKKGSSYPKLSKSYVNNKGKEVLVLWGVHHPPTGTDQQSLYQNADAYVSVGSSKYNRRFTPE
IAARPKVRDQAGRMNYYWTLLEPGDTITFEATGNLIAPWYAFALNRGSGSGIITSDAPVHDCNTKCQTPHG
AINSSLPFQNIHPVTIGECPKYVRSTKLRMATGLRNIPSIQSRGLFGAIAGFIEGGWTGMIDGWYGYHHQN
EQGSGYAADQKSTQNAIDGITNKVNSVIEKMNTQFTAVGKEFNNLERRIENLNKKVDDGFLDIWTYNAELL
VLLENERTLDFHDSNVRNLYEKVKSQLKNNAKEIGNGCFEFYHKCDDACMESVRNGTYDYPKYSEESKLNR
EEIDGV

>A/Puerto Rico/8/1934 (H1N1)
DTICIGYHANNSTDTVDTVLEKNVTVTHSVNLLEDSHNGKLCRLKGIAPLQLGKCNIAGWLLGNPECDPLL
PVRSWSYIVETPNSENGICYPGDFIDYEELREQLSSVSSFERFEIFPKESSWPNHNTNGVTAACSHEGKSS
FYRNLLWLTEKEGSYPKLKNSYVNKKGKEVLVLWGIHHPPNSKEQQNLYQNENAYVSVVTSNYNRRFTPEI
AERPKVRDQAGRMNYYWTLLKPGDTIIFEANGNLIAPMYAFALSRGFGSGIITSNASMHECNTKCQTPLGA
INSSLPYQNIHPVTIGECPKYVRSAKLRMVTGLRNIPSIQSRGLFGAIAGFIEGGWTGMIDGWYGYHHQNE
QGSGYAADQKSTQNAINGITNKVNTVIEKMNIQFTAVGKEFNKLEKRMENLNKKVDDGFLDIWTYNAELLV
LLENERTLDFHDSNVKNLYEKVKSQLKNNAKEIGNGCFEFYHKCDNECMESVRNGTYDYPKYSEESKLNRE
KVDGV

>A/duck/Alberta/345/1976 (H1N1)
DTICVGYHANNSTDTVDTVLEKNVTVTHSVNLLEDSHNGKLCSLNGIAPLQLGKCNVAGWLLGNPECDLLL
TANSWSYIIETSNSENGTCYPGEFIDYEELREQLSSISSFEKFEIFPKASSWPNHETTKGVTAACSYSGAS
SFYRNLLWITKKGTSYPKLSKSYTNNKGKEVLVLWGVHHPPSVSEQQSLYQNADAYVSVGSSKYNRRFAPE
IAARPEVRGQAGRMNYYWTLLDQGDTITFEATGNLIAPWYAFALNKGSDSGIITSDAPVHNCDTRCQTPHG
ALNSSLPFQNVHPITIGECPKYVKSTKLRMATGLRNVPSIQSRGLFGAIAGFIEGGWTGMIDGWYGYHHQN
EQGSGYAADQKSTQNAIDGITNKVNSVIEKMNTQFTAVGKEFNNLERRIENLNKKVDDGFLDVWTYNAELL
VLLENERTLDFHDSNVRNLYEKVKSQLRNNAKEIGNGCFEFYHKCDDECMESVKNGTYDYPKYSEESKLNR
EEID

>A/New Caledonia/20/1999 (H1N1)
DTICIGYHANNSTDTVDTVLEKNVTVTHSVNLLEDSHNGKLCRLKGIAPLQLGNCSVAGWILGNPECELLI
SKESWSYIVETPNPENGTCYPGYFADYEELREQLSSVSSFERFEIFPKESSWPNHTVTGVSASCSHNGKSS
FYRNLLWLTGKNGLYPNLSKSYVNNKEKEVLVLWGVHHPPNIGNQRALYHTENAYVSVVSSHYSRRFTPEI
AKRPKVRDQEGRINYYWTLLEPGDTIIFEANGNLIAPWYAFALSRGFGSGIITSNAPMDECDAKCQTPQGA
INSSLPFQNVHPVTIGECPKYVRSAKLRMVTGLRNIPSIQSRGLFGAIAGFIEGGWTGMVDGWYGYHHQNE
QGSGYAADQKSTQNAINGITNKVNSVIEKMNTQFTAVGKEFNKLERRMENLNKKVDDGFLDIWTYNAELLV
LLENERTLDFHDSNVKNLYEKVKSQLKNNAKEIGNGCFEFYHKCNNECMESVKNGTYDYPKYSEESKLNRE
KID

>A/Solomon Islands/3/2006 (H1N1)
DTICIGYHANNSTDTVDTVLEKNVTVTHSVNLLEDSHNGKLCRLKGIAPLQLGNCSVAGWILGNPECELLI
SRESWSYIVEKPNPENGTCYPGHFADYEELREQLSSVSSFERFEIFPKESSWPNHTTTGVSASCSHNGESS
FYKNLLWLTGKNGLYPNLSKSYANNKEKEVLVLWGVHHPPNIGDQRALYHKENAYVSVVSSHYSRKFTPEI
AKRPKVRDQEGRINYYWTLLEPGDTIIFEANGNLIAPRYAFALSRGFGSGIINSNAPMDECDAKCQTPQGA
INSSLPFQNVHPVTIGECPKYVRSAKLRMVTGLRNIPSIQSRGLFGAIAGFIEGGWTGMVDGWYGYHHQNE
QGSGYAADQKSTQNAINGITNKVNSVIEKMNTQFTAVGKEFNKLERRMENLNKKVDDGFIDIWTYNAELLV
LLENERTLDFHDSNVKNLYEKVKSQLKNNAKEIGNGCFEFYHKCNDECMESVKNGTYDYPKYSEESKLNRE
KID

>A/California/07/2009 (H1N1)
DTLCIGYHANNSTDTVDTVLEKNVTVTHSVNLLEDKHNGKLCKLRGVAPLHLGKCNIAGWILGNPECESLS
TASSWSYIVETPSSDNGTCYPGDFIDYEELREQLSSVSSFERFEIFPKTSSWPNHDSNKGVTAACPHAGAK
```

```
SFYKNLIWLVKKGNSYPKLSKSYINDKGKEVLVLWGIHHPSTSADQQSLYQNADAYVFVGSSRYSKKFKPE
IAIRPKVRXXEGRMNYYWTLVEPGDKITFEATGNLVVPRYAFAMERNAGSGIIISDTPVHDCNTTCQTPKG
AINTSLPFQNIHPITIGKCPKYVKSTKLRLATGLRNIPSIQSRGLFGAIAGFIEGGWTGMVDGWYGYHHQN
EQGSGYAADLKSTQNAIDEITNKVNSVIEKMNTQFTAVGKEFNHLEKRIENLNKKVDDGFLDIWTYNAELL
VLLENERTLDYHDSNVKNLYEKVRSQLKNNAKEIGNGCFEFYHKCDNTCMESVKNGTYDYPKYSEEAKLNR
EEID

>A/Adachi/2/1957 (H2N2)
DQICIGYHANNSTEKVDTILERNVTVTHAKDILEKTHNGKLCKLNGIPPLELGDCSIAGWLLGNPECDRLL
SVPEWSYIMEKENPRNGLCYPGSFNDYEELKHLLSSVKHFEKVKILPKDRWTQHTTTGGSQACAVSGNPSF
FRNMVWLTKKGSDYPVAKGSYNNTSGEQMLIIWGVHHPIDETEQRTLYQNVGTYVSVGTSTLNKRSTPEIA
TRPKVNGLGSRMEFSWTLLDMWDTINFESTGNLIAPEYGFKISKRGSSGIMKTEGTLENCETKCQTPLGAI
NTTLPFHNVHPLTIGECPKYVKSEKLVLATGLRNVPQIESRGLFGAIAGFIEGGWQGMVDGWYGYHHSNDQ
GSGYAADKESTQKAFDGITNKVNSVIEKMNTQFEAVGKEFGNLERRLENLNKKMEDGFLDVWTYNAELLVL
MENERTLDFHDSNVKNLYDKVRMQLRDNVKELGNGCFEFYHKCDDECMNSVKNGTYDYPKYEEESKLNRNE
IK

>A/Japan/305/1957 (H2N2)
DQICIGYHANNSTEKVDTILERNVTVTHAKDILEKTHNGKLCKLNGIPPLELGDCSIAGWLLGNPECDRLL
SVPEWSYIMEKENPRDGLCYPGSFNDYEELKHLLSSVKHFEKVKILPKDRWTQHTTTGGSRACAVSGNPSF
FRNMVWLTEKGSNYPVAKGSYNNTSGEQMLIIWGVHHPNDETEQRTLYQNVGTYVSVGTSTLNKRSTPEIA
TRPKVNGQGGRMEFSWTLLDMWDTINFESTGNLIAPEYGFKISKRGSSGIMKTEGTLENCETKCQTPLGAI
NTTLPFHNVHPLTIGECPKYVKSEKLVLATGLRNVPQIESRGLFGAIAGFIEGGWQGMVDGWYGYHHSNDQ
GSGYAADKESTQKAFDGITNKVNSVIEKMNTQFEAVGKEFSNLERRLENLNKKMEDGFLDVWTYNAELLVL
MENERTLDFHDSNVKNLYDKVRMQLRDNVKELGNGCFEFYHKCDDECMNSVKNGTYDYPKYEEESKLNRNE
IK

>A/Vietnam/1203/2004 (H5N1)
DQICIGYHANNSTEQVDTIMEKNVTVTHAQDILEKKHNGKLCDLDGVKPLILRDCSVAGWLLGNPMCDEFI
NVPEWSYIVEKANPVNDLCYPGDFNDYEELKHLLSRINHFEKIQIIPKSSWSSHEASLGVSSACPYQGKSS
FFRNVVWLIKKNSTYPTIKRSYNNTNQEDLLVLWGIHHPNDAAEQTKLYQNPTTYISVGTSTLNQRLVPRI
ATRSKVNGQSGRMEFFWTILKPNDAINFESNGNFIAPEYAYKIVKKGDSTIMKSELEYGNCNTKCQTPMGA
INSSMPFHNIHPLTIGECPKYVKSNRLVLATGLRNSPQRERRRKKRGLFGAIAGFIEGGWQGMVDGWYGYH
HSNEQGSGYAADKESTQKAIDGVTNKVNSIIDKMNTQFEAVGREFNNLERRIENLNKKMEDGFLDVWTYNA
ELLVLMENERTLDFHDSNVKNLYDKVRLQLRDNAKELGNGCFEFYHKCDNECMESVRNGTYDYPQYSEEAR
LKREEIS

>A/turkey/Massachusetts/3740/1965 (H6N2)
DKICIGYHANNSTTQVDTILEKNVTVTHSVELLESQKEERFCRVLNKTPLDLKGCTIEGWILGNPQCDILL
GDQSWSYIVERPGAQNGICYPGVLNEVEELKAFIGSGEKVQRFEMFPKSTWTGVDTNSGVTRACPYTTSGS
SFYRNLLWIIKTRSAAYPVIKGTYNNTGSQPILYFWGVHHPPNTDEQNTLYGSGDRYVRMGTESMNFAKSP
EIAARPAVNGQRGRIDYYWSVLKPGETLNVESNGNLIAPWYAYKFTSSNNKGAIFKSNLPIENCDAVCQTV
AGALKTNKTFQNVSPLWIGECPKYVKSESLRLATGLRNVPQAETRGLFGAIAGFIEGGWTGMIDGWYGYHH
ENSQGSGYAADKESTQKAIDGITNKVNSIIDKMNTQFEAVEHEFSNLERRIDNLNKRMEDGFLDVWTYNAE
LLVLLENERTLDLHDANVKNLYEKVKSQLRDNAKDLGNGCFEFWHKCDDECINSVKNGTYDYPKYQDESKL
NRQEIDSV

>A/turkey/Wisconsin/1/1966 (H9N2)
DKICIGYQSTNSTETVDTLTESNVPVTHTKELLHTEHNGMLCATDLGHPLILDTCTIEGLIYGNPSCDILL
GGKEWSYIVERSSAVNGMCYPGNVENLEELRSLFSSAKSYKRIQIFPDKTWNVTYSGTSRACSNSFYRSMR
WLTHKSNSYPFQNAHYTNNERENILFMWGIHHPPTDTEQTDLYKNADTTTSVTTEDINRTFKPVIGPRPLV
NGQQGRIDYYWSVLKPGQTLRIRSNGNLIAPWYGHVLTGESHGRILKTDLNNGNCVVQCQTEKGGLNTTLP
FHNISKYAFGNCPKYVGVKSLKLAVGLRNVPAVSSRGLFGAIAGFIEGGWPGLVAGWYGFQHSNDQGVGMA
ADKGSTQKAIDKITSKVNNIIDKMNKQYEVIDHEFNELEARLNMINNKIDDQIQDIWAYNAELLVLLENQK
TLDEHDANVNNLYNKVKRALGSNAVEDGNGCFELYHKCDDQCMETIRNGTYDRQKYQEESRLERQKIEGV
```

```
>A/duck/England/1/1956 (H11N6)
DEICIGYLSNNSTDKVDTIIENNVTVTSSVELVETEHTGSFCSINGKQPISLGDCSFAGWILGNPMCDELI
GKTSWSYIVEKPNPTNGICYPGTLESEEELRLKFSGVLEFNKFEVFTSNGWGAVNSGVGVTAACKFGGSNS
FFRNMVWLIHQSGTYPVIKRTFNNTKGRDVLIVWGIHHPATLTEHQDLYKKDSSYVAVGSETYNRRFTPEI
NTRPRVNGQAGRMTFYWKIVKPGESITFESNGAFLAPRYAFEIVSVGNGKLFRSELNIESCSTKCQTEIGG
INTNKSFHNVHRNTIGDCPKYVNVKSLKLATGPRNVPAIASRGLFGAIAGFIEGGWPGLINGWYGFQHRNE
EGTGIAADKESTQKAIDQITSKVNNIVDRMNTNFESVQHEFSEIEERINQLSKHVDDSVVDIWSYNAQLLV
LLENEKTLDLHDSNVRNLHEKVRRMLKDNAKDEGNGCFTFYHKCDNKCIERVRNGTYDHKEFEEESKINRQ
EIEGV

>A/duck/Alberta/60/1976 (H12N5)
DKICIGYQTNNSTETVNTLSEQNVPVTQVEELVHGGIDPILCGTELGSPLVLDDCSLEGLILGNPKCDLYL
NGREWSYIVERPKEMEGVCYPGSIENQEELRSLFSSIKKYERVKMFDFTKWNVTYTGTSKACNNTSNQGSF
YRSMRWLTLKSGQFPVQTDEYKNTRDSDIVFTWAIHHPPTSDEQVKLYKNPDTLSSVTTDEINRSFKPNIG
PRPLVRGQQGRMDYYWAVLKPGQTVKIQTNGNLIAPEYGHLITGKSHGRILKNNLPMGQCVTECQLNEGVM
NTSKPFQNTSKHYIGKCPKYIPSGSLKLAIGLRNVPQVQDRGLFGAIAGFIEGGWPGLVAGWYGFQHQNAE
GTGIAADRDSTQRAIDNMQNKLNNVIDKMNKQFEVVNHEFSEVESRINMINSKIDDQITDIWAYNAELLVL
LENQKTLDEHDANVRNLHDRVRRVLRENAIDTGDGCFEILHKCDNNCMDTIRNGTYNHKEYEEESKIERQK
VNGV

>A/gull/Maryland/704/1977 (H13N6)
DRICVGYLSTNSSERVDTLLENGVPVTSSIDLIETNHTGTYCSLNGVSPVHLGDCSFEGWIVGNPACTSNF
GIREWSYLIEDPAAPHGLCYPGELNNNGELRHLFSGIRSFSRTELIPPTSWGEVLDGTTSACRDNTGTNSF
YRNLVWFIKKNNRYPVISKTYNNTTGRDVLVLWGIHHPVSVDETKTLYVNSDPYTLVSTKSWSEKYKLETG
VRPGYNGQRSWMKIYWSLIHPGEMITFESNGGFLAPRYGYIIEEYGKGRIFQSRIRMSRCNTKCQTSVGGI
NTNRTFQNIDKNALGDCPKYIKSGQLKLATGLRNVPAISNRGLFGAIAGFIEGGWPGLINGWYGFQHQNEQ
GTGIAADKESTQKAIDQITTKINNIIDKMNGNYDSIRGEFNQVEKRINMLADRIDDAVTDIWSYNAKLLVL
LENDKTLDMHDANVKNLHEQVRRELKDNAIDEGNGCFELLHKCNDSCMETIRNGTYDHTEYAEESKLKRQE
IDGI

>A/black-headed gull/Sweden/4/1999 (H16N3)
DKICIGYLSNNSTDTVDTLTENGVPVTSSIDLVETNHTGTYCSLNGVSPIHLGDCSFEGWIVGNPSCASNI
NIREWSYLIEDPNAPHKLCFPGEVDNNGELRHLFSGVNSFSRTELIPPSKWGDILEGTTASCQNRGANSFY
RNLIWLVNKLNKYPVVKGEYNNTTGRDVLVLWGIHHPDTEATANKLYVNKNPYTLVSTKEWSRRYELEIGT
RIGDGQRSWMKIYWHLMHPGERITFESSGGLLAPRYGYIIEKYGTGRIFQSGVRLAKCNTKCQTSMGGINT
NKTFQNIERNALGDCPKYIKSGQLKLATGLRNVPSIVERGLFGAIAGFIEGGWPGLINGWYGFQHQNEQGT
GIAADKTSTQKAINEITTKINNIIEKMNGNYDSIRGEFNQVEKRINMIADRVDDAVTDIWSYNAKLLVLIE
NDRTLDLHDANVRNLHEQIKRALKDNAIDEGDGCFSILHKCNDSCMETIRNGTYNHEDYKEESQLKRQEIE
GI

>A/Hong Kong/1/1968 (H3N2)
ATLCLGHHAVPNGTLVKTITDDQIEVTNATELVQSSSTGKICNNPHRILDGIDCTLIDALLGDPHCDVFQN
ETWDLFVERSKAFSNCYPYDVPDYASLRSLVASSGTLEFITEGFTWTGVTQNGGSNACKRGPGSGFFSRLN
WLTKSGSTYPVLNVTMPNNDNFDKLYIWGVHHPSTNQEQTSLYVQASGRVTVSTRRSQQTIIPNIGSRPWV
RGLSSRISIYWTIVKPGDVLVINSNGNLIAPRGYFKMRTGKSSIMRSDAPIDTCISECITPNGSIPNDKPF
QNVNKITYGACPKYVKQNTLKLATGMRNVPEKQTRGLFGAIAGFIENGWEGMIDGWYGFRHQNSEGTGQAA
DLKSTQAAIDQINGKLNRVIEKTNEKFHQIEKEFSEVEGRIQDLEKYVEDTKIDLWSYNAELLVALENQHT
IDLTDSEMNKLFEKTGRQLRENAEDMGNGCFKIYHKCDNACIESIRNGTYDHDVYRDEALNNRFQIKGV

>A/Victoria/361/2011 (H3N2)
ATLCLGHHAVPNGTIVKTITNDQIEVTNATELVQNSSIGEICDSPHQILDGENCTLIDALLGDPQCDGFQN
KKWDLFVERSKAYSNCYPYDVPDYASLRSLVASSGTLEFNNESFNWTGVTQNGTSSACIRRSNNSFFSRLN
WLTHLNFKYPALNVTMPNNEQFDKLYIWGVHHPGTDKDQIFLYAQSSGRITVSTKRSQQAVIPNIGSRPRI
RNIPSRISIYWTIVKPGDILLINSTGNLIAPRGYFKIRSGKSSIMRSDAPIGKCNSECITPNGSIPNDKPF
QNVNRITYGACPRYVKQSTLKLATGMRNVPEKQTRGIFGAIAGFIENGWEGMVDGWYGFRHQNSEGRGQAA
DLKSTQAAIDQINGKLNRLIGKTNEKFHQIEKEFSEVEGRIQDLEKYVEDTKIDLWSYNAELLVALENQHT
IDLTDSEMNKLFEKTKKQLRENAEDMGNGCFKIYHKCDNACIGSIRNGTYDHDVYRDEALNNRFQIKGV
```

>A/duck/Czechoslovakia/1956 (H4N6)
PVICMGHHAVANGTMVKTLADDQVEVVTAQELVESQNLPELCPSPLRLVDGQTCDIINGALGSPGCDHLNG
AEWDVFIERPNAVDTCYPFDVPEYQSLRSILANNGKFEFIAEEFQWNTVKQNGKSGACKRANVNDFFNRLN
WLVKSDGNAYPLQNLTKINNGDYARLYIWGVHHPSTDTEQTNLYKNNPGRVTVSTKTSQTSVVPNIGSRPL
VRGQSGRVSFYWTIVEPGDLIVFNTIGNLIAPRGHYKLNNQKKSTILNTAIPIGSCVSKCHTDKGSLSTTK
PFQNISRIAVGDCPRYVKQGSLKLATGMRNIPEKASRGLFGAIAGFIENGWQGLIDGWYGFRHQNAEGTGT
AADLKSTQAAIDQINGKLNRLIEKTNDKYHQIEKEFEQVEGRIQDLEKYVEDTKIDLWSYNAELLVALENQ
HTIDVTDSEMNKLFERVRRQLRENAEDKGNGCFEIFHKCDNNCIESIRNGTYDHDIYRDEAINNRFQIQGV

>A/Netherlands/219/2003 (H7N7)
DKICLGHHAVSNGTKVNTLTERGVEVVNATETVERTNVPRICSKGKRTVDLGQCGLLGTITGPPQCDQFLE
FSADLIIERREGSDVCYPGKFVNEEALRQILRESGGIDKETMGFTYSGIRTNGTTSACRRSGSSFYAEMKW
LLSNTDNAAFPQMTKSYKNTRKDPALIIWGIHHSGSTTEQTKLYGSGNKLITVGSSNYQQSFVPSPGARPQ
VNGQSGRIDFHWLILNPNDTVTFSFNGAFIALDRASFLRGKSMGIQSEVQVDANCEGDCYHSGGTIISNLP
FQNINSRAVGKCPRYVKQESLLLATGMKNVPEIPKRRRRGLFGAIAGFIENGWEGLIDGWYGFRHQNAQGE
GTAADYKSTQSAIDQITGKLNRLIEKTNQQFELIDNEFTEVERQIGNVINWTRDSMTEVWSYNAELLVAME
NQHTIDLADSEMNKLYERVKRQLRENAEEDGTGCFEIFHKCDDDCMASIRNNTYDHSKYREEAIQNRIQID
PV

>A/chicken/Germany/N/1949 (H10N7)
DRICLGHHAVANGTIVKTLTNEQEEVTNATETVESTNLNKLCMKGRSYKDLGNCHPVGMLIGTPVCDPHLT
GTWDTLIERENAIAHCYPGATINEEALRQKIMESGGISKMSTGFTYGSSINSAGTTKACMRNGGDSFYAEL
KWLVSKTKGQNFPQTTNTYRNTDTAEHLIIWGIHHPSSTQEKNDLYGTQSLSISVESSTYQNNFVPVVGAR
PQVNGQSGRIDFHWTLVQPGDNITFSHNGGLIAPSRVSKLTGRGLGIQSEALIDNSCESKCFWRGGSINTK
LPFQNLSPRTVGQCPKYVNQRSLLLATGMRNVPEVVQGRGLFGAIAGFIENGWEGMVDGWYGFRHQNAQGT
GQAADYKSTQAAIDQITGKLNRLIEKTNTEFESIESEFSETEHQIGNVINWTKDSITDIWTYQAELLVAME
NQHTIDMADSEMLNLYERVRKQLRQNAEEDGKGCFEIYHTCDDSCMESIRNNTYDHSQYREEALLNRLNIN
SV

>A/mallard/Astrakhan/263/1982 (H14N5)
PIICLGHHAVENGTSVKTLTDNHVEVVSAKELVETNHTDELCPSPLKLVDGQDCDLINGALGSPGCDRLQD
TTWDVFIERPTAVDTCYPFDVPDYQSLRSILASSGSLEFIAEQFTWNGVKVDGSSSACLRGGRNSFFSRLN
WLTKATNGNYGPINVTKENTGSYVRLYLWGVHHPSSDNEQTDLYKVATGRVTVSTRSDQISIVPNIGSRPR
VRNQSGRISIYWTLVNPGDSIIFNSIGNLIAPRGHYKISKSTKSTVLKSDKRIGSCTSPCLTDKGSIQSDK
PFQNVSRIAIGNCPKYVKQGSLMLATGMRNIPGKQAKGLFGAIAGFIENGWQGLIDGWYGFRHQNAEGTGT
AADLKSTQAAIDQINGKLNRLIEKTNEKYHQIEKEFEQVEGRIQDLEKYVEDTKIDLWSYNAELLVALENQ
HTIDVTDSEMNKLFERVRRQLRENAEDQGNGCFEIFHQCDNNCIESIRNGTYDHNIYRDEAINNRIKINPV

>A/shearwater/Western Australia/2576/1979 (H15N9)
DKICLGHHAVANGTKVNTLTERGVEVVNATETVEITGIDKVCTKGKKAVDLGSCGILGTIIGPPQCDLHLE
FKADLIIERRNSSDICYPGRFTNEEALRQIIRESGGIDKESMGFRYSGIRTDGATSACKRTVSSFYSEMKW
LSSSMNNQVFPQLNQTYRNTRKEPALIVWGVHHSSSLDEQNKLYGTGNKLITVGSSKYQQSFSPSPGARPK
VNGQAGRIDFHWMLLDPGDTVTFTFNGAFIAPDRATFLRSNAPSGIEYNGKSLGIQSDAQIDESCEGECFY
SGGTINSPLPFQNIDSRAVGKCPRYVKQSSLPLALGMKNVPEKIRTRGLFGAIAGFIENGWEGLIDGWYGF
RHQNAQGQGTAADYKSTQAAIDQITGKLNRLIEKTNKQFELIDNEFTEVEQQIGNVINWTRDSLTEIWSYN
AELLVAMENQHTIDLADSEMNKLYERVRRQLRENAEEDGTGCFEIFHRCDDQCMESIRNNTYNHTEYRQEA
LQNRIMINPV

>B/Brisbane/33/2008
DRICTGITSSNSPHVVKTATQGEVNVTGVIPLTTTPTKSHFANLKGTETRGKLCPKCLNCTDLDVALGRPK
CTGKIPSARVSILHEVRPVTSGCFPIMHDRTKIRQLPNLLRGYEHIRLSTHNVINAENAPGGPYKIGTSGS
CPNITNGNGFFATMAWAVPKNDKNKTATNPLTIEVPYICTEGEDQITVWGFHSDSETQMAKLYGDSKPQKF
TSSANGVTTHYVSQIGGFPNQTEDGGLPQSGRIVVDYMVQKSGKTGTITYQRGILLPQKVWCASGRSKVIK
GSLPLIGEADCLHEKYGGLNKSKPYYTGEHAKAIGNCPIWVKTPLKLANGTKYRPPAKLLKERGFFGAIAG
FLEGGWEGMIAGWHGYTSHGAHGVAVAADLKSTQEAINKITKNLNSLSELEVKNLQRLSGAMDELHNEILE
LDEKVDDLRADTISSQIELAVLLSNEGIINSEDEHLLALERKLKKMLGPSAVEIGNGCFETKHKCNQTCLD
RIAAGTFDAGEFSLPTFDSLNITAAS

```
>B/Massachusetts/02/2012
DRICTGITSSNSPHVVKTATQGEVNVTGVIPLTTTPTKSYFANLKGTKTRGKLCPDCLNCTDLDVALGRPM
CVGTTPSAKASILHEVRPVTSGCFPIMHDRTKIRQLANLLRGYENIRLSTQNVIDAEKAPGGPYRLGTSGS
CPNATSKSGFFATMAWAVPKDNNKNATNPLTVEVPYICAEGEDQITVWGFHSDDKTQMKNLYGDSNPQKFT
SSANGVTTHYVSQIGGFPDQTEDGGLPQSGRIVVDYMMQKPGKTGTIVYQRGVLLPQKVWCASGRSKVIKG
SLPLIGEADCLHEKYGGLNKSKPYYTGEHAKAIGNCPIWVKTPLKLANGTKYRPPAKLLKERGFFGAIAGF
LEGGWEGMIAGWHGYTSHGAHGVAVAADLKSTQEAINKITKNLNSLSELEVKNLQRLSGAMDELHNEILEL
DEKVDDLRADTISSQIELAVLLSNEGIINSEDEHLLALERKLKKMLGPSAVDIGNGCFETKHKCNQTCLDR
IAAGTFNAGEFSLPTFDSLNITAAS
```

Supplementary Table 1. Comparative analysis of the CDR3 loop of single domain antibodies (sdAb) and conventional antibodies (Abs) against a diverse set of targets.

PDB IDs	Antibody name	Antibody type	Target	CDR3 length	Interacting residues in CDR3	(%) contacts of CDR3 residues	N atoms in paratope	Interacting atoms in CDR3	(%) contacts of CDR3 atoms
6FYT	SD38	sdAb	Influenza hemagglutinin	18	10	56%	48	32	67%
3K3Q	Aa1	sdAb	*C. botulinum* neurotoxin	19	9	47%	32	27	84%
6GS1	Nb00	sdAb	Transporter DtpA	18	7	39%	50	24	48%
6H16	L-P2-D07	sdAb	LRP6 P3E3P4E4	12	6	50%	49	27	55%
4FHB	Nb179	sdAb	Dihydrofolate reductase	22	9	41%	48	18	38%
4KRP	9G8	sdAb	EGFR	20	12	60%	63	46	73%
5IMK	N/A	sdAb	Complement receptor Vsig4	18	10	56%	55	43	78%
4TVS	BS1	sdAb	LAP1	20	9	45%	45	43	96%
4XT1	Nb7	sdAb	CX3CL1	12	7	58%	62	31	50%
5HVG	a204	sdAb	Thrombin-activatable fibrinolysis inhibitor	16	9	56%	49	40	82%
3P0G	Nb80	sdAb	β2 adrenergic receptor	14	7	50%	57	26	46%
5MWN	nbK18	sdAb	EAEC T6SS component TssK	13	9	69%	63	42	67%
5MWN	nbK27	sdAb	EAEC T6SS component TssK	13	7	54%	78	26	33%
3ZTN	FI6	Abs	Influenza hemagglutinin	22	8	36%	53	43	81%
5VJ6	PG9	Abs	HIV-1 env trimer	28	16	57%	130	71	55%
4FQR	C05	Abs	Influenza hemagglutinin	24	14	58%	184	59	32%
5GZN	Z3L1	Abs	Zika envelope protein	20	10	50%	53	37	70%
6HF1	Fab 316*	Abs	Sulfhydryl oxidase 1	9	2	22%	50	12	24%
6I8S	MEDI-579	Abs	Plasminogen activator inhibitor 1	14	4	29%	59	16	27%
3BQU	3H68*	Abs	2F5 Fab to HIV gp41	15	4	27%	42	13	31%
2CMR	D5	Abs	HIV gp41	12	5	42%	77	15	19%
1AHW	5G9*	Abs	Tissue factor	10	4	40%	64	10	16%
4KRP	Cetuximab	Abs	EGFR	13	5	38%	69	27	39%
2FJG	G6	Abs	VEGF	13	5	38%	63	22	35%
2J88	21E11*	Abs	Glucosaminidase	9	4	44%	67	9	13%
4O02	17E6*	Abs	AlphaVBeta3 integrin	11	4	36%	134	38	28%
5HDQ	305-78-7*	Abs	MntC	13	4	31%	56	18	32%
4DW2	mAb-112*	Abs	uPA	11	2	18%	79	12	15%

Abs are human and mouse*; Cetuximab is a chimeric antibody [variable domain (mouse) and constant domain (human)]. sdAbs are Llama- 6FYT, 3K3Q, 6GS1, 6H16, 4FHB, 4KRP, 3P0G, 5MWN and Alpaca- 5IMK, 4TVS, 4XT1, 5HVG.

Supplementary Table 2. AlphaLISA assay derived IC_{50} values for peptides binding to influenza groups 1 & 2 and influenza B.

Peptide ID	IC_{50} (µM)					
	**H1/Cal**	**H1/NCa**	**H5/Viet**	**H3/Bris**	**H7/Neth**	**B/Flo**
LP1	11.50±2.50	22.93±4.14	47.86±3.77	>100	>100	>100
CP1	0.055±0.00	0.052±0.02	0.84±0.13	>100	>100	>100
CP2	5.13*	12.70±1.64	55.11*	>100	>100	>100
CP3	1.71±0.19	2.01±0.54	27.14±2.79	>100	>100	>100
CP4	0.10±0.00	0.075±0.01	0.76±0.086	>100	>100	>100
CP5	4.63*	11.23*	N.D.	>100	>100	>100
CP6	1.09±0.20	1.47±0.32	13.49±2.17	>100	>100	>100
CP7	0.36±0.01	0.22±0.01	14.81±0.57	>100	>100	>100
CP8	0.11±0.00	0.16±0.02	1.55±0.29	>100	>100	>100
CP9	0.30±0.03	0.70±0.02	5.59±0.21	>100	>100	>100
CP10	0.61±0.08	2.93±0.32	7.61±1.56	>100	>100	>100
CP11	4.10±0.76	4.99±0.60	7.78±2.63	>100	>100	>100
CP12	6.38±0.17	4.95±0.38	28.82±10.21	>100	>100	>100
CP13	11.80±2.61	12.79±0.09	39.82±12.16	>100	>100	>100
CP14	1.72±0.24	1.24±0.15	10.03±0.91	>100	>100	>100

N.D. – not determined; values reported in the table are average of two independent experiments. The standard deviation of the errors is reported.*- values from a single experiment. Group-1 H1 HAs (H1/Cal = A/California/07/2009 (H1N1), H1/NCa = A/New Caledonia/20/1999 (H1N1)); H5 HA (H5/Viet = (A/Vietnam/1203/2004 (H5N1)); group-2 H3 HA (H3/Bris = A/Brisbane/10/2007 (H3N2)); H7 HA (H7/Neth = A/Netherlands/219/2003 (H7N7)) and influenza B (B/Flo = B/Florida/4/2006)

Supplementary Table 3. Virus neutralization assay (VNA) derived EC_{50} values for peptide binding to influenza groups 1 & 2 and influenza B.

Peptide ID	EC_{50} (µM)					
	**H1/Cal**	**H1/NCa**	**H5/Viet**	**H3/Bris**	**H7/Neth**	**B/Flo**
LP1	>100	>100	>100	>100	>100	>100
CP1	0.192*	0.10±0.02	49.95±4.30	>100	>100	>100
CP2	>100	75.42±7.08	>100	>100	>100	>100
CP3	39*	19.99±0.99	>100	>100	>100	>100
CP4	0.38±0.08	0.16±0.03	>100	>100	>100	>100
CP5	N.D.	>100	>100	>100	>100	>100
CP6	37.56±5.61	38.89±3.93	>100	>100	>100	>100
CP7	>100	>100	>100	>100	>100	>100
CP8	0.096±0.02	0.28±0.01	34.62±22.50	>100	>100	>100
CP9	75.12±6.17	0.82±0.00	>100	>100	>100	>100
CP10	4.96±2.48	88.27±23.07	>100	>100	>100	>100
CP11	20.58±3.47	>100	>100	>100	>100	>100
CP12	96.97±36.70	58.28±15.32	>100	>100	>100	>100
CP13	>100	>100	>100	>100	>100	>100
CP14	85.00±35.67	45.31±1.21	>100	>100	>100	>100

N.D. – not determined; values reported in the table are average of two independent experiments. The standard deviation of the errors is reported.*- values from a single experiment. Group-1 H1 (H1/Cal = A/California/07/2009 (H1N1), H1/NCa = A/New Caledonia/20/1999 (H1N1)); H5 (H5/Viet = (A/Vietnam/1203/2004 (H5N1)); group-2 H3 (H3/Bris = A/Brisbane/10/2007 (H3N2)); H7 (H7/Neth = A/Netherlands/219/2003 (H7N7)) and influenza B (B/Flo = B/Florida/4/2006) viruses.

Supplementary Table 4. ANOVA comparisons for peptides in AlphaLISA assay and SPR. Statistical analysis was performed using ordinary one-way ANOVA (GraphPad Prism). Data were log-transformed (pK_D or pIC_{50}) to achieve normal distributions. Equality of variances was confirmed by the Brown–Forsythe test ($p > 0.05$). When the ANOVA indicated a significant effect ($p < 0.05$), Tukey's multiple-comparison test was used to identify statistically significant differences between individual peptides. Statistical significance levels are indicated as follows: ns = $p > 0.05$ (not significant), * = $p < 0.05$, ** = $p < 0.01$, *** = $p < 0.001$, and **** = $p < 0.0001$.

ANOVA comparisons test	pIC_{50} H1/Cal	pIC_{50} H1/NCa	pIC_{50} H5/Viet	pK_D H1/Cal	pK_D H1/NCa	pK_D H5/Viet
LP1 vs. CP1	****	****	****	***	ns	ns
LP1 vs. CP4	****	****	****	**	ns	ns
LP1 vs. CP8	****	****	****	*	ns	ns
CP1 vs. CP2	****	****	****	**	ns	ns
CP1 vs. CP3	****	****	****	**	ns	ns
CP1 vs. CP14	****	***	***	*	ns	ns
CP3 vs. CP8	**	ns	****	ns	ns	ns
CP4 vs. CP6	*	ns	***	ns	ns	ns
CP4 vs. CP14	**	*	*	ns	ns	ns
CP6 vs. CP7	ns	ns	ns	ns	ns	ns
CP8 vs. CP11	*	***	ns	ns	ns	ns
CP8 vs. CP14	*	ns	**	ns	ns	ns

Supplementary Table 5. **Crystallization conditions for peptide ligands in complex with H1/PR8 and H5/Viet HAs**

Peptide ID	Crystallization Temperature (°C)	Crystallization Conditions
CP1	4	H1/PR8: 10 mg/ml,<br>0.2 M calcium acetate,<br>1 M HEPES, pH 7.4 ,<br>40% (v/v) polyethylene glycol 400.
CP1	20	H5/Viet: 10 mg/ml,<br>0.2 M di-sodium tartrate,<br>20% (w/v) polyethylene glycol 3350.
CP8	4	H1/PR8: 10 mg/ml,<br>0.2 M lithium sulfate,<br>20% (w/v) polyethylene glycol 1000,<br>0.1 M phosphate-citrate, pH 4.2.
CP14	4	H1/PR8: 10 mg/ml,<br>0.2 M magnesium nitrate,<br>20% (w/v) polyethylene glycol 3350.

Supplementary Table 6. Data collection and refinement statistics for peptide complexes with H1/PR8 HA and H5/Viet HA

	CP1-H1/PR8	CP14-H1/PR8	CP8-H1/PR8
**Data collection**			
Beamline	APS-23-IDD	APS-23-IDB	SSRL-12-2
Wavelength (Å)	1.0332	1.0331	0.9794
Space Group	C2	$I2_13$	H32
Unit cell (Å, °)	a=189.7, b=108.8, c=118.2; β =122.3	a=b=c=159.1	a=b=102.0, c=335.1
Resolution range (Å)[a]	47.96-2.03 (2.07-2.03)	37.52-2.87 (2.92-2.87)	42.10-1.59 (1.62-1.59)
Observations	408,669	272,770	384,284
Unique reflections	131,036 (6488)	15,428 (702)	89,949 (4486)
Completeness (%)	99.4 (98.8)	99.6 (92.5)	99.2 (99.8)
I/σ(I)	15.4 (1.3)	36.8 (1.5)	27.6 (2.4)
R_{sym}[b]	0.07 (0.89)	0.08 (0.59)	0.04 (0.39)
R_{pim}[c]	0.04 (0.49)	0.01 (0.28)	0.01 (0.19)
$CC_{1/2}$[d]	0.95 (0.81)	0.97 (0.79)	0.98 (0.91)
Redundancy	3.1 (2.9)	17.7 (3.4)	4.3 (4.1)
**Refinement**			
Resolution (Å)	47.96-2.03	37.52-2.87	42.10-1.59
No. reflections[e]	130,674 (6585)	15,419 (714)	89,937 (4551)
R_{cryst}[f]/R_{free}[g]	0.22/0.24	0.22/0.23	0.18/0.20
No. atoms			
Protein	11802	3930	3933
Peptide/glycan	402/345	88/84	129/112
Water /ions	377/3	10/-	592/15
Wilson B ($Å^2$)	37	70	19
Average B value ($Å^2$)			
Protein	54	70	28
Peptide ligand	65	78	25
Water/ions	53/40	58/-	40/61
**RMSD from ideal geometry**			
Bond length (Å)	0.008	0.005	0.012
Bond angle (°)	0.96	1.0	1.3
**Ramachandran Statistics (%)**[h]			
Favored	96.6	97.8	97.4
Outliers	0	0	0

Table S6 (ctd.)

	CP1- H5/Viet
**Data collection**	
Beamline	SSRL-12-2
Wavelength (Å)	1.0331
Space Group	P321
Unit cell (Å)	a=b=116.3, c=141.5
Resolution range (Å)[a]	47.19-2.76 (2.81-2.76)
Observations	261,606
Unique reflections	29,032 (1421)
Completeness (%)	99.9 (98.7)
I/σ(I)	28.9 (3.0)
R_{sym}[b]	0.11 (0.58)
R_{pim}[c]	0.03 (0.19)
$CC_{1/2}$[d]	0.98 (0.90)
Redundancy	9.0 (8.5)
**Refinement**	
Resolution (Å)	47.19-2.76
No. reflections[e]	29,018 (1473)
R_{cryst}[f]/R_{free}[g]	0.26/0.28
No. atoms	
Protein	3990
Peptide/glycan	134/56
Water /ions	70/-
Wilson B (Å^2)	53
Average B value (Å^2)	
Protein	52
Peptide ligand	60
Water/ions	45/-
**RMSD from ideal geometry**	
Bond length (Å)	0.005
Bond angle (º)	0.73
**Ramachandran Statistics (%)**[h]	
Favored	94.9
Outliers	0.2

[a]Parentheses refer to outer shell statistics.

[b]$R_{sym} = \Sigma_{hkl} \Sigma_i | I_{hkl,i} - <I_{hkl}> | / \Sigma_{hkl} \Sigma_i I_{hkl,\mathrm{i}}$, where $I_{hkl,i}$ is the scaled intensity of the ith measurement of reflection *h, k, l*, and $< I_{hkl}>$ is the average intensity for that reflection.

[c]$R_{pim} = \Sigma_{hkl} (1/(n-1))^{1/2} \Sigma_i | I_{hkl,i} - <I_{hkl}> | / \Sigma_{hkl} \Sigma_i I_{hkl,i}$, where n is the redundancy.

[d]$CC_{1/2}$ = Pearson Correlation Coefficient between two random half datasets

[e] Value in parentheses refer to number of reflections in test set.

[f]$R_{cryst} = \Sigma_{hkl} | F_o - F_c | / \Sigma_{hkl} | F_o |$ x 100, where F_o and F_c are the observed and calculated structures factors.

[g]R_{free} was calculated as for R_{cryst}, but on a test set of 5% of the data excluded from refinement.

[h]Calculated using MolProbity[3].

Supplementary Table 7. SPR kinetic data for peptide CP1 binding to influenza A and B HAs.

Influenza A	Lineages	Subtype	Strain	K_D (μM)*
	Group-1	H1N1	A/South Carolina/1/1918	0.035
		H1N1	A/Puerto Rico/8/1934	0.047
		H1N1	A/duck/Alberta/345/1976	0.101
		H1N1	A/New Caledonia/20/1999	0.023
		H1N1	A/Solomon Islands/3/2006	0.128
		H1N1	A/California/07/2009	0.054
		H2N2	A/Adachi/2/1957	>1**
		H5N1	A/Vietnam/1203/2004	1.80
		H6N2	A/turkey/Massachusetts/3740/1965	0.091
		H9N2	A/turkey/Wisconsin/1/1966	>1**
		H11N6	A/duck/England/1/1956	2.30
		H12N5	A/duck/Alberta/60/1976	N.B.
		H13N6	A/gull/Maryland/704/1977	>1**
		H16N3	A/black-headed gull/Sweden/4/1999	1.10
	Group-2	H3N2	A/Hong Kong/1/1968	N.B.
		H3N2	A/Victoria/361/2011	N.B.
		H4N6	A/duck/Czechoslovakia/1956	N.B.
		H7N7	A/Netherlands/219/2003	N.B.
		H10N7	A/chicken/Germany/N/1949	N.B.
		H14N5	A/mallard/Astrakhan/263/1982	N.B.
		H15N9	A/shearwater/W. Australia/2576/79	N.B.
**Influenza B**	**Victoria**	-	B/Brisbane/33/2008	N.B.
	**Yamagata**	-	B/Massachusetts/02/2012	N.B.

* values from a single experiment; ** Binding was observed only for the highest tested concentrations; N.B., no binding; Group-1 HAs: H1/PR8 = A/Puerto Rico/8/1934, H1/SC = A/South Carolina/1/1918, H1/DA= A/duck/Alberta/345/1976, H1/SI = A/Solomon Islands/3/2006, H2/AD = A/Adachi/2/1957, H6/TM = A/turkey/Massachusetts/3740/ 1965, H9/TW = A/turkey/Wisconsin/1/1966, H11/DE = A/duck/England/1/1956, H12/DA = A/duck/Alberta/ 60/1976, H13/GM = A/gull/Maryland/704/1977, H16/BS = A/black-headed gull/Sweden /4/1999. Group-2 HAs: H3/HK68 = A/Hong Kong/1/1968, H3/Vict = A/Victoria/361/2011, H4/DC = A/duck/Czechoslovakia/1956, H7/Neth = A/Netherlands/219/2003, H10/CG = A/chicken/Germany/N/1949, H14/MA = A/mallard/Astrakhan/263/1982, H15/SWA = A/shearwater/W. Australia/2576/79. Influenza B HAs: FluB/Bris = B/Brisbane/33/2008 and FluB/Mas = B/Massachusetts/02/2012.

Supplementary Table 8. **Conformational change inhibition assay (CCI) for peptide ligands.**

Peptide ID	HA strain	IC_{50} (µM)
LP1	H1 A/Brisbane/10/07	>100
CP1	H1 A/Brisbane/10/07	0.049±0
CP2	H1 A/Brisbane/10/07	21.88±3.24
CP3	H1 A/Brisbane/10/07	2.78±0.26
CP4	H1 A/Brisbane/10/07	0.39±0.04
CP5	H1 A/Brisbane/10/07	16.99±4.58
CP6	H1 A/Brisbane/10/07	2.74 ±0.90
CP7	H1 A/Brisbane/10/07	3.54±0.31
CP8	H1 A/Brisbane/10/07	0.54±0.01
CP9	H1 A/Brisbane/10/07	0.23±0.02
CP10	H1 A/Brisbane/10/07	0.46±0.04
CP11	H1 A/Brisbane/10/07	8.52±0.39
CP12	H1 A/Brisbane/10/07	N.D.
CP13	H1 A/Brisbane/10/07	39.35±8.32
CP14	H1 A/Brisbane/10/07	1.24±0.03

N.D.- Not determined; values reported in the table are an average of two independent experiments. The standard deviation of the errors is reported.

Supplementary Table 9. **P-glycoprotein substrate specificity and cell permeability for peptides.**

		LLC-Parent		LLC-MDR1	
**Condition**	**Direction***	**P_{app} (10^{-6} cm/s)**	**Efflux ratio BA/AB**	**P_{app} (10^{-6} cm/s)**	**Efflux ratio BA/AB**
30 nM ^{3}H-Digoxin	AB	2.45±0.10	0.94	0.45±0.08	22.6
	BA	2.31±0.15		10.3±0.20	
30 nM ^{3}H-Digoxin + 5 µM GF120918	AB	2.65±0.12	0.9	2.98±0.15	0.91
	BA	2.37±0.08		2.72±0.05	
5 µM CP14	AB	0.11±0.00	1.17	0.26±0.04	1.23
	BA	0.12±0.01		0.32±0.05	
5 µM CP14 + 5 µM GF120918	AB	0.11±0.01	1.01	0.25±0.01	1.33
	BA	0.11±0.01		0.33±0.05	
5 µM CP1	AB	0.07±0.01	2.55	0.21±0.03	1.57
	BA	0.19±0.09		0.33±0.08	
5 µM CP1 + 5 µM GF120918	AB	0.07±0.01	2.97	0.23±0.04	1.36
	BA	0.22±0.12		0.32±0.02	

LLC-parent and LLC-MDR1(multidrug resistance protein 1) cell lines were used for permeability assay. Condition for permeability assay: membrane- Optimem; incubation time-120 minutes, 1% BSA, pH 7.4; P-gp substrate- ^{3}H-Digoxin; P-gp inhibitor- GF120918 (Elacridar); AB = Apical to basolateral, BA= basolateral to apical. % peptide recovery: 92.9 % (CP1) and 96.5% (CP14). Digoxin acts as a positive control. The standard deviation of the errors is reported.

References

1 Whitehead, T. A. *et al.* Optimization of affinity, specificity and function of designed influenza inhibitors using deep sequencing. *Nat. Biotechnol.* **30**, 543-548 (2012).

2 Krause, J. C. *et al.* Naturally occurring human monoclonal antibodies neutralize both 1918 and 2009 pandemic influenza A (H1N1) viruses. *J. Virol.* **84**, 3127-3130 (2010).

3 Chen, V. B. *et al.* MolProbity: all-atom structure validation for macromolecular crystallography. *Acta Crystallogr. D Biol. Crystallogr.* **66**, 12-21 (2010).
